# Intertwined Dysregulation of Ribosomal Proteins and Immune Response Delineates SARS-CoV-2 Vaccination Breakthroughs

**DOI:** 10.1128/spectrum.04292-22

**Published:** 2023-04-06

**Authors:** Ranjeet Maurya, Uzma Shamim, Pallavi Mishra, Aparna Swaminathan, Aakarshan Raina, Bansidhar Tarai, Sandeep Budhiraja, Rajesh Pandey

**Affiliations:** a Division of Immunology and Infectious Disease Biology, INtegrative GENomics of HOst-PathogEn (INGEN-HOPE) laboratory, CSIR-Institute of Genomics and Integrative Biology (CSIR-IGIB), Delhi, India; b Academy of Scientific and Innovative Research (AcSIR), Ghaziabad, India; c Max Super Speciality Hospital, Max Healthcare, Delhi, India; Huashan Hospital of Fudan University

**Keywords:** COVID-19, vaccination breakthroughs, milder disease severity, ribosomal proteins, transcription factors, immune tolerance

## Abstract

Globally, COVID-19 vaccines have emerged as a boon, especially during the severe pandemic phases to control the spread of severe acute respiratory syndrome coronavirus 2 (SARS-CoV-2) infections, saving millions of lives. However, mixed responses to vaccination with breakthrough challenges provided a rationale to explore the immune responses generated postvaccination, which plausibly alter the subsequent course of infection. In this regard, we comprehensively profiled the nasopharyngeal transcriptomic signature of double-dose-vaccinated individuals with breakthrough infections in comparison to unvaccinated infected persons. The vaccinated individuals demonstrated a gross downregulation of ribosomal proteins along with immune response genes and transcription/translational machinery that methodically modulated the entire innate immune landscape toward immune tolerance, a feature of innate immune memory. This coordinated response was orchestrated through 17 transcription factors captured as differentially expressed in the vaccination breakthroughs, including epigenetic modulators of *CHD1* and *LMNB1* and several immune response effectors, with *ELF1* emerging as one of the important transcriptional regulators of the antiviral innate immune response. Deconvolution algorithm using bulk gene expression data revealed decreased T-cell populations with higher expression of memory B cells in the vaccination breakthroughs. Thus, vaccination might synergize the innate immune response with humoral and T-cell correlates of protection to more rapidly clear SARS-CoV-2 infections and reduce symptoms within a shorter span of time. An important feature invariably noted after secondary vaccination is downregulation of ribosomal proteins, which might plausibly be an important factor arising from epigenetic reprogramming leading to innate immune tolerance.

**IMPORTANCE** The development of multiple vaccines against SARS-CoV-2 infection is an unprecedented milestone achieved globally. Immunization of the mass population is a rigorous process for getting the pandemic under control, yet continuous challenges are being faced, one of them being breakthrough infections. This is the first study wherein the vaccination breakthrough cases of COVD-19 relative to unvaccinated infected individuals have been explored. In the context of vaccination, how do innate and adaptive immune responses correspond to SARS-CoV-2 infection? How do these responses culminate in a milder observable phenotype with shorter hospital stay in vaccination breakthrough cases compared with the unvaccinated? We identified a subdued transcriptional landscape in vaccination breakthroughs with decreased expression of a large set of immune and ribosomal proteins genes. We propose a module of innate immune memory, i.e., immune tolerance, which plausibly helps to explain the observed mild phenotype and fast recovery in vaccination breakthroughs.

## INTRODUCTION

The COVID-19 pandemic, caused by severe acute respiratory syndrome coronavirus 2 (SARS-CoV-2), has had devastating impacts on human lives worldwide, with implementation of containment measures and subsequent vaccination as crucial strategies to control recurrent infections (1). Ten COVID-19 vaccines, synthesized using eight different vaccine development strategies, have been approved by the WHO for global use. The mRNA vaccines have been largely used in the United States and other developed countries, whereas their usage in developing countries has been limited (2). On 31 December 2020, the WHO issued the emergency use authorization for the Pfizer COVID-19 vaccine (BNT162b2), followed by AstraZeneca/Oxford COVID-19 vaccine (3). Further, the administration of vaccines against COVID-19 commenced on 16 January 2021 in India with approved emergency use of ChAdOx1-S/Covishield (viral vector vaccine) and the indigenous vaccine Covaxin (inactivated virus vaccine), with later addition of Sputnik-V (viral vector vaccine) (4, 5). Consequently, vaccination coverage has been rising and more than 2 billion doses, including booster doses, have been administered in India as of September 2022, inclusive of children aged 12 years (https://covid19.who.int/region/searo/country/in; https://www.mohfw.gov.in/). Despite the mass immunization and overall efficacy of vaccines in the randomized controlled settings, several breakthrough infections have been encountered and the number has increased with time in the real world, pushing the target of achieving herd immunity a bit (6–8). Nevertheless, a higher proportion of breakthrough infections were observed to be milder in disease severity, with a reduced mortality rate globally (9). This could be reflective of numerous modulators, including a decrease in vaccine effectiveness and suboptimal immune response in individuals with comorbidities. This reinforces the need to elucidate the factors that are responsible for the breakthroughs for a possibly better immunization regime.

COVID-19 vaccines, like other vaccines, are biological preparations that provide acquired immunity by mimicking specific pathogen molecules (10). Consequently, characterizing the kinetics of differential immune responses elicited by the COVID-19 vaccines will aid us in elucidating both the long- and short-term effects of vaccination and the subsequent episodes of breakthrough infections (11–13). Additionally, the monitoring of the immune response postvaccination could also offer a biomarker-based approach to systems vaccinology. Several studies have reported the infection profiles of individuals fully or partially vaccinated based on transcriptome data. Subsequently, it was reported that the immune response genes were upregulated in the vaccinated group, including the interferon-stimulated genes *OAS1* and *OAS2*, which implied the induction and enhancement of the JAK-STAT pathway upon vaccination by BNT162b2 (14). A study by Fan et al. reported a reduction in the virus-induced inflammation by cytokine profiling in the vaccinated group compared to the unvaccinated (15). Furthermore, Khoury et al. demonstrated a decline in antibody titer in individuals 1 month after the second dose administration of BNT162b2 mRNA vaccine (16). Interestingly, a study reported the effectiveness of BNT162b2 mRNA vaccine against COVID-19-related outcomes in a cohort of 596,618 people in Israel to be consistent with the randomized clinical trial outcomes (17). However, Steensels et al. demonstrated that health care workers (HCW) vaccinated with mRNA-1273 showed higher antibody titers than those vaccinated with BNT162b2 in a Belgium cohort of 2,499 HCW (18). Another study from an eastern state of India demonstrated a significant difference in the seroconversion between Covaxin- and Covishield-vaccinated individuals, with higher seropositivity seen in individuals vaccinated with Covishield (96.7%) than with Covaxin (77.1%) (19).

In addition to the variations in humoral responses observed after vaccination, several studies have reported heterogeneous cellular responses following COVID-19 vaccination (20–22). Recently, a study demonstrated a greater magnitude of changes in interferon response, cytokine expression, T-cell exhaustion, and cytotoxicity in patients with severe COVID-19 than in individuals vaccinated with CoronaVac (23). Moreover, a downregulation of the type I interferon response was observed after vaccination in Vero cell lines in contrast to its reported protection against SARS-CoV-2 infection. Furthermore, a reduction in the lymphocyte-associated gene expression was also seen at least 28 days after the vaccine administration (24). Thus, the diverse immune profile displayed by vaccinated individuals should be examined in depth to optimize vaccine efficacy coupled with reduced COVID-19 reinfections, which would help us achieve population immunity against SARS-CoV-2.

In an effort toward understanding the impact of vaccination in the vaccination breakthroughs, we comprehensively analyzed the transcriptomic profile obtained from the RNA-seq data of 58 patients categorized into two groups: vaccination breakthroughs (VBT) and unvaccinated infected (UNV). Further, to elucidate the master regulators of differentially expressed genes (DEGs) observed between the groups, transcription factor analysis with respect to its target genes (TGs) among the DEGs and subsequent expression correlation among them was carried out. With an aberrant downregulation of genes attributed to the immune response during vaccination breakthroughs, cell-type-specific analysis was performed to delineate the composition of subtypes of cells associated with innate and adaptive immunity following infection postvaccination. Interestingly and importantly, our analysis also highlighted the gross downregulation of ribosomal proteins in vaccination breakthroughs that are the least explored and correlated with vaccination in published literature. Overall, our findings underlined a subdued/suboptimal immune response in the vaccination breakthrough cases that could possibly regulate infection recovery positively.

## RESULTS

### Study design, patient clinical characteristics, and cohort characterization.

**FIG 1:**
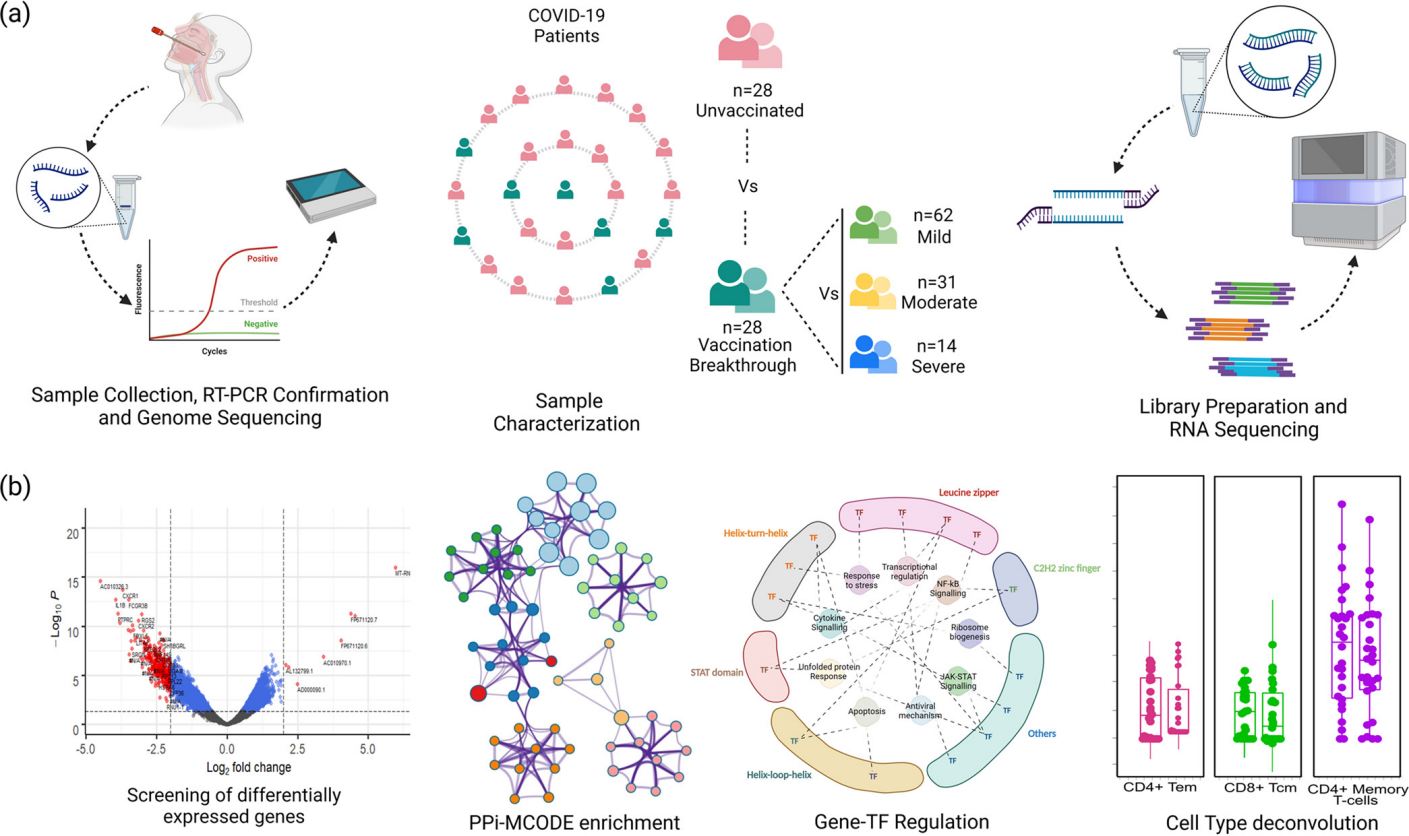
Overview of study design and experimental workflow. (a) Study design illustrating sample collection, patient cohorts, and experimental workflow toward RT-PCR, SARS-CoV-2 genome sequencing, and human host RNA-seq. Highlights the sample cohorts of vaccination breakthrough/unvaccinated and the differential disease severity of mild, moderate and severe. (b) Transcriptomic data analysis followed by screening of differentially expressed genes, downstream functional analyses, and visualizations toward inferences drawn.

The study cohort included 58 hospital-admitted COVID-19 patients who had tested positive by reverse transcription-PCR (RT-PCR) between January and April 2021, the initial Delta variant period in India. These patients had also undergone SARS-CoV-2 whole-genome sequencing using nasopharyngeal RNA. To investigate the vaccination effect during subsequent SARS-CoV-2 infection, the patients were segregated into two groups, i.e., unvaccinated and SARS-CoV-2 infected (UNV; *n* = 29) and vaccination breakthrough (VBT; *n* = 29), wherein the vaccination breakthrough cases had received two doses of COVID-19 vaccine (Covishield) prior to infection. A different cohort of 107 COVID-19 patients (from an earlier time frame, April to May 2020) with differential disease severities (mild *n* = 62; moderate *n* = 31; severe *n* = 14) was also included in the present study, to substantiate the findings of vaccination breakthrough group as well as for focused validation of functionally important disease severity-associated genes (25) in the UNV and VBT group RNA. Figure 1a summarizes our study design demonstrating patient segregation, bulk RNA-seq workflow, and downstream transcriptome analysis. A total of 1,733,841,806 reads were generated for all samples after RNA-seq, with an average of 30,961,460.82 reads per sample passing quality control (see Table S1 in the supplemental material). For stringent analysis and inferences, one sample each from the UNV and VBT groups was removed due to low reads. DGE analysis was done utilizing data for 28 unvaccinated and 28 vaccination breakthrough patients (Fig. 1b). Phylogenetic investigation of SARS-CoV-2 showed dominance of clades 19A and 20A in both unvaccinated (*n* = 28) and vaccination breakthrough (*n* = 15) cases, with clade 21A uniquely associating with only the vaccination breakthroughs (*n* = 13).

**FIG 2:**
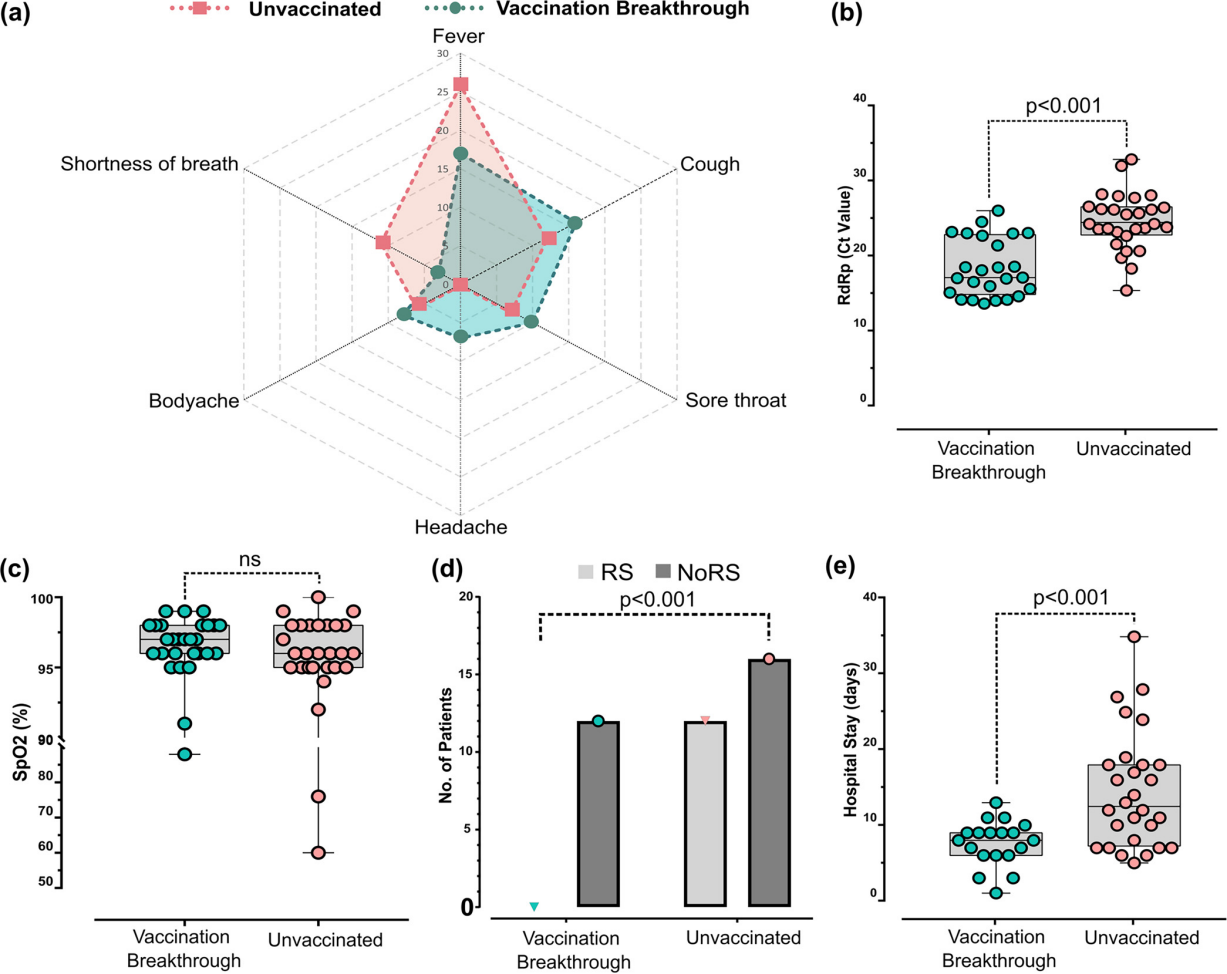
Clinical parameters for the vaccination breakthrough and unvaccinated cohorts. (a) Spider plot capturing various disease symptoms of fever, shortness of breath, body ache, headache, sore throat, and cough between the vaccination breakthrough and the unvaccinated individuals. (b to e) Individual clinical variables have been plotted for *C_T_* value of SARS-CoV-2 RdRp gene (b), SpO_2_ level (c), number of patients requiring respiratory support (RS) (d), and duration of hospital stay (e), with statistical significance measured using Mann-Whitney U test. ns, not significant.

Figure 2 highlights the clinical features of both patient groups, recorded on the day of confirmed SARS-CoV-2 infection and hospital admission. The demographic details and their hospital-captured clinical parameters are outlined in Table 1. There were relatively more male patients in the UNV cohort (7 females versus 21 males), while the VBT group showed near equal gender proportions (16 females versus 12 males). The vaccinated breakthrough individuals were younger, with a median age of 36 years (range, 24 to 56), than unvaccinated infected (median age, 58 years; range, 39 to 63). The VBT patients had received two doses of Covishield vaccine, although after a time gap of 40 days on average (range, 9 to 74), the outbreak infection occurred.

**TABLE 1:** Demographic details and clinical parameters for unvaccinated and vaccination breakthrough cohort of patients

Parameter	Value for cohort		*P* value^*a*^
	Vaccination breakthrough (*n* = 28)	Unvaccinated infected (*n* = 28)	
Gender, F/M^*b*^	16/13	7/21	*0.012*
Age in yrs, median (IQR)	36 (24–56)	58 (39–63)	**<*0.05***
*C_T_* value for RdRp gene, median (IQR)	17 (15.0–22.6)	24.4 (22.9–26.4)	**<*0.001***
SpO_2_, median (IQR)	97 (96–98)	96 (95–98)	*0.153*
Respiratory support, no. (%)	0	12 (42.85)	
Shortness of breath, no. (%)	4 (14.28)	11 (39.28)	**0.033**
Fever, no. (%)	17 (60.71)	25 (89.28)	**0.023**
Cough, no. (%)	17 (60.71)	12 (42.85)	0.243
Sore throat, no. (%)	11 (39.28)	7 (25)	0.223
Body ache, no. (%)	8 (28.57)	5 (17.85)	0.274
Comorbidity, no. (%)	3 (10.71)	20 (71.42)	**<0.001**
Hospital stay in days, median (IQR)	8 (6–9)	12.5 (7.75–18)	**<*0.001***

a Statistical significance was calculated using Mann-Whitney U test (*P* values in italics) or chi-square test (*P* values underlined). Values of significance are in bold.

b F, females; M, males.

The clinical symptoms were of mild presentation in both the UNV and the VBT patients, with fever, cough, sore throat, headache, and body ache distributed similarly (Fig. 2a). SARS-CoV-2 RT-PCR revealed a significantly lower cycle threshold (*C_t_*) value (17.0; range, 15.0 to 22.6) for the RNA-dependent RNA polymerase (RdRp) gene in the VBT patients than in the UNV patients (24.4; range, 22.9 to 26.4) (Fig. 2b). Shortness of breath was present in a relatively higher number of patients in the UNV group (*n* = 11) than in the VBT group (*n* = 4), yet the partial oxygen pressure (SpO_2_ level) measured at the time of hospital admission was within normal range (VBT, median = 97; UNV, median = 96) for both the groups (Fig. 2c). Similarly, a higher proportion of UNV patients (*n* = 20) had comorbid conditions than VBT patients (*n* = 3), a feature that might alter disease manifestation during the hospital stay. Notably, 12 UNV patients required respiratory support during the hospital stay, resulting in a mild+ clinical presentation, whereas none of the VBT patients required respiratory support (Fig. 2d). The hospital stay duration for vaccination breakthrough cases was, on average, 8 days (range, 6 to 9), whereas that of UNV patients was 12.5 days (range, 7.8 to 18) (Fig. 2e). Taken together, these results make it evident that both UNV and VBT patients showed mild COVID-19 manifestations but the recovery process was observed to be faster in the vaccination breakthrough patients.

### Depleted ribosomal and immune signatures in vaccination breakthrough patients.

**FIG 3:**
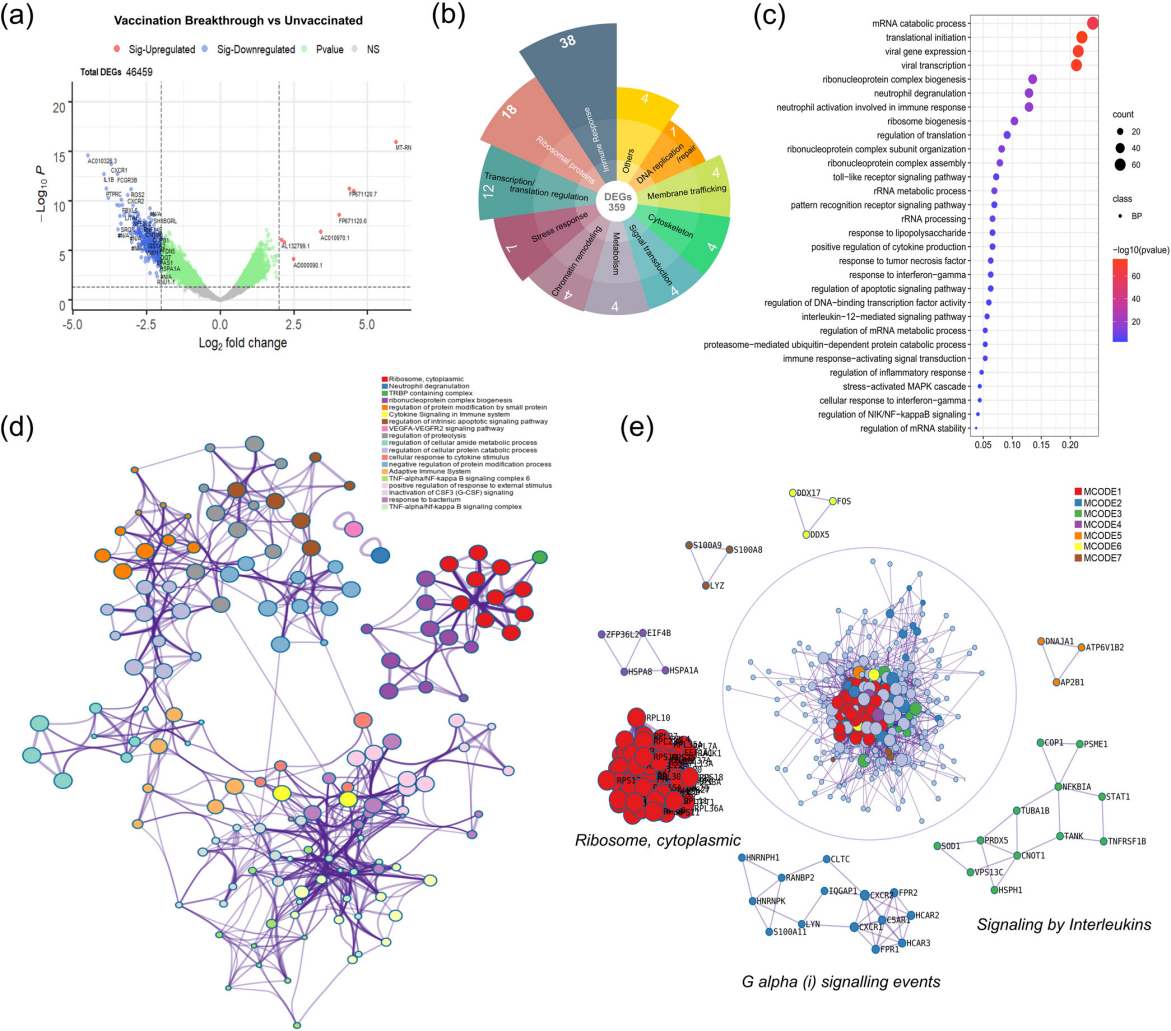
Differential expression, functional enrichment, and interactome analysis in the vaccination breakthrough and unvaccinated cohorts. (a) Volcano plot representing differentially expressed genes (DEGs) highlights genes with log_2_ fold change of ±62 and adjusted *P* value of <0.05. (b) Classification of DEGs under different functional categories. (c) Dot plot visualization of enriched pathways from the DEGs. (d) PPi enrichment network visualization among the DEGs showing the cluster similarities of enriched terms, taking 10 terms per cluster into consideration. Cluster annotations are shown in color code. (e) Module detection from the PPi interactome using MCODE clustering. Circles represent all the protein nodes. Nodes in each subgraph are colored differently for specific modules.

To identify the transcriptional profile of vaccination breakthroughs, we performed comparative analysis of RNA-seq data between the VBT and the UNV patients. We obtained 359 significant DEGs, of which 8 were upregulated and 351 were downregulated, in the vaccination breakthrough patients (Fig. 3a; Table S2). We classified the DEG set of vaccinated individuals under different functional categories. Immune response genes comprised highest proportion (38%; *n* = 136), followed by ribosomal protein genes (18%; *n* = 64) and genes regulating transcription/translation machinery (12%; *n* = 43). The other genes, invariably constituting the remaining 32% (*n* = 114), belonged to stress response, chromatin remodeling, and signal transduction, as shown in Fig. 3b. Subsequently, we did gene ontology (GO) enrichment analysis for the DEGs to identify pathways regulated by these genes (Table S3). The top 30 significant pathways are plotted in Fig. 3c. The genes for ribosome protein and transcription/translation regulatory genes were highly enriched for pathways governing ribosome biogenesis, mRNA and protein catabolic processes, translation initiation, and ribonucleoprotein complex biogenesis along with viral gene expression and viral transcription. The pathways captured for the immune genes were basically regulators of the innate immune response, such as neutrophil activation and degranulation, Toll-like receptor (TLR) and pattern recognition receptor signaling, response to tumor necrosis factor, response to interferon gamma, and positive regulation of cytokine production. Since the DEG set was majorly downregulated, we can consider that these pathways signaled a subdued immune and inflammatory response which seems to be resultant of coordinated downregulation of ribosomal proteins and transcription/translational machinery.

Further, protein-protein interaction (PPi) enrichment network of downregulated DEGs was established using STRING and the BioGrid database, highlighting pathways and interactions between them (Fig. 3d). The top interacting pathways were of ribosomes, the TRBP (TRE RNA-binding protein)-containing complex, and ribonucleoprotein complex biogenesis. Enrichment of these pathways reflect a role in gene expression silencing during cellular stress. The other set of interacting pathways included that of regulation of intrinsic apoptotic pathways along with protein modification, proteolysis, and cellular protein catabolic processes, wherein apoptotic pathways are suggested to play a role in the maintenance of immune tolerance. The innate and adaptive immune system pathways constituted yet another interacting network. Using the decomposition function (k-score of 2) of Molecular Complex Detection (MCODE), 7 modules were obtained representing a small set of DEGs for each cluster (Fig. 3e). It is hypothesized that genes which play a significant role in the vaccination breakthrough cohort included ribosomes, i.e., ribosomal proteins, genes regulating immune signaling by the interleukins (immune responses through inflammation) and G alpha signaling (role in lymphocyte development, immune cell survival and migration, and/or tolerance induction), which collectively represent the innate and adaptive branches of immune response.

### TFs as key players for a mutually and coherently modulating large set of genes associated with multiple pathways.

**FIG 4:**
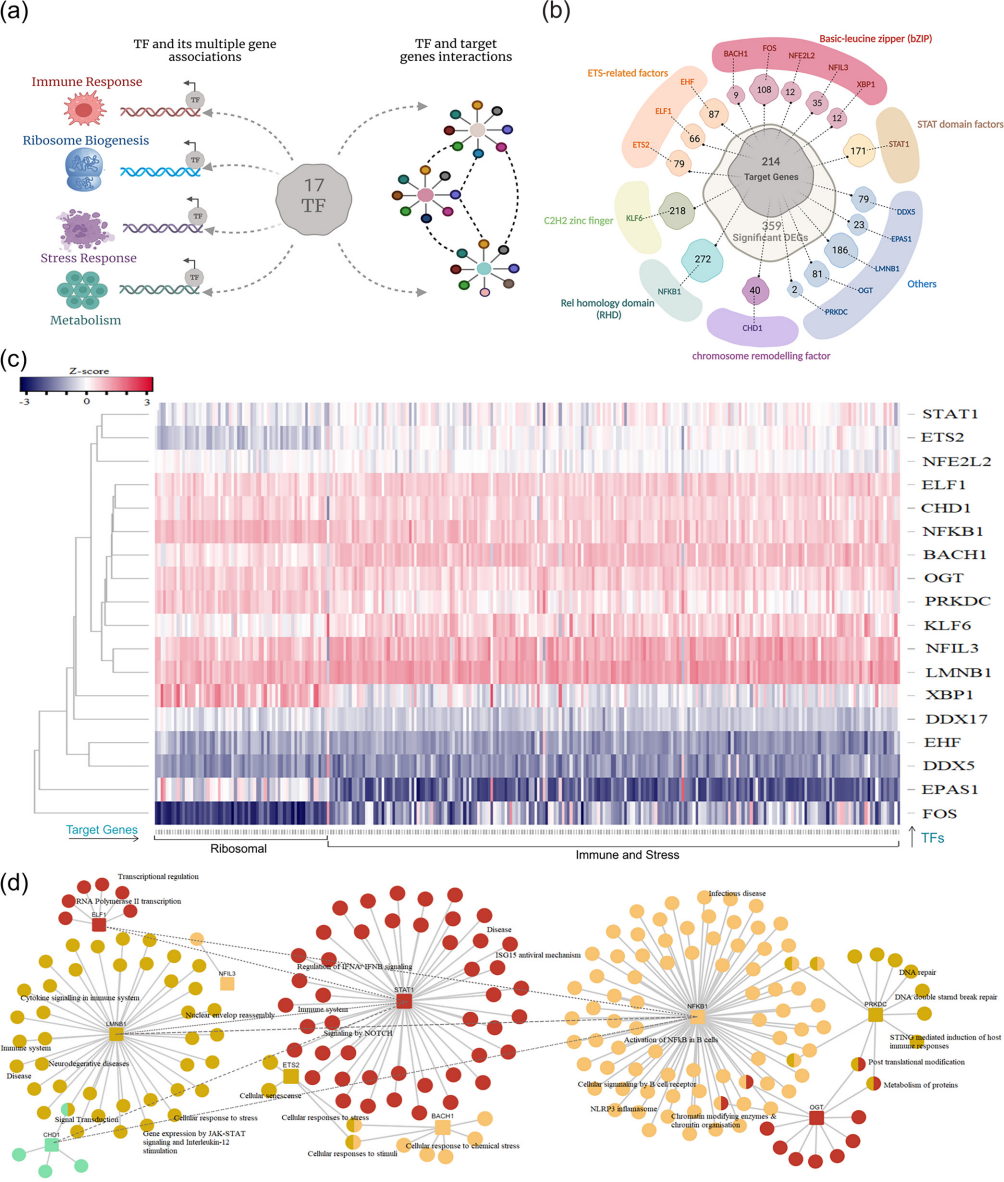
Coexpression and functional elucidation of transcription factors (TFs). (a) Illustration of TF regulation of different components of DEGs from the vaccination breakthrough and unvaccinated cohorts. (b) Number of target genes associated with TFs from the DEGs. (c) Heat map representing Pearson correlation coefficient between differentially expressed TFs and target genes. (d) Pathway enrichment of TFs regulating the ribosomal and the immune components together as well as differentially associated with ribosomal and immune response genes.

Our previous result consequently led us to identify transcription factors (TFs) with predicted target genes showing gross modulation in the VBT individuals compared to the unvaccinated ones (Fig. 4a). Several TFs are likely to play significant roles in the induction of innate and adaptive immunity in response to the vaccination. Remarkably, 17 transcription factors were determined through the databases TRANSFAC (TRANScription FACtor database) and hTFtarget (Database of Human Transcription Factor Targets) that were differentially expressed in the gene set of the vaccination breakthrough cohort. These covered six structural superclasses of TFs carrying the following basic domains: stat domain factor, *STAT1*; basic leucine zippers, *BACH1*, *FOS*, *NFE2L2*/*NRF2*, *NFIL3*, and *XBP1*; ETS-related factors/helix-turn-helix, *EHF*, *ELF1*, and *ETS2*; C2H2 zinc finger, *KLF6*; REL homology domain, *NFKB1*; and chromosome remodeling factor, *CHD1*. *DDX5*, *EPAS1*, *LMNB1*, *OGT*, and *PRKDC* did not belong to any superclass (Fig. 4b). We extracted the target genes of these 17 TFs and found that each TF had certain number of target genes present in the significant DEG set of the VBT group (Table S4). In totality, 214 out of the 359 DEGs constituted the target genes for the 17 TFs. Since the 214 target genes substantially overlapped for the 17 TFs, we wanted to elucidate the expression correlation between the two. The correlation matrix is plotted as a heat map in Fig. 4c. As is evident, the TFs segregated into three clusters, with positively correlated TFs such as *ELF1*, *CHD1*, *NFKB1*, *BACH1*, *OGT*, *PRKDC*, *KLF6*, *STAT1*, *NFIL3*, and *XBP1*. The TFs showing negative correlation included *DDX17*, *DDX5*, *EHF*, *EPAS1*, and *FOS*, while *NFE2L2* and *ETS2* showed no correlation.

Moreover, the target genes too showed differential expression with different TFs, forming two gene clusters, one of ribosomal proteins and the other of immune regulatory genes. Subsequently, it was observed that *LMNB1*, *NFIL3*, *ELF1*, and *CHD1* equally regulated both the target gene components, whereas *BACH1*, *KLF6*, *STAT1*, and *ETS2* showed enhanced expression correlation with immune regulatory genes, in addition to *PRKDC*, *NFKB1*, *OGT*, and *XBP1* with ribosomal protein genes. *FOS* and *EPAS1* negatively correlated with the ribosomal protein and the immune regulatory genes, respectively. Next, pathway analysis for the three sets of TFs depicted cross regulation of multiple pathways through different TFs and their associated target genes (Fig. 4d).

*ELF1* reportedly regulates a vast transcriptional innate immune program against DNA as well as RNA viruses, involving large numbers of antiviral genes, distinct from the type I interferon response (26). Although *ELF1* is known to regulate antiviral genes independent of the canonical interferon response, it shares pathways with *STAT1* and *NFKB1* (Fig. S1) along with *KLF6* and *FOS* (26). Moreover, *ELF1* also transcriptionally regulates the adaptive branch of the immune response through *BTG1* and *BTG2* (present in our DEG set) by lowering mRNA amounts through mRNA turnover in the naive T cells (27). *NFIL3* is reportedly involved in circadian regulation of gene expression, including rhythmic histone modifications, RNA polymerase II recruitment, circadian chromosomal conformation interactions, and posttranslational modifications (28). Moreover, it is expressed in immune cells and required for differentiation and development of innate lymphoid cells (ILCs) that limit infection at the mucosal surfaces (29). *LMNB1* regulates multiple pathways associated with epigenetic regulation (chromatin organization), signal transduction, immune system, apoptosis, and cellular stress response (alters translational processes, ribosomal proteins, and protein targeting). One of its functions pertaining to immune response is to regulate inflammation in different immune cell types through cytokine and interleukin signaling (*CAPZA1*, *GSTA2*, *HNRNPF*, *LCP1*, *MSN*, *SOD1*, *B2M*, *CAPZA1*, and *CD44*).

*STAT1*, the master regulator of the interferon response, demonstrated multiple pathways, wherein several genes enriched for the pathways were present in our DEG set. The pathways along with their corresponding TFs and associated genes are provided in Table S5. *NFKB1* functions as the repressor of NF-κB, the most important TF regulating cells of the innate immune system, via multiple mechanisms that involve O-GlcNAcylation (via OGT) of its specific subunits. *OGT* and *PRKDC* together regulate the pathways of posttranslational modification and metabolism of proteins. *XBP1* (regulator of unfolded protein response pathways) has been reported to protect the host from the detrimental effects of the innate immune response and inflammation (30). Together, *NFKB1*, *OGT*, *XBP1*, and *PRKDC* might aid in a balanced innate immune response through ribosomal protein regulation.

### Immune cell subtype distribution between the vaccinated and the unvaccinated groups from the bulk RNA-seq data.

**FIG 5:**
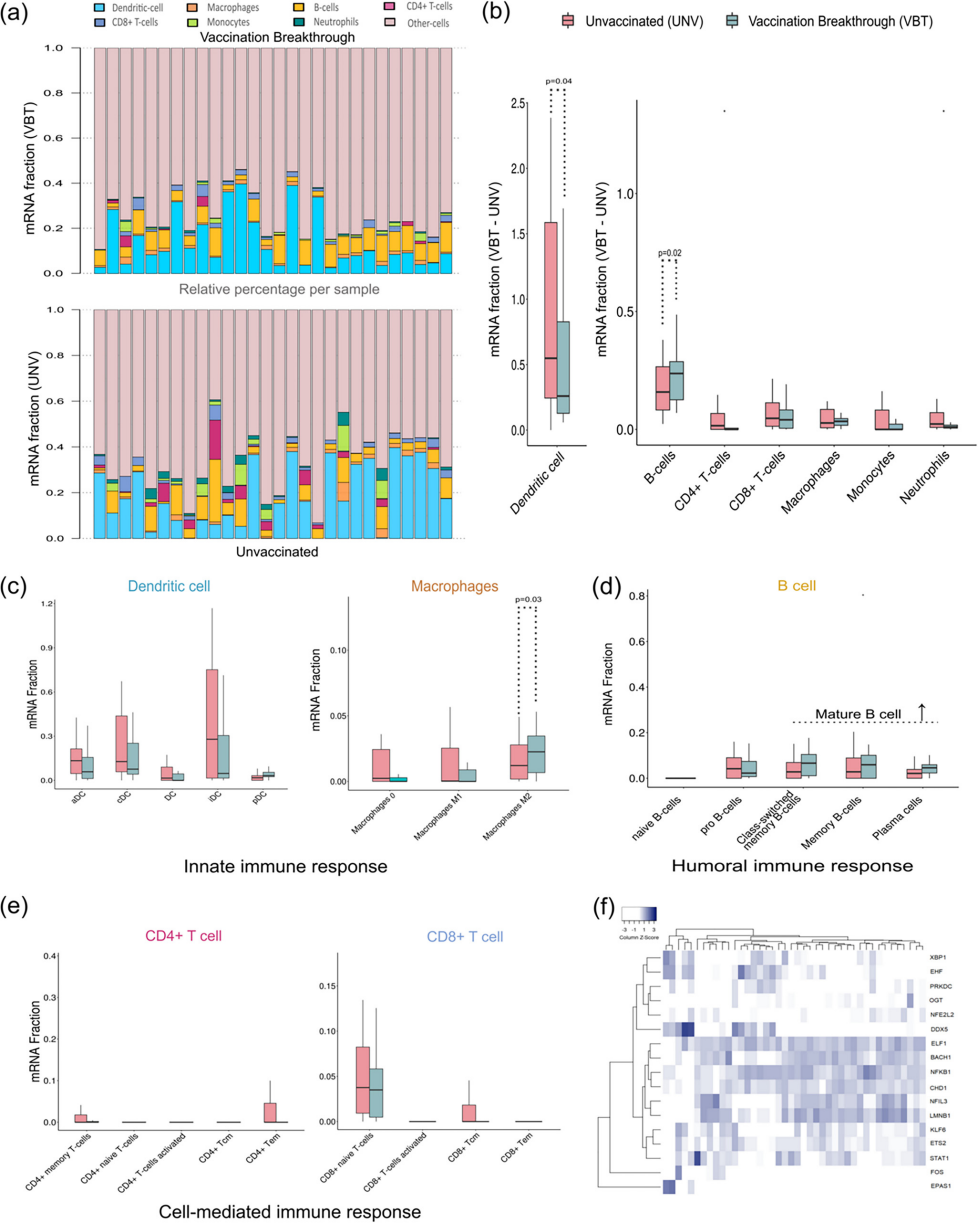
Profiles of immune cell subtype distribution pattern in the vaccination breakthrough and unvaccinated cohorts. (a) Bar plot visualization for the relative proportions of 7 immune cells across all the sample. (b) Box plot of seven specific immune cells with differential infiltrated fraction. (c to e) Box plots for differential infiltrated fraction of dendritic cells and macrophage subtypes (c), B-cell subtypes (d), and CD4^+^ and CD8^+^ T-cell subtypes (e). (f) Correlation heat map of 42 selective marker genes specific to cell types with 17 TFs showing possible correlation of TFs with cell type.

Bulk transcriptome signatures could reveal alterations in transcriptional activity within cells as well as changes in cell composition. To understand possible pattern of immune-related cells and regulators in the cell type(s), the deconvolution algorithm was used for bulk gene expression data for the vaccinated and the unvaccinated groups. This transformed the normalized gene expression matrix into a composition of infiltrating immune cells and abundance of specific cell types. The histogram (Fig. 5a) shows abundance ratios of 7 types of immune cells in each sample, represented by different colors. The height of each color represents the percentage of such cells in the sample, and the sum of the percentage of immune cells is 1. In total, 64 different cell types were captured, including subtypes (Table S6), from which 7 specific types of immune cells (dendritic cells [DCs], macrophages, CD4^+^ and CD8^+^ T cells, B cells, monocytes, and neutrophils) were compared for differential infiltrated fraction (Fig. 5a). Secondary vaccination reportedly increases signatures of dendritic cell activation, monocytes, and neutrophils (31, 32). Interestingly, DCs were found to be significantly lower (Fig. 5b and c) and B cells were found to be significantly higher in the vaccination breakthrough individuals (Fig. 5b and d). DC cells function to activate the T-cell-mediated immune response, which, in turn, activates adaptive immunity. Notably, the abundances of both T-cell populations were low in the VBT group, yet the presence of naive CD8^+^ T cells compared to other T-cell lineages directs toward development of adaptive immune response (Fig. 5b and e). However, we observed a higher abundance of mature B cells, which are responsible for providing the humoral immunity, in the VBT group (Fig. 5d). This possibly relates to vaccination, where we observe higher abundance of mature B cells at an early phase of infection albeit a suboptimal innate immune response. We also observed a higher abundance of macrophages (Fig. 5b), especially M2 macrophages, in the VBT group (Fig. 5c). M1 macrophages are involved in proinflammatory functions, whereas M2 macrophages demonstrate protective functions, including anti-inflammatory, DNA damage, and tissue repair functions (33). Therefore, a higher abundance of M2 macrophages in the vaccinated group indicates better protection against infection, which possibly helps maintain milder symptoms despite the presence of higher viral loads in the VBT group.

Subsequently, the relevance among different immune cells and TF was evaluated using Pearson correlation. For further analysis toward correlation between cell-specific marker genes expression and 17 TFs, we selected 42 marker genes present in our target genes (Table S4). Pearson test indicated that expression of *ELF1* was significantly correlated in the majority of immune infiltration with DCs, CD4^+^ and CD8^+^ T cells, macrophages, neutrophils, and monocytes (Fig. 5f). For T cells and macrophages, other TFs of *BACH1*, *NFKB1*, *CHD1*, *NFIL3*, and *LMNB1* were also correlated. For monocytes, *BACH1*, *NFIL3*, and *LMNB1* were also found along with *ELF1*. *BACH1* is known to be highly expressed in subsets of monocytes, macrophages, neutrophils, and dendritic cells, where decreased expression of *BACH1* results in M2 macrophage differentiation (34), as evidenced in our study. The only TF that showed significant correlation for B cells, *NFKB1*, may have regulatory functions in B cells. The results suggest that DCs emerge as a promising factor to delineate the classical cell-mediated immune response.

### Downregulated ribosomal protein genes in the VBT group may have functional concordance with mild disease severity across cohorts.

**FIG 6:**
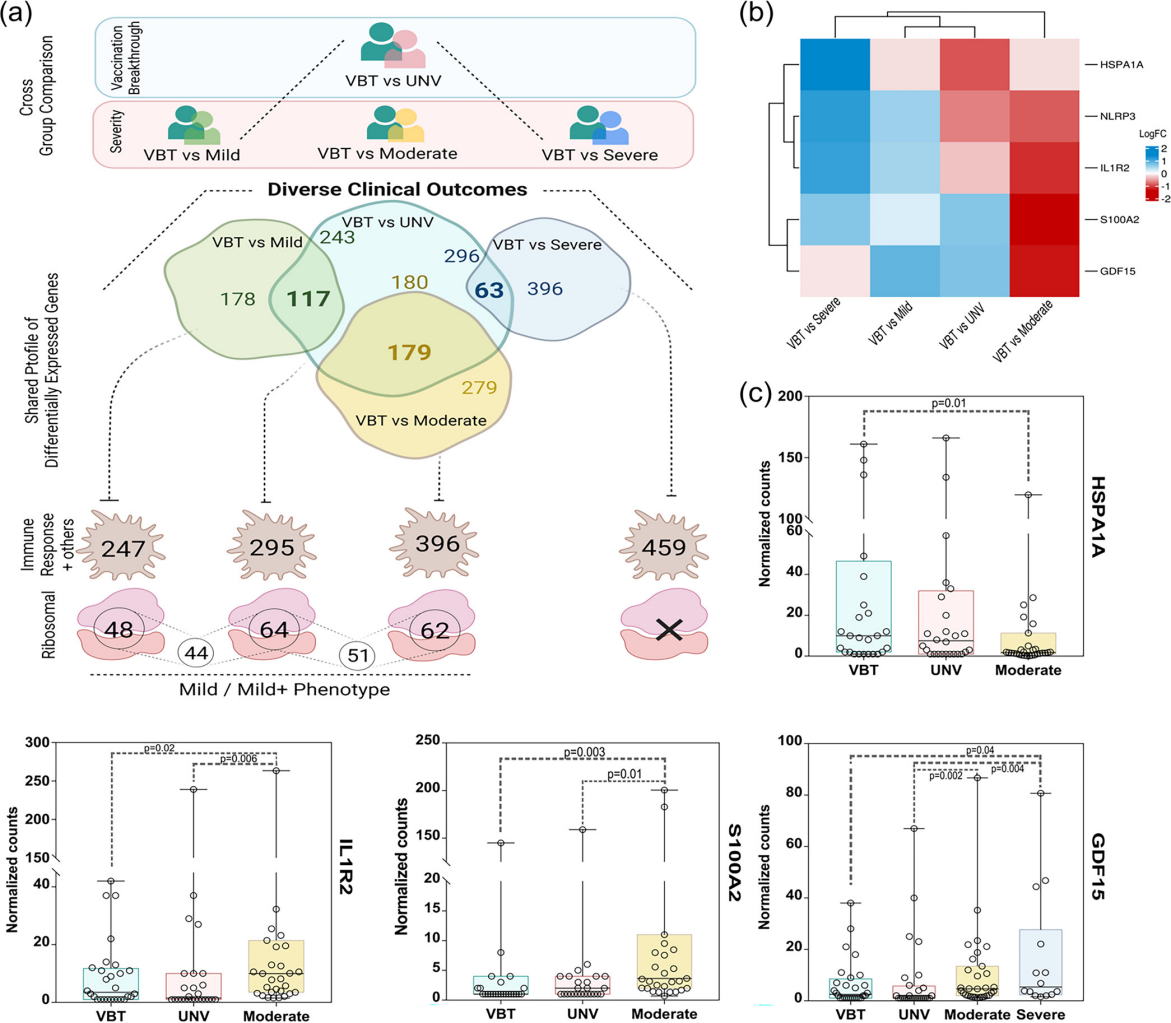
Transcriptomic profile comparison and validation of the specific immune genes for functional concordance across the two study cohorts. (a) Venn diagram representing summary of shared/unique DEGs, presence of immune response, and ribosomal genes across the two-cohort group comparison between the vaccination breakthrough and disease severity subphenotype classification. (b) Heat map representing expression profile (using log_2_ fold change) of key marker genes for severity subphenotype classification from previous study cohort. (c) Box plot of key gene expression showing significant presence.

For broader relevance of the findings presented in this study, we elucidated vaccination breakthrough cases (mild disease presentation) through another cohort of a different time frame with diverse COVID-19 disease severities (mild, moderate, and severe) (Fig. 6a). Differential gene analysis between the VBT versus the mild group, VBT versus the moderate group, and VBT versus the severe group revealed 295, 458, and 459 DEGs, respectively. Next, we compared DEGs for each group with the DEGs obtained for the VBT versus the UNV group. Remarkably, a substantial number of genes were found to overlap between the groups, as evident in the Venn diagram in Fig. 6a, with significant presence of ribosomal protein genes among the VBT versus the mild group (*n* = 48) and VBT versus the moderate group (*n* = 62), compared to the VBT versus the UNV group (*n* = 64). Thus, we can consider that ribosomal protein genes are possibly associated with the vaccination cohort compared with mild and mild+ phenotypes. The inherent presence of ribosomal protein genes in the VBT group implies an important functional outcome of vaccination which needs to be coherently deciphered.

Furthermore, we also investigated the gene profiles of the current VBT and the UNV groups to those of COVID-19-infected patients from an earlier published work from the lab with differential disease severities of mild, moderate, and severe. It is important to note that although the vaccination component was earlier missing from the study groups, the functional importance of the findings in terms of genes associated with disease severity from the earlier study was validated in the present cohort. In the moderate cohort of the earlier study, the genes *S100A2* and *NLRP3* positively regulated the innate immune response, while *HSPA1A* and *IL1R2* were observed to suppress the immune response, thereby indicating a balanced innate immune state in the moderate patients (25). Similarly, *GDF15* was upregulated in moderate as well as severe patients. Exploration of these genes in the mild versus the VBT group, the moderate versus the VBT group, and the severe versus the VBT group revealed similar patterns of differential expression (Fig. 6b), suggesting concordance with earlier reported data. We also looked into individual expression (normalized counts) of these genes in the present cohort of VBT and UNV patients compared with expression in the previous data sets for mild, moderate, and severe disease (Fig. 6c). *IL1R2* and *S100A2* were significantly higher in the moderate group, followed by UNV and VBT, suggesting a subdued innate immune response in the VBT group. Moreover, a significantly higher expression of *HSPA1A* in the VBT group substantiated a regulated immune response in the same. Similarly, GDF15, a marker of COVID-19 severity, was minimally expressed in VBT and UNV groups, followed by increasing trend in moderate and severe cases. Together, these data validate the importance of these genes in regulating the immune response in COVID-19 disease.

## DISCUSSION

We attempted to elucidate through this study the immune characteristics observed in individuals who turned COVID-19 positive despite being double-dose vaccinated and the role that vaccination plays in manifesting a milder phenotype of COVID-19 leading to fast recovery in vaccination breakthroughs.

Several studies have been published that highlight the immune or systemic response following vaccination against pathogens (35–37). Detailed systems-level analysis of innate and adaptive immunity to COVD-19 mRNA vaccine BNT162b2 has been published wherein interferon signaling, dendritic cell activation, and inflammatory responses with a cytokine feedback loop regulated the enhanced innate immune responses to secondary vaccination (31). Yet whether epigenetic reprogramming underlines the enhanced interferon-stimulated gene response after secondary immunization was yet to be explored. Moreover, live attenuated yellow fever vaccine (YF17D), considered one of the most successful vaccines, elicited strong interferon and innate antiviral responses through master transcription factors *STAT1*, *IRF7*, and *ETS2*, which regulated both innate and adaptive effector arms of the immune regulation (38).

Our study also demonstrated a gross modulation of immune response genes that methodically regulated several innate immune pathways in the vaccination breakthroughs (Fig. 3b). In addition, ribosomal proteins, transcription/translational machinery, and protein catabolic processes participated in maintaining the desired immune responses. This coordinated response was orchestrated through several transcription factors and their highly integrated downstream target genes controlling induction of several pathways simultaneously (Fig. 4d). *CHD1*, *LMNB1*, and *ELF1* has been identified as major TFs governing both expression of immune response genes and ribosomal proteins and were also found to be associated with different immune cell types in our data set. *CHD1* and *LMNB1*, as epigenetic regulators, might be responsible for the complex functional reprogramming of innate immune responses, a hallmark/definitive feature observed in the vaccination breakthrough cases. Additionally, we have identified *ELF1* as a major transcriptional regulator of the innate immune response in the vaccinated individuals; *ELF1* is known to elicit a vast antiviral program involving hundreds of genes distinct from those regulated by the interferons. Recently, *ELF1* has been recognized as a novel regulator of inflammation and innate antiviral immunity in the epithelial cells, which provides a delayed antiviral response as it comes into action after multiple rounds of viral replication. This finding sheds importance on the fact that COVID-19 mRNA vaccines are not able to generate a robust type I interferon response, as evidenced during a natural SARS-CoV-2 infection, and bypasses the interferon-mediated pathways to develop a robust adaptive immune response (39).

Cell type analysis was also in concordance with single-cell genomics studies in COVID-19 patients. A majority of the studies have reported a heightened adaptive immune response especially in moderate cases of COVID-19, with expanding CD4^+^ and CD8^+^ cell populations, in comparison to severe cases, which show T-cell dysregulation and significantly lower levels of CD8^+^ and CD4^+^ T cells, B cells, and NK cells (40, 41). In this study, we found comparatively higher populations of dendritic cells and CD4^+^ and CD8^+^ T cells in unvaccinated SARS-CoV-2-infected individuals than in vaccinated individuals. This could be attributed to mild++ (moderate) clinical manifestation in unvaccinated compared to milder phenotype in vaccinated individuals. These findings are supported by an elaborate review by Qi et al., who conducted a single-cell analysis of the adaptive immune response to SARS-CoV-2 infection and vaccination (42). Moreover, expansion of the B-cell population in vaccinated individuals coincides with the single-cell profiling carried out following vaccination by BNT162b2 vaccine (22).

Interestingly, the entire transcriptomic profile examined in our study showed gross downregulation of all the DEGs, be they immune response genes or ribosomal proteins, along with genes related to transcription/translational machinery in the vaccination breakthroughs. An unexplored area is whether COVID-19 vaccination can lead to generation of innate immune memory as witnessed for selected vaccines such as bacillus Calmette-Guérin (BCG). This may be extremely pertinent to COVID-19, where dysregulated inflammation is a major factor in the development of severe illness. Long-term innate immune responses can be either enhanced (potentiation/trained immunity) or suppressed (innate immune tolerance) after certain vaccines or infections (43–46).

Moreover, downregulation of ribosomal proteins is another important aspect noted in our vaccination breakthrough cases, which seamlessly shows association with vaccination (Fig. 6a). Although ribosomal proteins are known to have an essential role in the ribosome assembly and protein translation, their involvement in ribosome-independent functions such as tumorigenesis, immune signaling, and development has been also explored (47–49). Additionally, ribosome machineries are also preferentially modulated by the viral pathogens during virus infections. Notably, studies have reported that in dengue virus infection, the silencing or the knockdown of ribosomal proteins RPL18 and RPLP1/2 significantly reduced the viral replication and translation as well as the viral yield, indicating the importance of ribosomal proteins in the virus life cycle (50, 51). In addition to the virus hijack of host ribosome machinery, studies have elucidated antiviral roles of ribosomal proteins. For instance, a study by Guan et al. demonstrated that foot-and-mouth disease virus (FMDV) 3C^pro^ interacts with RPL13 to reduce its expression, thus suppressing RPL13- mediated antiviral activity (52).

**FIG 7:**
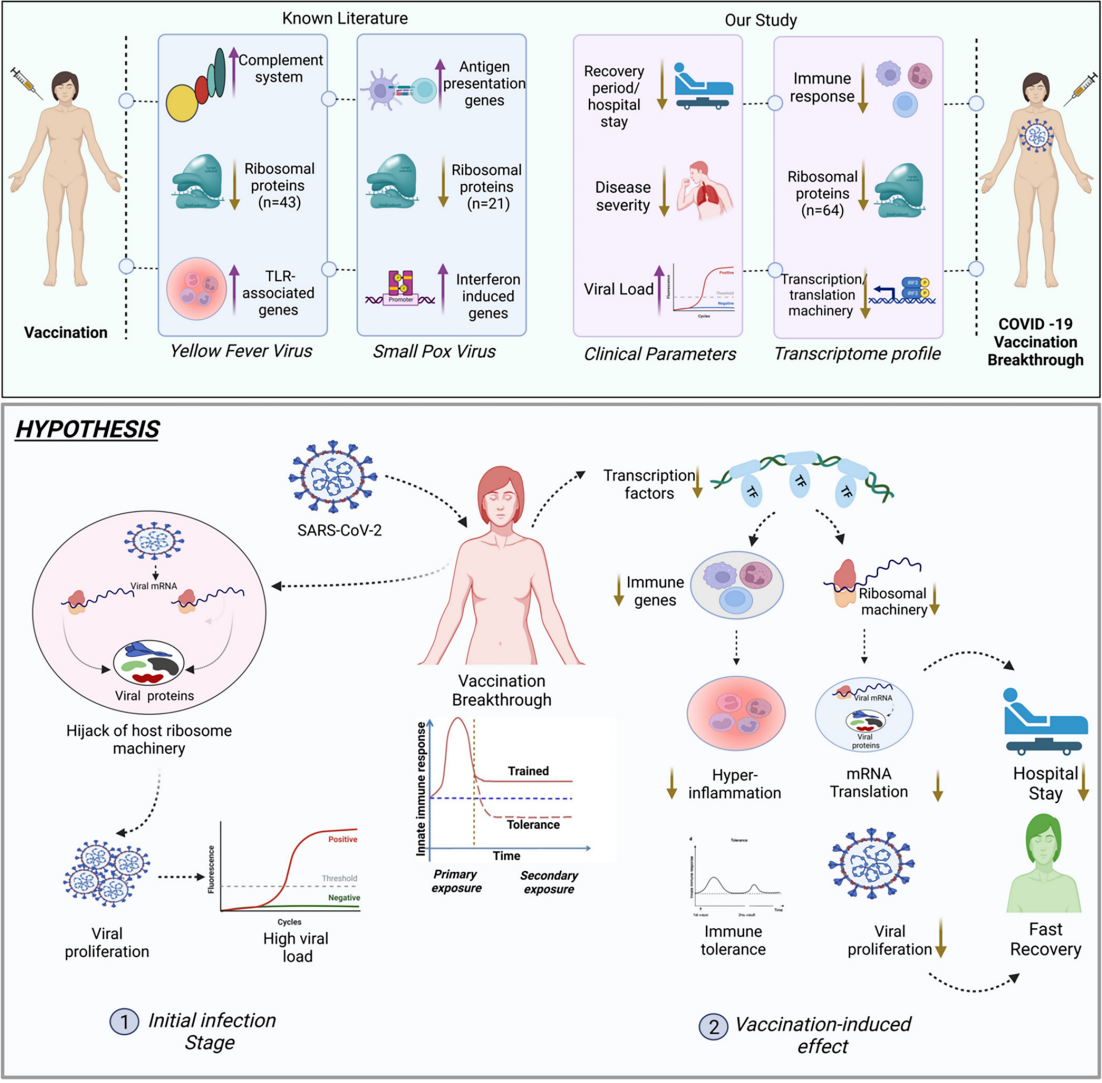
Schematic presentation of summary, possible mechanism for vaccination breakthroughs and milder symptoms. (Top) Concordance of findings from COVID-19 vaccination breakthrough with yellow fever and smallpox vaccination studies reflecting consensus presence of deregulated ribosomal genes. (Bottom) Working hypothesis proposing a possible mechanism for vaccination-induced effect on host immune response associated with vaccination breakthroughs.

Strikingly, there is a paucity of information on the downregulation of ribosomal proteins following immunization with any vaccines. A study by Gaucher et al. reported a downregulation of 43 ribosomal proteins in peripheral blood mononuclear cells (PBMCs) after vaccination with YF17D, the yellow virus vaccine (38). Moreover, downregulation of 21 ribosomal proteins was also reported following vaccination with the smallpox vaccine, vaccinia virus (53). But interestingly, while the classic immune response genes associated with complement system, antigen presentation, interferon induced genes and TLR-associated genes were found to be upregulated following vaccination by YF17D and vaccinia virus, we observed a complete downregulation of immune response genes in the vaccination breakthrough cases compared with the unvaccinated infected individuals (Fig. 7, top). This could possibly imply that vaccine-induced reprogramming of the innate immune response is toward immunotolerance, a feature of innate immune memory that is reported to occur through metabolic and epigenetic reprogramming of the immune cells (altering the landscape of chromatin in gene regions important for innate immune responses) (54, 55).

Threading together the findings of our study, we propose a hypothesis wherein the epigenetic modulation can lead to downregulation of diverse transcription factors and ribosomal proteins directing the immune response toward tolerance (Fig. 7, bottom).

During the initial infection stage, SARS-CoV-2 can proliferate inside the host by hijacking the host ribosomal machinery, leading to detectable high viral loads in the vaccinated individuals. Subsequently, the vaccination-induced effect leads to immune cells being unable to activate immune genes transcription through downregulation of master transcription factors, ribosome proteins, and translation and transcription machineries that could combinedly give rise to a suboptimal systemic inflammatory response leading to immunoparalysis/immunotolerance. Simultaneously, a gross downregulation of ribosomal protein could possibly imply that the viral hijack of ribosome machinery for efficient viral replication is distorted, which leads to decreased viral loads with time and speedy recovery with mild symptoms. We propose that the transcriptomic profile of vaccination breakthrough cases aids in establishing suboptimal systemic response dynamics which might lead to an inhibition of disease progression resulting in a faster recovery with shorter hospital stay as indicated by the patient clinical records.

### Conclusion.

**FIG 8:**
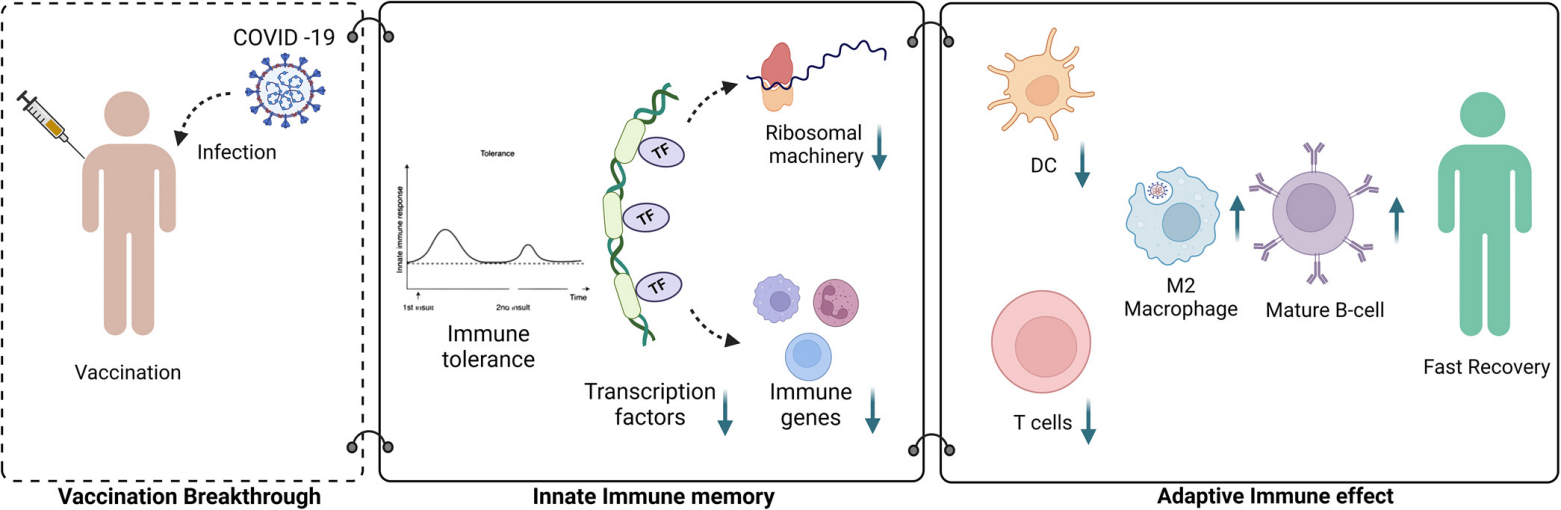
Summary of the study highlights. Vaccination breakthroughs demonstrate trained innate immunity with humoral and T-cell correlates of protection to more rapidly clear SARS-CoV-2 infections, leading to fast recovery.

This is the first study wherein the transcriptional signature in the vaccination breakthrough cases of COVD-19 relative to that in unvaccinated infected individuals has been explored. In the context of vaccination, how do innate and adaptive immune responses correspond to SARS-CoV-2 infection? How does these responses culminate into a milder observable phenotype with shorter hospital stay in vaccination breakthrough cases than for the unvaccinated? The answers lies in the downregulated transcriptional landscape observed in the vaccination breakthrough cases involving transcription factors, modulators of transcription/translation machinery, ribosomal proteins, and immune response genes, together facilitating an optimal or subdued immune response capable of protecting against SARS-CoV-2 infection without eliciting a hyperinflammatory immune response, which is a hallmark of COVID-19. This could possibly be regulating their infection recovery positively. An important feature observed in the vaccinated cases is downregulation of ribosomal proteins, which invariably might be an important factor leading toward innate immune tolerance. Thus, as depicted in Fig. 8, vaccination might synergize trained innate immunity with humoral and T-cell correlates of protection to more rapidly clear SARS-CoV-2 infections and reduce symptoms within a short span of time.

## MATERIALS AND METHODS

### Patient recruitment, sampling, and data collection.

The patients were admitted to a tertiary care center (Max Super Speciality Hospital, Delhi, India) with confirmed COVID-19 based on RT-quantitative PCR (qRT-PCR) from January to April 2021. Nasopharyngeal swabs were collected in Viral transport medium (VTM) by paramedical staff on the day of hospital admission. Viral RNA was isolated using a QIAamp viral minikit (Qiagen; catalog no. 52906), and SARS-CoV-2 detection and quantification was performed using a TRUPCR SARS-CoV-2 kit (3B BlackBio Biotech India Ltd.; catalog no. 3B304). Sequencing of the SARS-CoV-2 genome was performed using an Illumina COVIDSeq kit (catalog no. 20043675) as per the manufacturer’s reference guide (number 1000000126053v04). The demographic and clinical details along with COVID-19 vaccination history of the patients were collected from the electronic health record (EHR). The individuals were segregated into two groups: vaccination breakthrough (infected with SARS-CoV-2 after double-dose vaccination) and unvaccinated (infected with SARS-CoV-2 prior to COVID-19 vaccination).

### RNA sequencing library preparation.

RNA-seq libraries were prepared using Illumina TruSeq stranded total RNA library prep gold (catalog no. 20020599) with 250 ng of RNA isolated from nasopharyngeal swabs of COVID-19 patients, as per the manufacturer’s reference protocol. Cytoplasmic and mitochondrial rRNAs were removed using Ribo-Zero rRNA removal beads. Library preparation included double-stranded cDNA synthesis, adenylation at the 3′ ends, and ligation with index adapters. Subsequently, PCR-based amplification was performed to enrich the cDNA libraries. PCR products were purified using AMPure XP beads and quantified using a Qubit double-stranded DNA (dsDNA) high-sensitivity (HS) assay kit (Thermo Fisher Scientific; catalog no. Q32854). The quality of cDNA libraries was checked using the Agilent 2100 Bioanalyzer. A final loading concentration of 650 pM was used for sequencing, which was performed on the NextSeq 2000, with paired-end 2 × 151 read length.

### Data analysis: quality control, mapping to reference, and identification of differentially expressed genes.

FastQC v0.11.9 was used to determine the quality of raw reads, followed by trimming of adapter sequences using Trimmomatic v0.40. Reads were mapped to the human reference transcriptome (GENCODE) using the Salmon quasimapping tool to quantify transcript read abundance. Differential gene expression analysis was performed using DESeq2 (56). To identify significant differentially expressed genes, Wald’s test with an adjusted *P* value cutoff of ≤0.05, and log_2_ fold change of ≥±2 was applied. Log fold change was plotted against adjusted *P* value using the EnhancedVolcano R package (https://github.com/kevinblighe/EnhancedVolcano).

### PPi enrichment and modular analysis.

For the list of significant combined DEGs, protein-protein interaction (PPi) enrichment analysis was carried out using STRING and BioGrid in Cytoscape v3.9.1. Statistically enriched ontology terms were searched through KEGG, Gene Ontology (GO), Reactome, Hallmark Gene Sets from MSigDB, and CORUM. For each ontology term, a hypergeometric test with Benjamini-Hochberg correction was performed with a corrected *P* value of <0.05. The Molecular Complex Detection (MCODE) algorithm was applied on the resultant PPi to identify densely connected network components using the following criteria: degree cutoff of 2, node cutoff of 0.2, maximum depth of 100, and k-score of 2. The pathway enrichment analysis has been applied to each MCODE cluster, and the best-scoring terms by *P* value were retained as functional description of the corresponding cluster.

### TF and TG identification and coexpression analysis.

Transcription factors (TFs) are important regulators of gene expression. A complete list of transcription factors and downstream target genes (TGs) were identified in combination using TRANSFAC (TRANScription FACtor database) and hTFtarget (Database of Human Transcription Factor Targets) (57). TRANSFAC provides published data on eukaryotic TFs and their experimentally proven regulated genes. From the TRANSFAC database, we pulled a total of 4,316 curated TFs listed with their TF classification among 43 classes and 115 families. The hTFtarget has curated comprehensive TF-target regulations from large-scale of chromatin immunoprecipitation-sequencing (ChIP-seq) data of human TFs (7,190 experimental samples of 659 TFs) under 569 conditions (399 types of cell line, 129 classes of tissues or cells, and 141 kinds of treatments) to predict reliable TF-target regulation and potential association between the TFs. Each TF was classified into particular TF family based on Interpro and Pfam. Further, Pearson correlation analysis between the target gene and TF expression was performed to identify the TG-TF coexpression. A Pearson correlation coefficient value of ≥±0.9 (at a *P* value of ≤0.05), and at least one correlation between the DEG and TF expression was considered significant. The correlation coefficient (*R*) between TG and TF was used for clustering and plotting the heatmap using Heatmapper (58).

### Cell-type enrichment analysis from bulk gene expression profile.

Tissues are complex milieu of cells of different subtypes, each with its own unique and dynamic transcriptomic profile. Bulk transcriptome profiling is therefore the sum of the cell-type-specific gene expression weighted by cell-type proportion in the given sample. The deconvolution of gene expression was performed using xCell for conversion into enrichment scores for 64 immune cell types across the samples (59).

### Statistical analysis and visualization.

Statistical analysis was performed using GraphPad Prism v9.0 and R v4.0.2 available from CRAN or Bioconductor. For quantitative variables, Mann-Whitney-Wilcoxon test was applied to compare the distribution between the two groups. For correlation coefficient, Pearson correlation was performed. Most of the graphs and illustrations were produced using the R package ggplot2 (https://cran.r-project.org/web/packages/ggplot2/index.html) and BioRender.

### Ethics declarations.

The studies involving human participants were reviewed and approved by CSIR-IGIB’s Human Ethics Committee Clearance (reference no. CSIR-IGIB/IHEC/2020-21/01). The patients/participants provided their written informed consent to participate in this study.

### Data availability.

Sequence data used in this study are publicly available in NCBI SRA under BioProject number PRJNA868733.
