# Supplementary material for: Intertwined Dysregulation of Ribosomal Proteins and Immune Response Delineates SARS-CoV-2 Vaccination Breakthroughs
Source: Microbiol Spectr. 2023 Apr 6;11(3):e04292-22. doi: 10.1128/spectrum.04292-22 (PMC10269832; doi:10.1128/spectrum.04292-22)

Supplementary Material

Supplementary table S1: Raw Sequencing Reads information.

**Serial No**	**Sample IDs**	**Total Reads**
1	IGIB1130482058V	62862874
2	IGIB1130534086V	39077048
3	IGIB113050041V	19037992
4	IGIB1130469734V	88483774
5	IGIB1130519233V	78029270
6	IGIB1130519238V	44719046
7	IGIB1130536690V	19406400
8	IGIB1130429966V	12640314
9	IGIB1130534678V	35228114
10	IGIB1130540315V	26543080
11	IGIB1130532747V	11654962
12	IGIB1130797709V	25562518
13	IGIB1130493051V	11360978
14	IGIB1130538756V	16257504
15	IGIB1130527553V	15073554
16	IGIB11301042671V	90926848
17	IGIB1130111V	46806054
18	IGIB1130359925V	47785046
19	IGIB1130371022V	64849088
20	IGIB113023885V	36187962
21	IGIB113059193V	61832282
22	IGIB1130228653V	18563984
23	IGIB1130341678V	9507746
24	IGIB1130412732V	82006818
25	IGIB1130508933V	33987916
26	IGIB113094002V	27760944
27	IGIB1130222666V	34362574
28	IGIB1130515988V	13467416
29	IGIB11302272	16478916
30	IGIB11303580	33859562
31	IGIB11303722	9100460
32	IGIB11303770	22214872
33	IGIB11303953	35442118
34	IGIB113041961	42473202
35	IGIB113042520	41271994
36	IGIB113052554	16818960
37	IGIB113011563	46907396

38	IGIB11301567	26233712
39	IGIB11301748	64981804
40	IGIB11302237	16565012
41	IGIB11302559	24700988
42	IGIB11303523	728900
43	IGIB11303563	33784726
44	IGIB11303602	5620844
45	IGIB11303711	13895934
46	IGIB113071357	29179770
47	IGIB113071549	2788406
48	IGIB113081916	14431454
49	IGIB11301795	17255966
50	IGIB11301798	1987480
51	IGIB11301829	54372386
52	IGIB11301910	23175322
53	IGIB11301955	21877858
54	IGIB11302264	4235798
55	IGIB11301775	12427994
56	IGIB11303605	27047866

Supplementary table S2: Differentially expressed genes between vaccination breakthrough (VBT) and Unvaccinated (UNV) patients.

Gene ID	log2FoldChange	lfcSE	pvalue	padj	Gene Name (Ensembl)
ENSG00000211459.2	5.960594481	0.718311327	1.06E-16	3.01E-12	MT-RNR1
ENSG00000210082.2	4.525910209	0.663673094	9.14E-12	2.89E-08	MT-RNR2
ENSG00000281383.1	4.390855333	0.637234484	5.56E-12	2.23E-08	FP671120.7
ENSG00000280800.1	4.044606392	0.679461285	2.64E-09	3.14E-06	FP671120.6
ENSG00000225840.2	3.407425876	0.645268246	1.29E-07	4.78E-05	AC010970.1
ENSG00000283907.1	2.491514478	0.630213515	7.70E-05	0.002652729	AD000090.1
ENSG00000256618.3	2.1817098	0.45384596	1.53E-06	0.000242659	MTRNR2L1
ENSG00000286405.1	2.071889085	0.421776302	9.00E-07	0.000175373	AL132799.1
ENSG00000140749.9	-2.00000	0.432146235	3.95E-06	0.000425413	IGSF6
ENSG00000168264.11	-2.00000	0.444311311	6.82E-06	0.000617408	IRF2BP2
ENSG00000185164.15	-2.00000	0.450955087	9.92E-06	0.00076033	NOMO2
ENSG00000168016.15	-2.00000	0.457054463	1.30E-05	0.000889658	TRANK1
ENSG00000136603.14	-2.00000	0.482204458	3.53E-05	0.00163714	SKIL
ENSG00000204525.18	-2.00000	0.549032093	0.000273685	0.00586639	HLA-C
ENSG00000170889.14	-2.001411686	0.465457711	1.71E-05	0.001075407	RPS9
ENSG00000109320.13	-2.002709804	0.463608753	1.56E-05	0.001012386	NFKB1
ENSG00000278705.3	-2.00670691	0.424207957	2.24E-06	0.000297719	H4C2
ENSG00000182541.18	-2.007759941	0.491614664	4.43E-05	0.001882765	LIMK2
ENSG00000122122.10	-2.009920887	0.404144005	6.58E-07	0.000139417	SASH3
ENSG00000165119.21	-2.011228574	0.511126559	8.32E-05	0.002788571	HNRNPK
ENSG00000213719.8	-2.01269537	0.513655302	8.91E-05	0.00289794	CLIC1
ENSG00000126262.5	-2.013244943	0.420291449	1.67E-06	0.000254902	FFAR2
ENSG00000141367.12	-2.013692401	0.507534142	7.26E-05	0.002565263	CLTC
ENSG00000165029.17	-2.013711133	0.484308696	3.21E-05	0.001552387	ABCA1
ENSG00000143933.20	-2.014995831	0.49417754	4.55E-05	0.001903902	CALM2
ENSG00000153802.12	-2.017941194	0.487747578	3.51E-05	0.001635059	TMPRSS11D
ENSG00000198848.13	-2.018098622	0.497387443	4.96E-05	0.001993676	CES1
ENSG00000143207.21	-2.018368742	0.401059351	4.84E-07	0.000107529	COP1
ENSG00000120129.6	-2.019596677	0.574359501	0.000437674	0.007797337	DUSP1
ENSG00000147162.14	-2.021933185	0.465625684	1.41E-05	0.000941018	OGT
ENSG00000042980.13	-2.022122474	0.473120592	1.92E-05	0.001144118	ADAM28
ENSG00000266094.8	-2.022191277	0.434919684	3.33E-06	0.000381786	RASSF5
ENSG00000109814.12	-2.024754672	0.440771774	4.36E-06	0.000452139	UGDH
ENSG00000023330.15	-2.025719964	0.4100108	7.79E-07	0.000155966	ALAS1
ENSG00000110852.5	-2.026228913	0.455446871	8.63E-06	0.000709611	CLEC2B
ENSG00000118515.12	-2.029939931	0.558926411	0.000281391	0.005973043	SGK1
ENSG00000159713.11	-2.031245314	0.468004985	1.42E-05	0.000945957	TPPP3
ENSG00000128016.7	-2.031806925	0.537789742	0.000158049	0.004154845	ZFP36

ENSG00000184678.11	-2.031876126	0.419605095	1.28E-06	0.000219859	H2BC21
ENSG00000107968.10	-2.035435299	0.446033646	5.03E-06	0.0004988	MAP3K8
ENSG00000169228.14	-2.038629537	0.444211399	4.45E-06	0.00045663	RAB24
ENSG00000198668.14	-2.040670116	0.480590713	2.17E-05	0.001257174	CALM1
ENSG00000204389.10	-2.041206801	0.543985897	0.000175209	0.004440214	HSPA1A
ENSG00000198961.10	-2.041438658	0.452886134	6.56E-06	0.000603481	PJA2
ENSG00000221983.8	-2.041685065	0.459441114	8.84E-06	0.000716086	UBA52
ENSG00000145592.14	-2.0421509	0.502927904	4.90E-05	0.001978259	RPL37
ENSG00000166441.13	-2.042847025	0.501126443	4.57E-05	0.001903902	RPL27A
ENSG00000231389.8	-2.044075288	0.476613067	1.80E-05	0.001100871	HLA-DPA1
ENSG00000102144.15	-2.047570162	0.474639237	1.60E-05	0.001031982	PGK1
ENSG00000142089.17	-2.049255574	0.494329556	3.39E-05	0.001615333	IFITM3
ENSG00000139318.8	-2.049411726	0.399498677	2.90E-07	7.71E-05	DUSP6
ENSG00000163191.6	-2.050306972	0.48401125	2.27E-05	0.001291993	S100A11
ENSG00000118680.14	-2.050337304	0.472602355	1.44E-05	0.000951613	MYL12B
ENSG00000123349.15	-2.052005782	0.438745009	2.91E-06	0.000347981	PFDN5
ENSG00000110422.12	-2.052755348	0.458741253	7.65E-06	0.000655398	HIPK3
ENSG00000168298.7	-2.053412058	0.437129955	2.63E-06	0.000331033	H1-4
ENSG00000179869.15	-2.055637669	0.528183673	9.95E-05	0.003099465	ABCA13
ENSG00000115415.20	-2.056256029	0.518469015	7.31E-05	0.00257278	STAT1
ENSG00000275713.3	-2.056282492	0.394204349	1.83E-07	5.90E-05	H2BC9
ENSG00000063046.18	-2.057161469	0.488722729	2.56E-05	0.001377611	EIF4B
ENSG00000270629.7	-2.057558512	0.497008665	3.47E-05	0.00163047	NBPF14
ENSG00000154845.16	-2.062212836	0.465409709	9.38E-06	0.000735105	PPP4R1
ENSG00000173110.8	-2.062982678	0.463572191	8.58E-06	0.000707355	HSPA6
ENSG00000124102.5	-2.064498661	0.46173394	7.78E-06	0.000662809	PI3
ENSG00000174748.22	-2.06489394	0.474351576	1.34E-05	0.00091343	RPL15
ENSG00000137767.14	-2.067575556	0.431267034	1.63E-06	0.000252494	SQOR
ENSG00000100906.11	-2.068074167	0.552236927	0.000180463	0.004510621	NFKBIA
ENSG00000185650.10	-2.069511944	0.440938967	2.69E-06	0.000334592	ZFP36L1
ENSG00000269378.1	-2.072252151	0.398382459	1.98E-07	6.31E-05	ITGB1P1
ENSG00000121966.8	-2.072356522	0.508095394	4.53E-05	0.001903902	CXCR4
ENSG00000131469.15	-2.074114145	0.507687563	4.40E-05	0.001879106	RPL27
ENSG00000103569.10	-2.076037004	0.51673369	5.88E-05	0.002229636	AQP9
ENSG00000156261.13	-2.078301024	0.43914568	2.22E-06	0.00029706	CCT8
ENSG00000153827.14	-2.078652683	0.493075293	2.49E-05	0.001356895	TRIP12
ENSG00000253729.8	-2.079058051	0.461151186	6.53E-06	0.000603194	PRKDC
ENSG00000167996.16	-2.08060142	0.56515883	0.000231915	0.005273895	FTH1
ENSG00000198408.14	-2.083566668	0.4605849	6.08E-06	0.000571721	OGA
ENSG00000260075.1	-2.083572751	0.462714538	6.70E-06	0.000611016	NSFP1
ENSG00000058673.17	-2.083803069	0.504180002	3.58E-05	0.001650282	ZC3H11A
ENSG00000156976.17	-2.084441561	0.49789931	2.83E-05	0.001459324	EIF4A2

ENSG00000105372.8	-2.0887445	0.502706866	3.25E-05	0.001562953	RPS19
ENSG00000257335.8	-2.090118857	0.426483488	9.54E-07	0.000183026	MGAM
ENSG00000090382.7	-2.091317166	0.481282716	1.39E-05	0.000932504	LYZ
ENSG00000168028.14	-2.092149234	0.53983109	0.000106379	0.003232727	RPSA
ENSG00000254911.3	-2.09554151	0.451182617	3.41E-06	0.000385918	SCARNA9
ENSG00000170345.10	-2.095956539	0.566436542	0.000215388	0.00508422	FOS
ENSG00000241343.10	-2.097803562	0.494772795	2.24E-05	0.001279616	RPL36A
ENSG00000116251.11	-2.09823541	0.478145514	1.14E-05	0.000826915	RPL22
ENSG00000181019.13	-2.099808777	0.501578592	2.83E-05	0.001459324	NQO1
ENSG00000180353.11	-2.102544821	0.477247323	1.06E-05	0.000779638	HCLS1
ENSG00000273136.8	-2.115167305	0.413527505	3.14E-07	8.04E-05	NBPF26
ENSG00000169813.17	-2.115733679	0.47918273	1.01E-05	0.00076114	HNRNPF
ENSG00000110321.19	-2.118309127	0.523628283	5.22E-05	0.002054575	EIF4G2
ENSG00000140988.16	-2.119162802	0.499524569	2.21E-05	0.001268407	RPS2
ENSG00000179813.7	-2.121698765	0.467964163	5.79E-06	0.000552718	FAM216B
ENSG00000124942.14	-2.122532103	0.594920576	0.00036005	0.006981093	AHNAK
ENSG00000006125.18	-2.124618737	0.45926216	3.73E-06	0.00040753	AP2B1
ENSG00000120694.20	-2.128748243	0.506262011	2.61E-05	0.001396925	HSPH1
ENSG00000116016.14	-2.128775619	0.523707576	4.81E-05	0.001956089	EPAS1
ENSG00000156875.14	-2.13937713	0.461020511	3.48E-06	0.000389174	MFSD14A
ENSG00000007062.12	-2.141600776	0.468623623	4.88E-06	0.000489641	PROM1
ENSG00000142168.15	-2.142216494	0.484635861	9.86E-06	0.000759646	SOD1
ENSG00000114391.13	-2.142231412	0.515907227	3.29E-05	0.001578413	RPL24
ENSG00000124486.14	-2.143147758	0.469533217	5.01E-06	0.00049814	USP9X
ENSG00000197746.15	-2.145496613	0.546113552	8.54E-05	0.00283194	PSAP
ENSG00000123416.15	-2.147282637	0.493755711	1.37E-05	0.000924509	TUBA1B
ENSG00000122861.16	-2.149323496	0.508909066	2.41E-05	0.001328636	PLAU
ENSG00000165071.15	-2.151880268	0.416807233	2.43E-07	6.96E-05	TMEM71
ENSG00000108654.16	-2.15785521	0.553351987	9.63E-05	0.003041677	DDX5
ENSG00000152601.18	-2.161547153	0.46554216	3.43E-06	0.000385918	MBNL1
ENSG00000213741.11	-2.164497843	0.504850952	1.81E-05	0.001102008	RPS29
ENSG00000137462.9	-2.17217854	0.464442918	2.91E-06	0.000347981	TLR2
ENSG00000163131.12	-2.180599716	0.510378759	1.93E-05	0.001147751	CTSS
ENSG00000156482.11	-2.182037047	0.518084261	2.53E-05	0.001370337	RPL30
ENSG00000089157.16	-2.182145268	0.541690726	5.62E-05	0.002149894	RPLP0
ENSG00000114127.11	-2.189050042	0.491542976	8.45E-06	0.000700826	XRN1
ENSG00000125753.14	-2.190002953	0.435556198	4.95E-07	0.000109248	VASP
ENSG00000104093.14	-2.190795716	0.49962945	1.16E-05	0.000829525	DMXL2
ENSG00000197756.10	-2.191186585	0.532238028	3.84E-05	0.001717163	RPL37A
ENSG00000163349.22	-2.192231081	0.475876661	4.09E-06	0.000432121	HIPK1
ENSG00000111716.14	-2.193088264	0.47006692	3.08E-06	0.000360618	LDHB
ENSG00000248144.6	-2.195342684	0.5586196	8.50E-05	0.00282771	ADH1C

ENSG00000077097.16	-2.195818394	0.429264079	3.13E-07	8.04E-05	TOP2B
ENSG00000152518.8	-2.19896225	0.452192641	1.16E-06	0.000206953	ZFP36L2
ENSG00000058799.15	-2.199951789	0.411356434	8.89E-08	3.77E-05	YIPF1
ENSG00000073921.18	-2.200885935	0.496798585	9.42E-06	0.000735896	PICALM
ENSG00000107854.6	-2.206290362	0.436643522	4.35E-07	9.91E-05	TNKS2
ENSG00000112851.15	-2.206309478	0.495246706	8.39E-06	0.000699879	ERBIN
ENSG00000101265.16	-2.206324	0.464436423	2.03E-06	0.00028527	RASSF2
ENSG00000130741.11	-2.207316149	0.462567834	1.83E-06	0.000266738	EIF2S3
ENSG00000136689.20	-2.208789752	0.548043499	5.57E-05	0.002135269	IL1RN
ENSG00000170759.11	-2.209861122	0.462249448	1.75E-06	0.000260186	KIF5B
ENSG00000070961.16	-2.215231074	0.489289734	5.97E-06	0.000566053	ATP2B1
ENSG00000198189.11	-2.215667517	0.43396317	3.30E-07	8.22E-05	HSD17B11
ENSG00000162244.12	-2.216341616	0.495114636	7.59E-06	0.000654252	RPL29
ENSG00000071082.11	-2.217133459	0.494799707	7.43E-06	0.000649672	RPL31
ENSG00000115760.15	-2.217301604	0.478807489	3.64E-06	0.000401418	BIRC6
ENSG00000070190.13	-2.220454961	0.467035549	1.99E-06	0.000284686	DAPP1
ENSG00000136536.15	-2.221040874	0.459012207	1.31E-06	0.00022152	MARCHF7
ENSG00000163625.16	-2.226719537	0.479249911	3.38E-06	0.000385918	WDFY3
ENSG00000125691.14	-2.227675545	0.495428228	6.91E-06	0.000623896	RPL23
ENSG00000055917.16	-2.233541473	0.467077228	1.74E-06	0.000259888	PUM2
ENSG00000234745.14	-2.233541736	0.585830025	0.000137507	0.003830794	HLA-B
ENSG00000070756.17	-2.23670989	0.497008852	6.78E-06	0.000616537	PABPC1
ENSG00000131171.13	-2.245421739	0.377266157	2.65E-09	3.14E-06	SH3BGRL
ENSG00000135842.17	-2.245895074	0.433195179	2.17E-07	6.64E-05	NIBAN1
ENSG00000167526.14	-2.245975777	0.492471349	5.10E-06	0.000501975	RPL13
ENSG00000157557.13	-2.247535099	0.512921023	1.18E-05	0.000835163	ETS2
ENSG00000120063.10	-2.248435238	0.494962407	5.56E-06	0.000538705	GNA13
ENSG00000101336.18	-2.258735759	0.407051365	2.87E-08	1.67E-05	HCK
ENSG00000024048.11	-2.259068908	0.453536368	6.33E-07	0.000135277	UBR2
ENSG00000169180.12	-2.260990722	0.446158336	4.03E-07	9.47E-05	XPO6
ENSG00000180596.8	-2.264125126	0.431830265	1.58E-07	5.42E-05	H2BC4
ENSG00000109475.17	-2.264204016	0.509303835	8.76E-06	0.000714223	RPL34
ENSG00000115524.17	-2.264879683	0.505711806	7.51E-06	0.000652236	SF3B1
ENSG00000196683.12	-2.265421562	0.447519451	4.15E-07	9.55E-05	TOMM7
ENSG00000171858.18	-2.265735824	0.468721814	1.34E-06	0.000222266	RPS21
ENSG00000280987.4	-2.266839587	0.499916107	5.78E-06	0.000552718	MATR3
ENSG00000125107.19	-2.267351821	0.483484662	2.74E-06	0.000336481	CNOT1
ENSG00000173535.15	-2.268113127	0.38043696	2.49E-09	3.14E-06	TNFRSF10C
ENSG00000196126.12	-2.270457603	0.472516701	1.55E-06	0.000242659	HLA-DRB1
ENSG00000117450.15	-2.271931417	0.572305993	7.19E-05	0.002552553	PRDX1
ENSG00000140575.13	-2.275330866	0.494141013	4.13E-06	0.000432121	IQGAP1
ENSG00000107372.13	-2.276375147	0.494048521	4.07E-06	0.000432121	ZFAND5

ENSG00000100219.17	-2.27657651	0.494034037	4.06E-06	0.000432121	XBP1
ENSG00000129083.13	-2.279646479	0.442067491	2.51E-07	7.01E-05	COPB1
ENSG00000204592.9	-2.284271546	0.547414575	3.01E-05	0.001514483	HLA-E
ENSG00000147065.17	-2.284314699	0.460761051	7.13E-07	0.000147028	MSN
ENSG00000137818.12	-2.285589734	0.481722824	2.09E-06	0.000289855	RPLP1
ENSG00000185201.18	-2.290873928	0.506788757	6.17E-06	0.000573815	IFITM2
ENSG00000132475.10	-2.29541487	0.539796217	2.12E-05	0.001227836	H3-3B
ENSG00000182899.17	-2.296816991	0.50830538	6.23E-06	0.000576817	RPL35A
ENSG00000171863.15	-2.30029863	0.484723669	2.08E-06	0.000289855	RPS7
ENSG00000153201.16	-2.300360842	0.510569411	6.62E-06	0.000607626	RANBP2
ENSG00000136942.15	-2.300711193	0.473020488	1.15E-06	0.000206953	RPL35
ENSG00000270149.5	-2.301174622	0.559197151	3.87E-05	0.001722417	AL591806.2
ENSG00000080824.19	-2.302147093	0.564098227	4.48E-05	0.00189434	HSP90AA1
ENSG00000124237.6	-2.302384182	0.4764828	1.35E-06	0.000222266	C20orf85
ENSG00000081320.11	-2.303121138	0.466764131	8.05E-07	0.000159257	STK17B
ENSG00000065809.14	-2.306599086	0.488515529	2.34E-06	0.000306644	FAM107B
ENSG00000148303.17	-2.310408905	0.515980582	7.54E-06	0.000652307	RPL7A
ENSG00000197329.12	-2.311429879	0.495771553	3.13E-06	0.000364541	PELI1
ENSG00000153922.11	-2.314402559	0.492120152	2.56E-06	0.000327699	CHD1
ENSG00000177954.14	-2.314663108	0.52974158	1.25E-05	0.00086216	RPS27
ENSG00000012660.14	-2.317368394	0.447387956	2.22E-07	6.64E-05	ELOVL5
ENSG00000119535.18	-2.317700726	0.500263586	3.60E-06	0.000400522	CSF3R
ENSG00000124614.16	-2.319933639	0.522723457	9.07E-06	0.00072409	RPS10
ENSG00000142676.14	-2.321219465	0.535415336	1.46E-05	0.000960366	RPL11
ENSG00000137642.13	-2.321586219	0.485201654	1.71E-06	0.000258976	SORL1
ENSG00000129003.19	-2.322876875	0.507108234	4.64E-06	0.000472609	VPS13C
ENSG00000110700.7	-2.323952692	0.499689654	3.31E-06	0.000381786	RPS13
ENSG00000158470.5	-2.329173969	0.521166316	7.85E-06	0.000662809	B4GALT5
ENSG00000149273.15	-2.329676859	0.490018542	1.99E-06	0.000284686	RPS3
ENSG00000252835.1	-2.329842426	0.481129531	1.28E-06	0.000219859	SCARNA21
ENSG00000134419.15	-2.346868854	0.502426156	3.00E-06	0.000356599	RPS15A
ENSG00000057608.17	-2.348673991	0.453382282	2.21E-07	6.64E-05	GDI2
ENSG00000156273.16	-2.355313581	0.496546481	2.10E-06	0.000290199	BACH1
ENSG00000276966.3	-2.359200769	0.441981318	9.41E-08	3.82E-05	H4C5
ENSG00000170315.14	-2.360855958	0.504203145	2.84E-06	0.000344648	UBB
ENSG00000229117.9	-2.361392006	0.547143128	1.59E-05	0.00102543	RPL41
ENSG00000143669.15	-2.363335898	0.477955492	7.63E-07	0.000154959	LYST
ENSG00000116489.13	-2.363518107	0.495341301	1.83E-06	0.000266738	CAPZA1
ENSG00000100129.18	-2.370616621	0.44300822	8.74E-08	3.77E-05	EIF3L
ENSG00000121858.11	-2.37574765	0.507265357	2.82E-06	0.000344377	TNFSF10
ENSG00000163563.8	-2.377123421	0.433555479	4.19E-08	2.20E-05	MNDA
ENSG00000196954.14	-2.378844047	0.473862263	5.16E-07	0.000112126	CASP4

ENSG00000273802.3	-2.380227035	0.383836904	5.61E-10	9.38E-07	H2BC8
ENSG00000167552.15	-2.380835153	0.488104557	1.07E-06	0.000196945	TUBA1A
ENSG00000147416.11	-2.381253889	0.481038875	7.41E-07	0.000151685	ATP6V1B2
ENSG00000086061.16	-2.387508795	0.533119014	7.52E-06	0.000652236	DNAJA1
ENSG00000170458.15	-2.390567331	0.423462235	1.65E-08	1.12E-05	CD14
ENSG00000288640.1	-2.397071627	0.452434324	1.17E-07	4.60E-05	AC005192.1
ENSG00000177600.9	-2.39745578	0.4917397	1.09E-06	0.000197935	RPLP2
ENSG00000133112.17	-2.400177705	0.54360794	1.01E-05	0.00076114	TPT1
ENSG00000197061.5	-2.405503121	0.492594154	1.04E-06	0.000195189	H4C3
ENSG00000109971.14	-2.407628002	0.588195284	4.25E-05	0.00185265	HSPA8
ENSG00000163041.13	-2.408250242	0.487286816	7.73E-07	0.000155859	H3-3A
ENSG00000182774.13	-2.408843151	0.493735348	1.07E-06	0.000196945	RPS17
ENSG00000163421.9	-2.412128517	0.412839621	5.13E-09	5.04E-06	PROK2
ENSG00000124107.5	-2.412302534	0.602056989	6.16E-05	0.002300762	SLPI
ENSG00000126432.14	-2.413008938	0.520588218	3.57E-06	0.000397841	PRDX5
ENSG00000028137.19	-2.41634826	0.460836868	1.58E-07	5.42E-05	TNFRSF1B
ENSG00000107290.14	-2.421148516	0.501660065	1.39E-06	0.000224823	SETX
ENSG00000182782.8	-2.421238693	0.472173804	2.93E-07	7.71E-05	HCAR2
ENSG00000108298.12	-2.422571054	0.511968049	2.22E-06	0.00029706	RPL19
ENSG00000198242.14	-2.423527748	0.529840616	4.78E-06	0.000484197	RPL23A
ENSG00000096384.20	-2.424207451	0.546338479	9.11E-06	0.00072409	HSP90AB1
ENSG00000144713.13	-2.42547952	0.522343619	3.43E-06	0.000385918	RPL32
ENSG00000135373.13	-2.426700327	0.516663775	2.64E-06	0.000331033	EHF
ENSG00000187837.4	-2.429730414	0.436397478	2.58E-08	1.53E-05	H1-2
ENSG00000184588.18	-2.437749281	0.497885189	9.77E-07	0.000185272	PDE4B
ENSG00000255398.3	-2.438530337	0.503295635	1.27E-06	0.000219456	HCAR3
ENSG00000244067.3	-2.43909541	0.495567296	8.57E-07	0.000168194	GSTA2
ENSG00000163220.11	-2.441289376	0.630449084	0.000107812	0.003255424	S100A9
ENSG00000231500.7	-2.44210364	0.556755252	1.15E-05	0.000828363	RPS18
ENSG00000142541.18	-2.449429478	0.54642457	7.37E-06	0.000649672	RPL13A
ENSG00000215301.11	-2.456133979	0.545323598	6.67E-06	0.000609929	DDX3X
ENSG00000067082.15	-2.456211543	0.555948546	9.96E-06	0.00076114	KLF6
ENSG00000197249.14	-2.459198568	0.487538126	4.56E-07	0.000102045	SERPINA1
ENSG00000116679.16	-2.46832778	0.477158043	2.30E-07	6.69E-05	IVNS1ABP
ENSG00000197405.8	-2.469912194	0.459565233	7.68E-08	3.55E-05	C5AR1
ENSG00000265681.8	-2.470210867	0.520063521	2.04E-06	0.00028527	RPL17
ENSG00000205413.8	-2.474773793	0.564283323	1.16E-05	0.000828363	SAMD9
ENSG00000144802.11	-2.476023634	0.519219293	1.85E-06	0.000269026	NFKBIZ
ENSG00000161970.15	-2.476373862	0.522765427	2.17E-06	0.000293699	RPL26
ENSG00000131236.18	-2.47897692	0.506645298	9.93E-07	0.000187144	CAP1
ENSG00000135821.19	-2.4818386	0.547008541	5.70E-06	0.000548028	GLUL
ENSG00000111913.20	-2.484476115	0.470098713	1.26E-07	4.78E-05	RIPOR2

ENSG00000027697.16	-2.487819831	0.46391521	8.20E-08	3.70E-05	IFNGR1
ENSG00000204628.12	-2.490223927	0.488584023	3.45E-07	8.47E-05	RACK1
ENSG00000266412.6	-2.491777834	0.479568063	2.04E-07	6.44E-05	NCOA4
ENSG00000155307.19	-2.50268835	0.491480467	3.54E-07	8.58E-05	SAMSN1
ENSG00000152818.19	-2.507713681	0.516621987	1.21E-06	0.000215036	UTRN
ENSG00000146278.11	-2.514458901	0.444548867	1.55E-08	1.09E-05	PNRC1
ENSG00000238741.1	-2.515791341	0.570967475	1.05E-05	0.000779638	SCARNA7
ENSG00000091317.8	-2.517695192	0.46776933	7.35E-08	3.49E-05	CMTM6
ENSG00000133872.14	-2.518049901	0.475405597	1.18E-07	4.60E-05	SARAF
ENSG00000113368.12	-2.52338881	0.471429351	8.67E-08	3.77E-05	LMNB1
ENSG00000062716.13	-2.524168952	0.551589179	4.74E-06	0.000481062	VMP1
ENSG00000277209.1	-2.526188163	0.62901122	5.92E-05	0.002240777	RPPH1
ENSG00000139644.13	-2.527323601	0.528468897	1.73E-06	0.000259888	TMBIM6
ENSG00000111348.9	-2.528094574	0.492646724	2.87E-07	7.71E-05	ARHGDIB
ENSG00000186468.13	-2.528446504	0.509129699	6.83E-07	0.000142791	RPS23
ENSG00000125772.14	-2.529845055	0.499159414	4.02E-07	9.47E-05	GPCPD1
ENSG00000170525.21	-2.539799943	0.458201142	2.97E-08	1.69E-05	PFKFB3
ENSG00000147604.14	-2.541067547	0.556092825	4.89E-06	0.000489641	RPL7
ENSG00000274266.1	-2.544410529	0.661917864	0.000121046	0.003520492	SNORA73A
ENSG00000152558.15	-2.544722857	0.496287174	2.94E-07	7.71E-05	TMEM123
ENSG00000163682.17	-2.547966051	0.470063172	5.94E-08	2.95E-05	RPL9
ENSG00000156508.19	-2.549786559	0.568915686	7.40E-06	0.000649672	EEF1A1
ENSG00000171049.9	-2.552060355	0.463799107	3.74E-08	2.01E-05	FPR2
ENSG00000089009.16	-2.555904301	0.498575481	2.95E-07	7.71E-05	RPL6
ENSG00000137575.12	-2.557318068	0.538285687	2.03E-06	0.00028527	SDCBP
ENSG00000197958.13	-2.55963606	0.518790987	8.06E-07	0.000159257	RPL12
ENSG00000134294.14	-2.562372106	0.539325792	2.02E-06	0.00028527	SLC38A2
ENSG00000142937.12	-2.568353944	0.542050029	2.16E-06	0.000293423	RPS8
ENSG00000198755.11	-2.570397968	0.516945407	6.62E-07	0.000139417	RPL10A
ENSG00000206503.13	-2.572904386	0.569093037	6.15E-06	0.000573815	HLA-A
ENSG00000142534.7	-2.581006377	0.545866699	2.26E-06	0.000298584	RPS11
ENSG00000059728.11	-2.583984134	0.551953035	2.85E-06	0.000344648	MXD1
ENSG00000026508.21	-2.586730157	0.51876948	6.16E-07	0.000132637	CD44
ENSG00000159128.16	-2.587832677	0.450667587	9.34E-09	7.59E-06	IFNGR2
ENSG00000163162.9	-2.592030821	0.462658181	2.11E-08	1.34E-05	RNF149
ENSG00000136040.9	-2.599468444	0.486790159	9.29E-08	3.82E-05	PLXNC1
ENSG00000101596.17	-2.604203404	0.53147873	9.59E-07	0.000183026	SMCHD1
ENSG00000147251.16	-2.60744426	0.467858715	2.50E-08	1.51E-05	DOCK11
ENSG00000166920.13	-2.615748812	0.541015917	1.33E-06	0.000222266	C15orf48
ENSG00000112306.8	-2.617744793	0.545162138	1.57E-06	0.000244443	RPS12
ENSG00000171988.20	-2.62589642	0.543441574	1.35E-06	0.000222266	JMJD1C
ENSG00000130066.17	-2.640027491	0.600643934	1.11E-05	0.000806681	SAT1

ENSG00000198604.11	-2.644519899	0.486064938	5.31E-08	2.75E-05	BAZ1A
ENSG00000105835.13	-2.649812703	0.559147439	2.15E-06	0.000293423	NAMPT
ENSG00000198918.8	-2.655529823	0.526380851	4.54E-07	0.000102045	RPL39
ENSG00000163660.12	-2.656937143	0.547723646	1.23E-06	0.000216685	CCNL1
ENSG00000162511.8	-2.664930241	0.52652829	4.16E-07	9.55E-05	LAPTM5
ENSG00000205542.11	-2.667215225	0.567236278	2.57E-06	0.000327699	TMSB4X
ENSG00000268104.3	-2.668498705	0.51925306	2.76E-07	7.55E-05	SLC6A14
ENSG00000254772.10	-2.669193444	0.527308712	4.15E-07	9.55E-05	EEF1G
ENSG00000147403.18	-2.670144701	0.511262667	1.76E-07	5.77E-05	RPL10
ENSG00000239002.3	-2.677991198	0.65850338	4.77E-05	0.001945459	SCARNA10
ENSG00000115956.10	-2.685222299	0.569093221	2.38E-06	0.000310118	PLEK
ENSG00000122406.14	-2.692557339	0.519570719	2.19E-07	6.64E-05	RPL5
ENSG00000118181.11	-2.699299069	0.521486834	2.26E-07	6.64E-05	RPS25
ENSG00000099985.4	-2.70582823	0.498463563	5.69E-08	2.89E-05	OSM
ENSG00000143947.15	-2.712014962	0.526565295	2.60E-07	7.18E-05	RPS27A
ENSG00000133639.6	-2.719562359	0.515468388	1.32E-07	4.82E-05	BTG1
ENSG00000198502.6	-2.732602849	0.473411066	7.83E-09	6.75E-06	HLA-DRB5
ENSG00000114942.14	-2.734473841	0.480650875	1.28E-08	9.56E-06	EEF1B2
ENSG00000173334.4	-2.743457421	0.519212163	1.26E-07	4.78E-05	TRIB1
ENSG00000137154.13	-2.75403376	0.540161186	3.42E-07	8.47E-05	RPS6
ENSG00000122026.11	-2.760028157	0.522779329	1.30E-07	4.78E-05	RPL21
ENSG00000254087.8	-2.761258839	0.516966019	9.23E-08	3.82E-05	LYN
ENSG00000155926.14	-2.763458328	0.485294115	1.24E-08	9.52E-06	SLA
ENSG00000108179.14	-2.763884415	0.511483289	6.53E-08	3.15E-05	PPIF
ENSG00000118503.16	-2.764120957	0.589114166	2.71E-06	0.000334592	TNFAIP3
ENSG00000120690.16	-2.767047109	0.528228567	1.62E-07	5.42E-05	ELF1
ENSG00000100316.16	-2.773065785	0.530588756	1.73E-07	5.72E-05	RPL3
ENSG00000234851.4	-2.781091202	0.513281144	6.02E-08	2.95E-05	RPL23AP42
ENSG00000143546.10	-2.783066767	0.588634963	2.27E-06	0.000298584	S100A8
ENSG00000184557.4	-2.783347024	0.468719495	2.88E-09	3.28E-06	SOCS3
ENSG00000165030.4	-2.789200867	0.463088409	1.71E-09	2.43E-06	NFIL3
ENSG00000100201.23	-2.790905784	0.505940985	3.46E-08	1.93E-05	DDX17
ENSG00000233369.7	-2.803663443	0.493557047	1.34E-08	9.79E-06	GTF2IP4
ENSG00000143384.14	-2.84935628	0.54979891	2.19E-07	6.64E-05	MCL1
ENSG00000059804.16	-2.851870643	0.536300483	1.05E-07	4.21E-05	SLC2A3
ENSG00000198034.11	-2.864623501	0.546629175	1.60E-07	5.42E-05	RPS4X
ENSG00000163565.20	-2.86709381	0.509706145	1.86E-08	1.23E-05	IFI16
ENSG00000143226.15	-2.874687351	0.496447062	7.02E-09	6.24E-06	FCGR2A
ENSG00000112303.14	-2.877292116	0.496286075	6.73E-09	6.17E-06	VNN2
ENSG00000143119.14	-2.886420749	0.517418335	2.43E-08	1.50E-05	CD53
ENSG00000198814.14	-2.894424692	0.478918672	1.51E-09	2.26E-06	GK
ENSG00000265972.6	-2.901654354	0.552435665	1.50E-07	5.34E-05	TXNIP

ENSG00000116701.15	-2.909932765	0.509334333	1.11E-08	8.76E-06	NCF2
ENSG00000174444.15	-2.935435546	0.519281358	1.58E-08	1.09E-05	RPL4
ENSG00000116044.16	-2.945278463	0.505418163	5.63E-09	5.34E-06	NFE2L2
ENSG00000145425.10	-2.947976216	0.535214876	3.63E-08	1.98E-05	RPS3A
ENSG00000263639.7	-2.955615591	0.468935789	2.92E-10	5.26E-07	MSMB
ENSG00000123689.6	-2.964026644	0.502277353	3.61E-09	3.67E-06	G0S2
ENSG00000166710.23	-3.005457805	0.625290172	1.54E-06	0.000242659	B2M
ENSG00000116741.8	-3.019411856	0.439299247	6.28E-12	2.23E-08	RGS2
ENSG00000229807.13	-3.055755485	0.582989567	1.59E-07	5.42E-05	XIST
ENSG00000159388.6	-3.068058048	0.514781469	2.52E-09	3.14E-06	BTG2
ENSG00000180871.8	-3.132171455	0.470070811	2.68E-11	7.62E-08	CXCR2
ENSG00000189067.14	-3.221010976	0.532027074	1.41E-09	2.23E-06	LITAF
ENSG00000151726.15	-3.274899984	0.552096466	3.00E-09	3.28E-06	ACSL1
ENSG00000118564.15	-3.326394308	0.525410493	2.44E-10	5.20E-07	FBXL5
ENSG00000073756.13	-3.360626341	0.516524881	7.71E-11	1.83E-07	PTGS2
ENSG00000122862.5	-3.363616101	0.599258982	1.99E-08	1.29E-05	SRGN
ENSG00000169429.12	-3.403649132	0.66610613	3.23E-07	8.19E-05	CXCL8
ENSG00000136167.15	-3.403971976	0.574804864	3.18E-09	3.35E-06	LCP1
ENSG00000174718.12	-3.420096411	0.542804531	2.96E-10	5.26E-07	RESF1
ENSG00000251562.9	-3.475368642	0.646792913	7.73E-08	3.55E-05	MAL
ENSG00000162747.12	-3.478837183	0.473616911	2.05E-13	1.17E-09	FCGR3B
ENSG00000171051.9	-3.500397938	0.553554084	2.56E-10	5.20E-07	FPR1
ENSG00000163464.8	-3.70358113	0.484195311	2.03E-14	1.92E-10	CXCR1
ENSG00000204287.14	-3.805595141	0.578521689	4.76E-11	1.23E-07	HLA-DRA
ENSG00000081237.20	-3.860231261	0.560367448	5.63E-12	2.23E-08	PTPRC
ENSG00000125538.12	-3.93864313	0.5354616	1.90E-13	1.17E-09	IL1B
ENSG00000269867.1	-4.490684567	0.567195187	2.43E-15	3.45E-11	novel transcript, sense intronic to ZNF814

Supplementary table S3: GO enrichment analysis for the differentially expressed genes to identify associated pathways

GO ID	Description	Gene Ratio	Enrichment score	pvalue	p.adjust	qvalue	Gene ID	Count
GO:0022613	ribonucleoprotein complex biogenesis	43/318	0.135220126	0	0	0	RPL38/RPS9/EIF4B/RPL27/PRKDC/RPS19/RPSA/RPS2/RNU1-1/ RPL24/ RMRP/RPLP0/EIF2S3/SF3B1/RPS21/RPL35A/RPS7/RPL35/HSP90AA1/RPL7A/RPS27/RPS10/RPL11/EIF3L/RNVU1-7/RPS17/SETX/RPL23A/HSP90AB1/RPL13A/DDX3X/RPL26/GLUL/RPL7/RPL6/RPL12/RPS8/RPL10A/RPL10/RPL5/RPS6/RPL3/DDX17	43
GO:0051090	regulation of DNA-binding transcription factor activity	19/318	0.059748428	0.000281214	0.006933983	0.00551409	ADGRG3/NFKB1/HSPA1A/UBA52/NFKBIA/FOS/HCLS1/TLR2/ERBIN/HCK/PELI1/RPS3/UBB/S100A9/TMSB4X/RPS27A/TRIB1/S100A8/IL1B	19
GO:0006417	regulation of translation	29/318	0.091194969	7.22783E-10	8.92637E-08	7.09849E-08	GAPDH/RPL38/RPS9/ZFP36/EIF4B/ZFP36L1/PRKDC/EIF4A2/EIF4G2/RPL30/XRN1/ZFP36L2/EIF2S3/PUM2/PABPC1/NIBAN1/MATR3/CNOT1/RPLP1/RPS3/RPL13A/DDX3X/RPL26/RACK1/RPL10/RPL5/RPS4X/RGS2/BTG2	29
GO:0043161	proteasome-mediated ubiquitin-dependent protein catabolic process	17/318	0.05345912	0.000914972	0.016865537	0.013411929	RNF19A/PSME1/COP1/OGT/HSPA1A/UBA52/UBR2/PELI1/RPL11/UBB/HSP90AB1/RACK1/SDCBP/RPS27A/TRIB1/NFE2L2/FBXL5	17
GO:0006402	mRNA catabolic process	76/318	0.238993711	0	0	0	PSME1/RPS20/RPL38/RPS9/ZFP36/HSPA1A/UBA52/RPL37/RPL27A/RPL15/ZFP36L1/RPL27/RPS19/RPSA/RPL36A/RPL22/RPS2/RPL24/DDX5/RPS29/RPL30/RPLP0/XRN1/RPL37A/ZFP36L2/RPL29/RPL31/RPL23/PUM2/PABPC1/RPL13/RPL34/RPS21/CNOT1/RPLP1/RPL35A/RPS7/RPL35/RPL7A/RPS27/RPS10/RPL11/RPS13/RPS3/RPS15A/UBB/RPL41/RPLP2/HSPA8/RPS17/RPL19/RPL23A/RPL32/RPS18/RPL13A/RPL26/RPS23/RPL7/RPL9/RPL6/RPL12/RPS8/RPL10A/RPS11/RPS12/RPL39/RPL10/RPL5/RPS25/RPS27A/RPS6/RPL3/RPS4X/RPL4/RPS3A/BTG2	76
GO:1903311	regulation of mRNA metabolic process	17/318	0.05345912	8.11237E-05	0.002636521	0.002096632	PSME1/HNRNPK/ZFP36/HSPA1A/UBA52/ZFP36L1/DDX5/XRN1/ZFP36L2/PUM2/PABPC1/CNOT1/UBB/HSPA8/RPS27A/DDX17/BTG2	17

GO:0042254	ribosome biogenesis	33/318	0.103773585	0	0	0	RPL38/RPS9/RPL27/PRKDC/RPS19/RPSA/RPS2/RPL24/RMRP/RPLP0/RPS21/RPL35A/RPS7/RPL35/RPL7A/RPS27/RPS10/RPL11/RPS17/RPL23A/DDX3X/RPL26/GLUL/RPL7/RPL6/RPL12/RPS8/RPL10A/RPL10/RPL5/RPS6/RPL3/DDX17	33
GO:0071826	ribonucleoprotein complex subunit organization	26/318	0.081761006	0	0	0	RPL38/EIF4B/PRKDC/RPS19/RPSA/RNU1-1/RPL24/RPLP0/EIF2S3/KIF5B/SF3B1/HSP90AA1/RPS27/RPS10/RPL11/EIF3L/RNVU1-7/SETX/RPL23A/HSP90AB1/RPL13A/RPL6/RPL12/RPL10/RPL5/RPL3	26
GO:0016072	rRNA metabolic process	22/318	0.06918239	1.11418E-10	1.4743E-08	1.1724E-08	RPS9/RPL27/PRKDC/RPS19/RPS2/RMRP/XRN1/RPS21/RPL35A/RPS7/RPL35/RPL7A/RPS27/RPL11/RPS17/RPL26/RPL7/RPS8/RPL10A/RPL5/RPS6/DDX17	22
GO:0022618	ribonucleoprotein complex assembly	25/318	0.078616352	0	0	0	RPL38/EIF4B/PRKDC/RPS19/RPSA/RNU1-1/RPL24/RPLP0/EIF2S3/SF3B1/HSP90AA1/RPS27/RPS10/RPL11/EIF3L/RNVU1-7/SETX/RPL23A/HSP90AB1/RPL13A/RPL6/RPL12/RPL10/RPL5/RPL3	25
GO:0006364	rRNA processing	21/318	0.066037736	2.94294E-10	3.75986E-08	2.98994E-08	RPS9/RPL27/PRKDC/RPS19/RPS2/RMRP/RPS21/RPL35A/RPS7/RPL35/RPL7A/RPS27/RPL11/RPS17/RPL26/RPL7/RPS8/RPL10A/RPL5/RPS6/DDX17	21
GO:0019080	viral gene expression	68/318	0.213836478	0	0	0	RPS20/RPL38/RPS9/ZFP36/UBA52/RPL37/RPL27A/IFITM3/RPL15/RPL27/RPS19/RPSA/RPL36A/RPL22/RPS2/RPL24/RPS29/RPL30/RPLP0/RPL37A/RPL29/RPL31/RPL23/RPL13/RPL34/RPS21/RPLP1/RPL35A/RPS7/RANBP2/RPL35/RPL7A/CHD1/RPS27/RPS10/RPL11/RPS13/RPS3/RPS15A/RPL41/EIF3L/RPLP2/RPS17/RPL19/RPL23A/RPL32/RPS18/RPL13A/RPL26/RPS23/RPL7/RPL9/RPL6/RPL12/RPS8/RPL10A/RPS11/RPS12/RPL39/RPL10/RPL5/RPS25/RPS27A/RPS6/RPL3/RPS4X/RPL4/RPS3A	68

GO:0006413	translational initiation	70/318	0.220125786	0	0	0	RPS20/RPL38/RPS9/UBA52/RPL37/RPL27A/EIF4B/RPL15/RPL27/EIF4A2/RPS19/RPSA/RPL36A/RPL22/EIF4G2/RPS2/RPL24/RPS29/RPL30/RPLP0/RPL37A/EIF2S3/RPL29/RPL31/RPL23/PABPC1/RPL13/RPL34/RPS21/RPLP1/RPL35A/RPS7/RPL35/RPL7A/RPS27/RPS10/RPL11/RPS13/RPS3/RPS15A/RPL41/EIF3L/RPLP2/RPS17/RPL19/RPL23A/RPL32/RPS18/RPL13A/DDX3X/RPL26/RPS23/RPL7/RPL9/RPL6/RPL12/RPS8/RPL10A/RPS11/RPS12/RPL39/RPL10/RPL5/RPS25/RPS27A/RPS6/RPL3/RPS4X/RPL4/RPS3A	70
GO:0043488	regulation of mRNA stability	12/318	0.037735849	7.80396E-05	0.002567689	0.002041895	PSME1/ZFP36/HSPA1A/UBA52/ZFP36L1/XRN1/ZFP36L2/PUM2/PABPC1/UBB/HSPA8/RPS27A	12
GO:0019083	viral transcription	67/318	0.210691824	0	0	0	RPS20/RPL38/RPS9/ZFP36/UBA52/RPL37/RPL27A/IFITM3/RPL15/RPL27/RPS19/RPSA/RPL36A/RPL22/RPS2/RPL24/RPS29/RPL30/RPLP0/RPL37A/RPL29/RPL31/RPL23/RPL13/RPL34/RPS21/RPLP1/RPL35A/RPS7/RANBP2/RPL35/RPL7A/CHD1/RPS27/RPS10/RPL11/RPS13/RPS3/RPS15A/RPL41/RPLP2/RPS17/RPL19/RPL23A/RPL32/RPS18/RPL13A/RPL26/RPS23/RPL7/RPL9/RPL6/RPL12/RPS8/RPL10A/RPS11/RPS12/RPL39/RPL10/RPL5/RPS25/RPS27A/RPS6/RPL3/RPS4X/RPL4/RPS3A	67
GO:0043312	neutrophil degranulation	41/318	0.128930818	0	0	0	ADGRG3/NFKB1/RAB24/HSPA1A/S100A11/ABCA13/HSPA6/CCT8/FTH1/MGAM/LYZ/PLAU/TLR2/CTSS/IQGAP1/COPB1/HSP90AA1/GDI2/MNDA/HSPA8/SLPI/TNFRSF1B/HSP90AB1/S100A9/DDX3X/SERPINA1/C5AR1/CAP1/CMTM6/EEF1A1/FPR2/SDCBP/S100A8/SLC2A3/FCGR2A/CD53/CXCR2/FCGR3B/FPR1/CXCR1/PTPRC	41
GO:0002283	neutrophil activation involved in immune response	41/318	0.128930818	0	0	0	ADGRG3/NFKB1/RAB24/HSPA1A/S100A11/ABCA13/HSPA6/CCT8/FTH1/MGAM/LYZ/PLAU/TLR2/CTSS/IQGAP1/COPB1/HSP90AA1/GDI2/MNDA/HSPA8/SLPI/TNFRSF1B/HSP90AB1/S100A9/DDX3X/SERPINA1/C5AR1/CAP1/CMTM6/EEF1A1/FPR2/SDCBP/S100A8/SLC2A3/FCGR2A/CD53/CX	41

							CR2/FCGR3B/FPR1/CXCR1/PTPRC	
GO:2001233	regulation of apoptotic signaling pathway	20/318	0.062893082	0.00021803	0.005811507	0.004621467	SASH3/MAP3K8/ZFP36L1/PRKDC/HSPH1/SOD1/ZFP36L2/MARCHF7/HLA-E/MSN/PELI1/RPS3/TNFRSF1B/NFKBIZ/RIPOR2/HLA-A/LYN/LCP1/PTPRC/IL1B	20
GO:0002221	pattern recognition receptor signaling pathway	22/318	0.06918239	2.44084E-05	0.001130415	0.000898937	ACTR3/FCGR3A/PSME1/NFKB1/FFAR2/HCK/HSP90AA1/RPS3/MNDA/HSP90AB1/PDE4B/C5AR1/NFKBIZ/FPR2/HLA-A/HLA-DRB5/LYN/ELF1/FCGR2A/FPR1/HLA-DRA/PTPRC	22
GO:0032496	response to lipopolysaccharide	21/318	0.066037736	4.22025E-05	0.001663406	0.001322785	DUSP1/ZFP36/NFKBIA/ZFP36L1/PRKDC/RPS19/MARCHF7/HLA-E/PELI1/MNDA/H4C3/RIPOR2/SAMSN1/TMBIM6/HLA-A/TRIB1/LYN/ELF1/NFE2L2/CXCR2/PTPRC	21
GO:0002224	toll-like receptor signaling pathway	23/318	0.072327044	2.32703E-06	0.000153958	0.000122431	GAPDH/NFKB1/SASH3/FFAR2/HSPA1A/STAT1/PRKDC/SOD1/TLR2/PUM2/HLAE/PELI1/SORL1/RPS3/MNDA/PDE4B/DDX3X/C5AR1/HLAA/OSM/PTGS2/PTPRC/IL1B	23
GO:0001819	positive regulation of cytokine production	21/318	0.066037736	1.19403E-05	0.000631982	0.000502569	NFKB1/FFAR2/NFKBIA/RPS19/SOD1/TLR2/HCK/HLAE/CASP4/TNFRSF1B/S100A9/NFKBIZ/FPR2/TMSB4X/OSM/LYN/S100A8/SOCS3/PTGS2/PTPRC/IL1B	21
GO:0051403	stress-activated MAPK cascade	14/318	0.044025157	0.004995627	0.047767929	0.037986344	GAPDH/PI3/RPS19/LYZ/RPL30/HLAE/SLPI/S100A9/C5AR1/HLAA/RPL39/S100A8/PTPRC/IL1B	14
GO:0034341	response to interferon-gamma	20/318	0.062893082	2.90666E-06	0.000188933	0.000150245	NFKB1/ABCA1/ZFP36/NFKBIA/FOS/TLR2/ERBIN/HCK/PELI1/SLPI/TNFRSF1B/PDE4B/S100A9/C5AR1/TRIB1/LYN/S100A8/LITAF/PTGS2/IL1B	20
GO:0034612	response to tumor necrosis factor	20/318	0.062893082	1.57576E-06	0.000110155	8.7598E-05	DUSP1/MAP3K8/UBA52/DUSP6/HIPK3/SOD1/IQGAP1/SORL1/RPS3/UBB/DNAJA1/PROK2/C5AR1/RPS27A/TRIB1/LYN/RGS2/FPR1/PTPRC/IL1B	20
GO:0050727	regulation of inflammatory response	15/318	0.047169811	0.000545467	0.01141782	0.009079758	SASH3/MAP3K8/HSPH1/SOD1/MARCHF7/HLA-E/PELI1/RPS3/TNFRSF1B/NFKBIZ/RIPOR2/HLA-A/LYN/PTPRC/IL1B	15
GO:0035722	interleukin-12-mediated signaling pathway	18/318	0.056603774	8.99715E-06	0.000505067	0.000401643	TANK/PSME1/NFKB1/ZFP36/HSPA1A/STAT1/NFKBIA/ZFP36L1/HIPK1/ZFP36L2/ERBIN/RPS3/CASP4/TNFRSF1B/RACK1/TMSB4X/NFE2L2/PTGS2	18

GO:0071346	cellular response to interferon-gamma	14/318	0.044025157	0.000831853	0.01581187	0.012574025	HSPA1A/FOS/NQO1/EPAS1/SOD1/PRDX1/RPS3/PRDX5/SETX/RACK1/PPIF/MCL1/NCF2/NFE2L2	14
GO:0002757	immune response-activating signal transduction	17/318	0.05345912	7.7845E-06	0.00045065	0.000358369	NFKB1/DUSP1/ZFP36/MAP3K8/PJA2/UBA52/HIPK3/ZFP36L1/RASSF2/PRDX1/RPS3/UBB/DNAJA1/SDCBP/RPS27A/TRIB1/IL1B	17
GO:1901222	regulation of NIK/NF-kappaB signaling	13/318	0.040880503	0.000571334	0.011825652	0.009404077	MAP3K8/UBA52/DUSP6/SOD1/IQGAP1/RPS3/UBB/PROK2/C5AR1/RPS27A/FPR1/PTPRC/IL1B	13

Supplementary table S4: List of Target genes present in the significant differentially expressed gene set of vaccination breakthroughs for 17 Transcription factors.

**BACH1**	B4GALT5,GDI2,KLF6,NQO1,PRDX1,PSAP,RPS8,SOCS3
**CHD1**	AHNAK,C5AR1,DUSP1,EEF1G,EPAS1,ETS2,FBXL5,FOS,HSP90AB1,KLF6,LITAF,MATR3,MCL1,MXD1,NFIL3,NFKBIA,OSM,PICALM,PLAU,PPIF,PSAP,RPL15,RPL19,RPL22,RPL27A,RPLP0,RPLP2,RPS2,RPS8,S100A11,SCARNA10,SCARNA10,SOCS3,TPT1,TUBA1A,VASP,ZFP36,ZFP36L1
**DDX5**	ABCA1,AP2B1,BAZ1A,BTG2,C5AR1,CAPZA1,CD14,CNOT1,CSF3R,EIF4B,ELF1,EPAS1,FAM107B,FOS,FTH1,HIPK1,HLAE,HNRNPF,HSP90AA1,HSPA1A,IFITM2,IFITM3,IFNGR2,KIF5B,KLF6,LAPTM5,LITAF,LYN,LYZ,MCL1,NQO1,PFDN5,PFKFB3,PLXNC1,PNRC1,PRDX1,PRDX5,PSAP,RPL10A,RPL13A,RPL15,RPL19,RPL23,RPL23A,RPL26,RPL27,RPL27A,RPL29,RPL41,RPL5,RPL6,RPL7A,RPLP0,RPLP1,RPLP2,RPS11,RPS2,RPS21,RPS23,RPS29,RPS3,RPS8,RPSA,SAT1,SETX,SLC2A3,SNORA73A,SOCS3,TMBIM6,TNFRSF10C,TPT1,TUBA1A,UBA52,XBP1,ZC3H11A,ZFP36,ZFP36L2
**EHF**	ACSL1,AHNAK,AP2B1,ARHGDIB,CAP1,CASP4,CCNL1,CLIC1,CLTC,DAPP1,DDX3X,DMXL2,DUSP6,EEF1G,EHF,EIF4A2,EIF4B,ETS2,G0S2,HIPK1,HNRNPF,HSP90AA1,HSPA1A,HSPH1,IFI16,IFITM2,IRF2BP2,IVNS1ABP,LYN,MCL1,MYL12B,NCF2,NFE2L2,NFKB1,PABPC1,PFDN5,PGK1,PLAU,PRDX5,PRKDC,RNF149,RPL11,RPL15,RPL22,RPL23,RPL23A,RPL26,RPL27,RPL27A,RPL30,RPL31,RPL32,RPL34,RPL35,RPL37,RPL4,RPL41,RPL6,RPL7A,RPL9,RPS11,RPS18,RPS19,RPS21,RPS25,RPS3,RPS6,RPS7,RPS8,RPSA,S100A11,SLPI,TMEM123,TNFRSF10C,TNFSF10,TPT1,TRIB1,TRIP12,TUBA1B,TXNIP,UBA52,UBB,USP9X,VASP,VMP1,ZFP36,ZFP36L1
**ELF1**	ARHGDIB,B2M,C5AR1,DUSP1,DUSP6,EEF1G,EIF3L,EIF4A2,EIF4B,ETS2,FOS,FPR1,FPR2,GPCPD1,HNRNPF,HSP90AA1,HSP90AB1,HSPA1A,IFNGR1,IQGAP1,IRF2BP2,LIMK2,LMNB1,MCL1,MYL12B,NFE2L2,OSM,PLXNC1,PPIF,PRDX5,RASSF5,RPL13A,RPL17,RPL19,RPL23,RPL23A,RPL26,RPL27A,RPL3,RPL35,RPL37A,RPL41,RPLP2,RPS11,RPS21,RPS27,RPS3,RPS8,RPS9,S100A11,SCARNA10,SCARNA10,SETX,SKIL,SOCS3,SORL1,TMEM123,TUBA1A,TUBA1B,VASP,VMP1,ZFP36,ZFP36L1,ZFP36L2
**EPAS1**	DUSP1,HNRNPF,HNRNPK,IFNGR2,IRF2BP2,NAMPT,NFIL3,OSM,PABPC1,PFKFB3,PGK1,PICALM,PLXNC1,PNRC1,RGS2,RPL19,RPL3,RPL41,TNFAIP3,TPT1,UBB,ZFP36,ZFP36L1ETS2,AHNAK,ALAS1,B2M,CALM1,CALM2,CAPZA1,CCNL1,CCT8,CLIC1,DDX17,DDX3X,DMXL2,EEF1A1,EIF4A2,EIF4B,ELF1,ETS2,FOS,GDI2,HNRNPF,HSP90AB1,HSPH1,IFNGR1,IRF2BP2,KIF5B,KLF6,NFE2L2,NFKBIZ,PABPC1,PELI1,PFDN5,PRDX5,RNF149,RPL12,RPL13A,RPL15,RPL17,RPL23,RPL23A,RPL24,RPL26,RPL27A,RPL29,RPL35A,RPL41,RPL5,RPL7A,RPLP0,RPS11,RPS15A,RPS18,RPS19,RPS25,RPS27,RPS27A,RPS3,RPS7,RPS8,RPS9,RPSA,SARAF,SAT1,SCARNA10,SCARNA10,SNORA73A,STAT1,TNFSF10,TPT1,TUBA1B,UBR2,UGDH,VAP,VMP1,ZC3H11A,ZFP36,ZFP36L1,ZFP36L2
**ETS2**	AHNAK,ALAS1,B2M,CALM1,CALM2,CAPZA1,CCNL1,CCT8,CLIC1,DDX17,DDX3X,DMXL2,EEF1A1,EIF4A2,EIF4B,ELF1,ETS2,FOS,GDI2,HNRNPF,HSP90AB1,HSPH1,IFNGR1,IRF2BP2,KIF5B,KLF6,NFE2L2,NFKBIZ,PABPC1,PELI1,PFDN5,PRDX5,RNF149,RPL12,RPL13A,RPL15,RPL17,RPL23,RPL23A,RPL24,RPL26,RPL27A,RPL29,RPL35A,RPL41,RPL5,RPL7A,RPLP0,RPS11,RPS15A,RPS18,RPS19,RPS25,RPS27,RPS27A,RPS3,RPS7,RPS8,RPS9,RPSA,SARAF,SAT1,SCARNA10,SCARNA10,SNORA73A,STAT1,TNFSF10,TPT1,TUBA1B,UBR2,UGDH,VASP,VMP1,ZC3H11A,ZFP36,ZFP36L1,ZFP36L2
**FOS**	ABCA1,AHNAK,AP2B1,ARHGDIB,ATP2B1,B2M,B4GALT5,BACH1,BTG1,C15orf48,CALM1,CALM2,CCT8,CD44,CD53,CLIC1,COPB1,DDX3X,DUSP1,EEF1G,EIF3L,EIF4B,ELF1,EPAS1,ETS2,FFAR2,HCAR3,HIPK1,HLA-C,HLA-E , HNRNPF, HSP90AB1, HSPA1A, HSPA8, IFI16, IQGAP1, IRF2BP2, IVNS1ABP, JMJD1C, KLF6,LAPTM5,MCL1,MT-RNR1, MTRNR2, MXD1, MYL12B, NCF2, NFKB1, NQO1, OGT, PABPC1, PDE4B, PFDN5,PJA2,PRDX5,PRKDC,PSAP,PTGS2,RAB24,RANBP2,RASSF5,RNF149,RPL15,RPL24,RPL3,RPL35A, RPL37,RPL41,RPL6,RPLP0,RPLP1,RPLP2,RPS15A,RPS18,RPS25,RPS29,RPS8,S100A11,SARAF,SCARNA10,SCARNA10,SDCBP,SGK1,SLC2A3,SLC38A2,SMCHD1,SNORA73A,SOCS3,SRGN,STAT1,TMBIM6,TMSB4X,TNFAIP3,TNFRSF10C,TOP2B,TRANK1,TRIB1,TUBA1B,UBB,UTRN,VASP,YIPF1,ZC3H11A,ZFAND5,ZFP36,ZFP36L2

**KLF6**	ACSL1,AHNAK,AP2B1,ATP2B1,ATP6V1B2,B2M,B4GALT5,BACH1,BAZ1A,BIRC6,C15orf48,C5AR1,CALM1,CALM2,CAP1,CAPZA1,CASP4,CCNL1,CD14,CD44,CHD1,CLIC1,CLTC,CMTM6,CNOT1,COPB1,DAPP1,DDX17,DDX3X,DDX5,DMXL2,DNAJA1,DUSP1,DUSP6,EEF1A1,EEF1B2,EEF1G,EHF,EIF2S3,EIF4A2,EIF4B,EIF4G2,ELF1,ELOVL5,ETS2,FAM107B,FBXL5,FOS,FTH1,GDI2,GK,GLUL,GNA13,HIPK1,HIPK3,HLA-A,HLA-B,HLA-C,HLA-E, HNRNPF, HNRNPK, HSD17B11, HSP90AA1, HSP90AB1, HSPA1A, HSPA6, HSPA8,IFI16,IFITM2,IFNGR1,IL1B,IQGAP1,IRF2BP2,IVNS1ABP,JMJD1C,KIF5B,KLF6,LDHB,LIMK2,LITAF,LMNB1,LYST,LYZ,MAP3K8,MATR3,MBNL1,MCL1,MSN,MT-RNR1,MT-RNR2, MXD1, MYL12B, NAMPT,NCF2,NFE2L2,NFKB1,NFKBIA,NFKBIZ,NQO1,OGT,OSM,PFKFB3,PGK1,PICALM,PJA2,PLAU,PLXNC1,PNRC1,PPIF,PRDX1,PRDX5,PRKDC,PSAP,PTGS2,RAB24,RANBP2,RPL10,RPL10A,RPL11,RPL12,RPL13,RPL13A,RPL15,RPL19,RPL21,RPL22,RPL23,RPL23A,RPL24,RPL26,RPL27,RPL27A,RPL29,RPL3,RPL30,RPL31,RPL32,RPL35,RPL35A,RPL37,RPL37A,RPL39,RPL4,RPL41,RPL5,RPL6,RPL7,RPL7A,RPL9,RPLP0,RPLP1,RPLP2,RPPH1,RPS11,RPS13,RPS18,RPS19,RPS2,RPS21,RPS23,RPS25,RPS27,RPS27A,RPS29,RPS3,RPS3A,RPS6,RPS7,RPS8,RPS9,RPSA,S100A11,SARAF,SDCBP,SERPINA1,SETX,SF3B1,SGK1,SH3BGRL,SKIL,SLC38A2,SLPI,SNORA73A,SOD1,STAT1,TMBIM6,TMEM123,TMEM71,TMSB4X,TNFRSF10C,TNFSF10,TPT1,TRIB1,TRIP12,TUBA1A,TUBA1B,TXNIP,UBA52,UBB,UBR2,UGDH,USP9X,UTRN,VASP,VMP1,VPS13C,WDFY3,XBP1,XPO6,XRN1,YIPF1,ZC3H11A,ZFAND5,ZFP36,ZFP36L1,ZFP36L2
**LMNB1**	ABCA1,ACSL1,AHNAK,ALAS1,AP2B1,ATP2B1,ATP6V1B2,B2M,B4GALT5,BTG1,BTG2,C15orf48,C5AR1,CALM1,CAP1,CAPZA1,CCNL1,CCT8,CD14,CD44,CLIC1,CLTC,CMTM6,CNOT1,CXCR4,DAPP1,DDX17,DDX3X,DDX5,DNAJA1,DUSP1,DUSP6,EEF1A1,EEF1B2,EEF1G,EIF3L,EIF4B,EIF4G2,ELOVL5,EPAS1,FBXL5,FOS,FPR1,FPR2,FTH1,G0S2,GNA13,GPCPD1,HCAR2,HCAR3,HIPK1,HIPK3,HLA-E,HNRNPF,HSD17B11,HSP90AA1,HSP90AB1,HSPA1A,HSPA8,HSPH1,IFITM2,IFITM3,IFNGR2,IL1RN,IQGAP1,IRF2BP2,JMJD1C,KLF6,LAPTM5,LCP1,LIMK2,LITAF,LMNB1,MAP3K8,MATR3,MBNL1,MCL1,MSN,MXD1,NCF2,NFE2L2,NFKBIA,NFKBIZ,NQO1,OSM,PFDN5,PFKFB3,PICALM,PJA2,PLAU,PLXNC1,PRDX1,PRDX5,PRKDC,PSAP,PUM2,RAB24,RASSF2,RASSF5,RNF149,RPL10,RPL10A,RPL12,RPL13,RPL13A,RPL15,RPL17,RPL19,RPL23,RPL23A,RPL24,RPL26,RPL27,RPL27A,RPL29,RPL30,RPL32,RPL34,RPL35,RPL36A,RPL4,RPL41,RPL5,RPL7A,RPLP0,RPLP1,RPLP2,RPS10,RPS11,RPS12,RPS15A,RPS18,RPS19,RPS2,RPS21,RPS23,RPS25,RPS27,RPS27A,RPS29,RPS3,RPS3A,RPS7,RPS8,RPS9,RPSA,S100A11,SARAF,SASH3,SAT1,SCARNA10,SCARNA10,SCARNA21,SCARNA21,SCARNA9,SETX,SF3B1,SGK1,SLC2A3,SNORA73A,SOCS3,SORL1,TMBIM6,TMEM123,TMSB4X,TNFRSF10C,TNFRSF1B,TOMM7,TPPP3,TPT1,TRIB1,TUBA1A,TUBA1B,UBA52,UBB,USP9X,UTRN,VASP,VMP1,XBP1,ZC3H11A,ZFP36,ZFP36L1,ZFP36L2
**NFE2L2**	CLTC,CXCL8,MAP3K8,MSN,MT-RNR1,MT-RNR2,NQO1,PRDX1,RASSF2,RPS13,RPS8,SOD1
**NFIL3**	AHNAK,CD14,CES1,CSF3R,DDX5,EPAS1,HCAR3,IRF2BP2,JMJD1C,LMNB1,MCL1,MT-RNR1,MT-RNR2,MXD1,NCOA4,NFE2L2,NFIL3,NFKBIA,PGK1,PLXNC1,RPL23,RPL31,RPPH1,SERPINA1,SGK1,SORL1,TMEM123,TNFRSF10C,TRIB1,UBB,UTRN,VMP1,XBP1,ZC3H11A
**NFKB1**	ABCA1,ACSL1,ADAM28,AHNAK,ALAS1,AP2B1,ATP2B1,ATP6V1B2,B2M,B4GALT5,BACH1,BAZ1A,BIRC6,BTG1,BTG2,C15orf48,C5AR1,CALM1,CALM2,CAP1,CAPZA1,CASP4,CCNL1,CCT8,CD14,CD44,CHD1,CLIC1,CLTC,CMTM6,CNOT1,COPB1,CSF3R,CTSS,CXCL8,CXCR4,DAPP1,DDX17,DDX3X,DDX5,DMXL2,DNAJA1,DOCK11,DUSP1,DUSP6,EEF1A1,EEF1B2,EEF1G,EIF2S3,EIF3L,EIF4A2,EIF4B,EIF4G2,ELF1,ELOVL5,EPAS1,ETS2,FAM107B,FBXL5,FCGR2A,FFAR2,FOS,FTH1,G0S2,GDI2,GK,GLUL,GNA13,GPCPD1,HIPK1,HIPK3,HLA-A,HLA-B,HLA-C,HLA-E , HNRNPF, HNRNPK, HSD17B11, HSP90AA1, HSP90AB1, HSPA1A,HSPA6,HSPA8,HSPH1,IFI16,IFITM2,IFNGR1,IFNGR2,IL1RN,IQGAP1,IRF2BP2,IVNS1ABP,JMJD1C,KIF5B,KLF6,LAPTM5,LCP1,LDHB,LIMK2,LITAF,LMNB1,LYN,LYST,MAP3K8,MATR3,MBNL1,MCL1,MSN,MXD1,MYL12B,NAMPT,NBPF14,NBPF14,NCF2,NFE2L2,NFIL3,NFKB1,NFKBIA,NFKBIZ,NQO1,OGT,OSM,PABPC1,PDE4B,PELI1,PFDN5,PFKFB3,PGK1,PICALM,PJA2,PLAU,PLEK,PLXNC1,PNRC1,PPIF,PPP4R1,PRDX1,PRDX5,PRKDC,PROM1,PSAP,PTGS2,PUM2,RAB24,RANBP2,RASSF2,RASSF5,RGS2,RNF149,RPL10,RPL10A,RPL12,RPL13,RPL13A,RPL15,RPL17,RPL19,RPL21,RPL22,RPL23,RPL23A,RPL24,RPL26,RPL27,RPL27A,RPL29,RPL3,RPL30,RPL31,RPL32,RPL34,RPL35,RPL35A,RPL37,RPL37A,RPL39,RPL4,RPL41,RPL5,RPL6,RPL7A,RPL9,RPLP0,RPLP1,RPLP2,RPPH1,RPS10,RPS11,RPS12,RPS13,RPS15A,RPS17,RPS18,RPS19,RPS2,RPS21,RPS23,RPS25,RPS27,RPS27A,RPS29,RPS3,RPS3A,RPS4X,RPS6,RPS7,RPS8,RPS9,S100A11,SARAF,SASH3,SAT1,SCARNA10,SCARNA10,SCARNA21,SCARNA21,SDCBP,SERPINA1,SETX,SF3B1,SGK1,SH3BGRL,SKIL,SLA,SLC2A3,SLC38A2,SMCHD1,SNORA73A,SOCS3,SOD1,SORL1,SRGN,STAT1,STK17B,TMBIM6,TMEM123,TMSB4X,TNFAIP3,TNFRSF10C,TNFRSF1B,TNFSF10,TNKS2,TOMM7,TOP2B,TPPP3,TPT1,TRIB1,TRIP12,TUBA1A,TUBA1B,TXNIP,UBA52,UBB,UBR2,UGDH,USP9X,UTRN,VASP,VMP1,VPS13C,WDFY3,XBP1,XPO6,XRN1,YIPF1,ZC3H11A,ZFAND5,ZFP36,ZFP36L1,ZFP36L2

**OGT**	AP2B1,CALM2,CCT8,CLTC,CNOT1,COPB1,CSF3R,EIF3L,HIPK1,HSP90AB1,HSPH1,MCL1,NCOA4,OGT,PELI1,PFDN5,PPIF,PRDX1,PRDX5,PRKDC,PUM2,RANBP2,RPL11,RPL12,RPL13A,RPL15,RPL19,RPL22,RPL23,RPL23A,RPL24,RPL26,RPL27,RPL27A,RPL29,RPL31,RPL32,RPL34,RPL36A,RPL37,RPL37A,RPL39,RPL4,RPL41,RPL5,RPL6,RPL7,RPL7A,RPL9,RPLP0,RPLP1,RPLP2,RPS11,RPS12,RPS13,RPS15A,RPS18,RPS19,RPS2,RPS21,RPS25,RPS27,RPS27A,RPS3,RPS3A,RPS4X,RPS6,RPS7,RPS8,SNORA73A,TMBIM6,UBA52,UBB,UBR2,VASP,VMP1,XIST,XPO6,XRN1,ZC3H11A,ZFP36
**PRKDC**	ALAS1,LITAF
**STAT1**	ACSL1,AHNAK,ATP2B1,ATP6V1B2,B2M,BAZ1A,BIRC6,BTG2,CALM1,CALM2,CAP1,CAPZA1,CCNL1,CD14,CHD1,CLEC2B,CLTC,COPB1,CXCL8,DAPP1,DDX5,DUSP1,DUSP6,EEF1A1,EEF1G,EIF4A2,ELF1,ELOVL5,EPAS1,ETS2,FBXL5,FCGR2A,FOS,FPR1,FPR2,FTH1,GDI2,GNA13,GPCPD1,HIPK1,HLA-A,HLA-E,HNRNPF,HNRNPK,HSD17B11,HSP90AA1,HSP90AB1,HSPA6,HSPA8,IFI16,IFITM2,IFITM3,IFNGR1,IFNGR2,IQGAP1,IRF2BP2,IVNS1ABP,KLF6,LITAF,LMNB1,LYN,MAP3K8,MATR3,MBNL1,MCL1,MXD1,MYL12B,NAMPT,NFE2L2,NFIL3,NFKB1,NFKBIA,NFKBIZ,OGT,OSM,PABPC1,PELI1,PFKFB3,PGK1,PICALM,PJA2,PLAU,PNRC1,PPP4R1,PRDX1,PRDX5,PSAP,PUM2,RANBP2,RASSF5,RNF149,RPL10A,RPL13,RPL13A,RPL15,RPL17,RPL22,RPL23A,RPL24,RPL26,RPL27,RPL27A,RPL29,RPL3,RPL30,RPL31,RPL35,RPL37,RPL37A,RPL5,RPL7,RPL7A,RPLP1,RPLP2,RPPH1,RPS15A,RPS18,RPS19,RPS2,RPS21,RPS25,RPS27,RPS27A,RPS29,RPS3,RPS3A,RPS7,RPS8,RPS9,RPSA,S100A11,SAMD9,SAT1,SETX,SKIL,SLA,SLC2A3,SLC38A2,SMCHD1,SNORA73A,SOCS3,SORL1,STAT1,STK17B,TMEM123,TMSB4X,TNFRSF10C,TNFRSF10C,TNFSF10,TOP2B,TPT1,TRANK1,TRIB1,TRIP12,TUBA1A,TUBA1B,TXNIP,UBR2,UGDH,VASP,VMP1,WDFY3,XBP1,XRN1,ZFAND5,ZFP36,ZFP36L1,ZFP36L2
**XBP1**	HSP90AB1,PRDX1,RNF149,RPL15,RPL23,RPLP2,RPS8,RPS9,TMEM123,TMSB4X

Supplementary table S5: Pathways identified for the Transcription factors and their corresponding associated genes.

**Common TFs**		
**ELF1**		
R-HSA-8878171	Transcriptional regulation by RUNX1	ABL1,ACTL6A,ACTL6B,AGO1,AGO2,AGO3,AGO4,ARID1A,ARID1B,ARID2,ASH2L,AUTS2,AXIN1,BLK,BMI1,CBFB,CBX2,CBX4,CBX6,CBX8,CCND1,CCND2,CCND3,CCNH,CDK6,CDK7,CLDN5,CR1,CREBBP,CSF2,CSF2,CSNK2A1,CSNK2A2,CSNK2B,CTLA4,CTSK,CTSL,CTSV,DPY30,ELF1,ELF2,EP300,ESR1,FOXP3,GATA1,GATA2,GATA3,GP1BA,GPAM,H19,H2AB1,H2AC14,H2AC18,H2AC20,H2AC4,H2AC6,H2AC7,H2AFX,H2AJ,H2AZ1,H2AZ2,H2BC1,H2BC11,H2BC12,H2BC12L,H2BC13,H2BC14,H2BC15
R-HSA-74160	Gene expression (Transcription)	18S rRNA, 5.8S rRNA, 28S rRNA,28S rRNA,45S pre-rRNA gene,5.8S rRNA,5SrRNA,AAAS,ABCA6,ABL1,ACTB,ACTL6A,ACTL6B,AEBP2,AFF4,AGO1,AGO2,AGO3,AGO4,AGRP,AIFM2,AKT1,AKT2,AKT3,ALYREF,ANAPC1,ANAPC10,ANAPC11,ANAPC15,ANAPC16,ANAPC2,ANAPC4,ANAPC5,ANAPC7,ANG,APAF1,APOE,AR,ARID1A,ARID1B,ARID2,ARID3A,ARID4B,ASH2L,ASUN,ASZ1,ATAD2,ATF2,ATM,ATP1B4,ATR,ATRIP,ATXN3,AURKA,AURKB,AUTS2,AXIN1,BANP,BARD1,BAX,BAZ1,BAZ2A,BBC3,BCDIN3D,BCL2L11,BCL2L14,BCL6,BDNF
R-HSA-212436	Generic Transcription Pathway	ABCA6,ABL1,ACTL6A,ACTL6B,AGO1,AGO2,AGO3,AGO4,AGRP,AIFM2,AKT1,AKT2,AKT3,ANAPC1,ANAPC10,ANAPC11,ANAPC15,ANAPC16,ANAPC2,ANAPC4,ANAPC5,ANAPC7,APAF1,APOE,AR,ARID1A,ARID1B,ARID2,ARID3A,ASH2L,ATAD2,ATF2,ATM,ATP1B4,ATR,ATRIP,ATXN3,AURKA,AURKB,AUTS2,AXIN1,BANP,BARD1,BAX,BBC3,BCL2L11,BCL2L14,BCL6,BDNF,BGLAP,BID,BIRC5,BLK,BLM,BMI1,BMP2,BNIP3L,BRCA1,BRD1,BRD2,BRD7,BRIP1,BRPF1,BRPF3,BTG1,BTG2,CALM1,CAMK2A
R-HSA-8939245	RUNX1 regulates transcription of genes involved in BCR signaling	BLK,CBFB,ELF1,ELF2,PAX5,RUNX1
R-HSA-73857	RNA Polymerase II Transcription	ABCA6,ABL1,ACTL6A,ACTL6B,AFF4,AGO1,AGO2,AGO3,AGO4,AGRP,AIFM2,AKT1,AKT2,AKT3,ALYREF,ANAPC1,ANAPC10,ANAPC11,ANAPC15,ANAPC16,ANAPC2,ANAPC4,ANAPC5,ANAPC7,APAF1,APOE,AR,ARID1A,ARID1B,ARID2,ARID3A,ASH2L,ASUN,ATAD2,ATF2,ATM,ATP1B4,ATR,ATRIP,ATXN3,AURKA,AURKB,AUTS2,AXIN1,BANP,BARD1,BAX,BBC3,BCL2L11,BCL2L14,BCL6,BDNF,BGLAP,BID,BIRC5,BLK,BLM,BMI1,BMP2,BNIP3L,BRCA1,BRD1,BRD2,BRD7,BRIP1,BRPF1,BRPF3,BTG1
R-HSA-8939247	RUNX1 regulates transcription of genes involved in interleukin signaling	CBFB,ELF1,IL3,LIFR,RUNX1
**NFIL3**		
R-HSA-400253	Circadian Clock	ARNTL,ARNTL2,ATF2,AVP,BHLHE40,BHLHE41,BTRC,CARM1,CCRN4L,CHD9,CLOCK,CPT1A,CREB1,CREBBP,CREM,CRTC1,CRTC2,CRTC3,CRY1,CRY2,CSNK1D,CSNK1E,CUL1,DBP,ELOVL3,EP300,F7,FBXL3,HDAC3,HELZ2,HIF1A,KLF15,MED1,MEF2C,MEF2D,NAMPT,NCOA1,NCOA2,NCOA6,NCOR1,NFIL3,NOCT,NPAS2,NR1D1,NR3C1,NRIP1,PER1,PER2,PPARA,PPARGC1A,PPP1CA,PPP1CB,PPP1CC,RAI1,RBM4,RORA,RPS27A,RXRA,SERPINE1,SIK1,SIRT1,SKP1,SMARCD3,SREBF1,TBL1X,TBL1XR1,TGS1,UBA52
**CHD1**		
R-HSA-9006931	Signaling by Nuclear Receptors	ABCA1,ABCG1,ABCG5,ABCG8,ADH1A,ADH1C,ADH4,AGO1,AGO2,AGO3,AGO4,AKR1C3,AKT1,AKT2,AKT3,ALDH1A1,ALDH1A2,ALDH1A3,ALDH8A1,ANGPTL3,APOC1,APOC2,APOC4,APOD,APOE,AREG,ARL4C,ATF2,AXIN1,BCL2,BTC,CALM1,CARM1,CAV1,CAV2,CBFB,CCND1,CCNT1,CDK9,CDKN1B,CETP,CHD1,CITED1,CPT1A,CPT1B,CRABP1,CRABP2,CREB1,CREBBP,CTSD,CXCL12,CXXC5,CYP26A1,CYP26B1,CYP26C1,DDX5,DHRS3,DHRS4,DHRS9,DLAT,DLD,EBAG9,EEPD1,EGF,EGFR,ELK1,EP300,EPGN
R-HSA-9018519	Estrogen-dependent gene expression	AGO1,AGO2,AGO3,AGO4,ATF2,AXIN1,BCL2,CARM1,CBFB,CCND1,CCNT1,CDK9,CHD1,CITED1,CREBBP,CTSD,CXCL12,CXXC5,DDX5,EBAG9,EP300,ERBB4,ESR1,FKBP4,FOS,FOSB,FOXA1,GATA3,GPAM,GREB1,GTF2A1,GTF2A2,GTF2F1,GTF2F2,H2AB1,H2AC14,H2AC18,H2AC20,H2AC4,H2AC6,H2AC7,H2AFX,H2AJ,H2AZ1,H2AZ2,H2BC1,H2BC11,H2BC12,H2BC12L,H2BC13,H2BC14,H2BC15,H2BC17,H2BC21,H2BC3,H2BC4,H2BC5,H2BC9,H2BU1,H3-3A,H3C1,H3C15,H4C1,HDAC1,HSP90AA1,HSP90AB1,JUN,JUND
R-HSA-8939211	ESR-mediated signaling	AGO1,AGO2,AGO3,AGO4,AKT1,AKT2,AKT3,AREG,ATF2,AXIN1,BCL2,BTC,CALM1,CARM1,CAV1,CAV2,CBFB,CCND1,CCNT1,CDK9,CDKN1B,CHD1,CITED1,CREB1,CREBBP,CTSD,CXCL12,CXXC5,DDX5,EBAG9,EGF,EGFR,ELK1,EP300,EPGN,ERBB4,EREG,ESR1,ESR2,FKBP4,FKBP5,FOS,FOSB,FOXA1,FOXO3,GATA3,GNAI1,GNAI2,GNAI3,GNA

		T3,GNB1,GNB2,GNB3,GNB4,GNB5,GNG10,GNG11,GNG12,GNG13,GNG2,GNG3,GNG4,GNG5,GNG7,GNG8,GNGT1,GNGT2,GPAM
R-HSA-162582	Signal Transduction	AAAS,AAMP,AATF,ABCA1,ABCD3,ABCG1,ABCG5,ABCG8,ABHD12,ABHD17A,ABHD17B,ABHD17C,ABHD6,ABI1,ABI2,ABL1,ABL2,ABR,ACBD5,ACKR1,ACKR2,ACKR3,ACKR4,ACTA2,ACTB,ACTC1,ACTG1,ACTN1,ACTN2,ACTR2,ACTR3,ACVR1B,ACVR1C,ACVR2A,ACVR2B,ACVRL1,ADAM10,ADAM12,ADAM17,ADAP1,ADCY1,ADCY10,ADCY2,ADCY3,ADCY4,ADCY5,ADCY6,ADCY7,ADCY8,ADCY9,ADCYAP1,ADCYAP1R1,ADD3,ADGRE1,ADGRE2,ADGRE3,ADGRE5,ADH1A,ADH1C,ADH4,ADM,ADM2,ADORA1,ADORA2A,ADORA2B,ADORA3,ADRA1A,ADRA1B
**LMNB1**		
R-HSA-162582	Signal Transduction	AAAS,AAMP,AATF,ABCA1,ABCD3,ABCG1,ABCG5,ABCG8,ABHD12,ABHD17A,ABHD17B,ABHD17C,ABHD6,ABI1,ABI2,ABL1,ABL2,ABR,ACBD5,ACKR1,ACKR2,ACKR3,ACKR4,ACTA2,ACTB,ACTC1,ACTG1,ACTN1,ACTN2,ACTR2,ACTR3,ACVR1B,ACVR1C,ACVR2A,ACVR2B,ACVRL1,ADAM10,ADAM12,ADAM17,ADAP1,ADCY1,ADCY10,ADCY2,ADCY3,ADCY4,ADCY5,ADCY6,ADCY7,ADCY8,ADCY9,ADCYAP1,ADCYAP1R1,ADD3,ADGRE1,ADGRE2,ADGRE3,ADGRE5,ADH1A,ADH1C,ADH4,ADM,ADM2,ADORA1,ADORA2A,ADORA2B,ADORA3,ADRA1A,ADRA1B
R-HSA-352238	Breakdown of the nuclear lamina	CASP6,LMNA,LMNB1,
R-HSA-111465	Apoptotic cleavage of cellular proteins	ACIN1,ADD1,APC,BCAP31,BIRC2,BMX,CASP3,CASP6,CASP7,CASP8,CDH1,CLSPN,CTNNB1,DBNL,DSG1,DSG2,DSG3,DSP,FNTA,GAS2,GSN,LMNA,LMNB1,MAPT,OCLN,PKP1,PLEC,PRKCD,PRKCQ,PTK2,ROCK1,SATB1,SPTAN1,STK24,STK26,TJP1,TJP2,VIM
R-HSA-9013405	RHOD GTPase cycle	ACTN1,ADD3,AKAP12,ANKFY1,ARHGAP1,ARHGAP12,ARHGAP17,ARHGAP21,ARHGAP26,ARHGAP32,ARHGAP35,ARHGAP39,ARHGAP5,CAPZB,CAV1,CPNE8,DBN1,DEPDC1B,DIAPH1,DIAPH2,DIAPH3,EFHD2,EMD,ESYT1,FILIP1,GOLGA8R,HINT2,LBR,LEMD3,LMAN1,LMNB1,MCAM,MOSPD2,PAK5,PAK6,PGRMC2,PIK3R1,PIK3R2,PLXNA1,PLXNB1,RAB7A,RACGAP1,RHOD,SLC4A7,STBD1,STEAP3,TMPO,TOR1AIP1,VAMP3,VANGL1,VAPB,VRK2,WHAMM,
R-HSA-75153	Apoptotic execution phase	ACIN1,ADD1,APC,BCAP31,BIRC2,BMX,CASP3,CASP6,CASP7,CASP8,CDH1,CLSPN,CTNNB1,DBNL,DFFA,DFFB,DNM1L,DSG1,DSG2,DSG3,DSP,FNTA,GAS2,GSN,H1-0,H1-1,H1-2,H1-3,H1-4,H1-5,HMGB1,HMGB2,KPNA1,KPNB1,LMNA,LMNB1,MAPT,OCLN,PAK2,PKP1,PLEC,PRKCD,PRKCQ,PTK2,ROCK1,SATB1,SPTAN1,STK24,STK26,TJP1,TJP2,VIM,,
R-HSA-1643685	Disease	18S rRNA,1a,28S rRNA,3a,5.8S rRNA,5S rRNA,6,7SL RNA (ENSG00000222619),7SL RNA (ENSG00000222639),7a,8,8b,9b,AAAS,ABCA1,ABCA12,ABCA3,ABCA4,ABCB11,ABCB4,ABCB6,ABCC2,ABCC6,ABCC8,ABCC9,ABCD1,ABCD4,ABCG5,ABCG8,ABI1,ABI2,ABL1,ACACA,ACAN,ACE2,ACTB,ACTG1,ACTR2,ACTR3,ACY1,ADA,ADAM10,ADAM17,ADAMTS1,ADAMTS10,ADAMTS12,ADAMTS13,ADAMTS14,ADAMTS15,ADAMTS16,ADAMTS17,ADAMTS18,ADAMTS19,ADAMTS2,ADAMTS20,ADAMTS3,ADAMTS4,ADAMTS5,ADAMTS6,ADAMTS7,ADAMTS8,ADAMTS9,ADAMTSL1,ADAMTSL2,ADAMTSL3,ADAMTSL4,ADAMTSL5,ADCY1
R-HSA-1221632	Meiotic synapsis	ACD,ATR,BRCA1,DIDO1,FKBP6,H2AB1,H2AC14,H2AC18,H2AC20,H2AC4,H2AC6,H2AC7,H2AFX,H2AJ,H2AZ1,H2AZ2,H2BC1,H2BC11,H2BC12,H2BC12L,H2BC13,H2BC14,H2BC15,H2BC17,H2BC21,H2BC3,H2BC4,H2BC5,H2BC9,H2BU1,H3-4,H4C1,HSPA2,LMNA,LMNB1,POT1,RAD21,REC8,SMC1A,SMC1B,SMC3,STAG1,STAG2,STAG3,SUN1,SUN2,SYCE1,SYCE2,SYCE3,SYCP1,SYCP2,SYCP3,SYNE1,SYNE2,TERF1,TERF2,TERF2IP,TEX12,TINF2,UBE2I
R-HSA-168256	Immune System	8,9b,A1BG,AAAS,AAMP,ABCA13,ABCE1,ABI1,ABI2,ABL1,ABL2,ACAA1,ACLY,ACP3,ACTB,ACTG1,ACTR10,ACTR1A,ACTR1B,ACTR2,ACTR3,ADAM10,ADAM17,ADAM8,ADAR,ADGRE3,ADGRE5,ADGRG3,AGA,AGER,AGL,AGPAT2,AHCYL1,AHSG,AIM2,AIP,AKT1,AKT2,AKT3,ALAD,ALDH3B1,ALDOA,ALDOC,ALOX15,ALOX5,ALPK1,AMPD3,ANAPC1,ANAPC10,ANAPC11,ANAPC13,ANAPC2,ANAPC4,ANAPC5,ANAPC7,ANO6,ANPEP,ANXA1,ANXA2,AOC1,AP1B1,AP1G1,AP1M1,AP1M2,AP1S1,AP1S2,AP1S3,AP2A1
R-HSA-109581	Apoptosis	ACIN1,ADD1,AKT1,AKT2,AKT3,APAF1,APC,APIP,APPL1,ARHGAP10,AVEN,BAD,BAK1,BAX,BBC3,BCAP31,BCL2,BCL2L1,BCL2L11,BID,BIRC2,BMF,BMX,C1QBP,CARD8,CASP3,CASP6,CASP7,CASP8,CASP9,CD14,CDH1,CDKN2A,CFLAR,CLSPN,CTNNB1,CYCS,DAPK1,DAPK2,DAPK3,DBNL,DCC,DFFA,DFFB,DIABLO,DNM1L,DSG1,DSG2,DSG3,DSP,DYNLL1,DYNLL2,E2F1,FADD,FAS,FASLG,FNTA,GAS2,GSDMD,GSDME,GSN,GZMB,H1-0,H1-1,H1-2,H1-3,H1-4,H1-5
R-HSA-8863678	Neurodegenerative Diseases	APP,BCL2L11,CAPN1,CAPN2,CAPNS1,CAPNS2,CAST,CDC25A,CDC25B,CDC25C,CDK5,CDK5R1,FASLG,FOXO3,GOLGA2,JUN,LMNA,LMNB1,PRDX1,PRDX2,SOD2,YWHAE

R-HSA-9035034	RHOF GTPase cycle	ACTB,ACTN1,ADD3,AKAP12,ARHGAP1,ARHGAP12,ARHGAP21,ARHGAP32,ARHGAP39,ARHGAP5,BAIAP2L1,BAIAP2L2,BASP1,CAPZB,CAV1,DEPDC1B,DIAPH1,DIAPH2,DIAPH3,ESYT1,FAM169A,FARP1,LMNB1,MCAM,MTMR1,MYO9B,PIK3R1,PIK3R2,POTEE,RAB7A,RHOF,SENP1,SLC4A7,SNAP23,SOWAHC,SRGAP2,STEAP3,SYDE1,TMPO,TOR1AIP1,VAMP3,VANGL1
R-HSA-1280215	Cytokine Signaling in Immune system	8,AAAS,ABCE1,ABL2,ADAM17,ADAR,AGER,AIP,AKT1,AKT2,AKT3,ALOX15,ALOX5,ALPK1,ANXA1,ANXA2,APP,ARF1,ARIH1,ATF1,ATF2,B2M,BATF,BCL2,BCL2L1,BCL2L11,BCL6,BIRC2,BIRC3,BIRC5,BLNK,BOLA2,BRWD1,BST2,BTRC,CA1,CAMK2A,CAMK2B,CAMK2D,CAMK2G,CANX,CAPZA1,CASP1,CASP3,CASP8,CBL,CCL11,CCL19,CCL2,CCL20,CCL22,CCL3,CCL3L1,CCL4,CCL5,CCND1,CCR1,CCR2,CCR5,CD27,CD36,CD4,CD40,CD40LG,CD44,CD70,CD80,CD86
R-HSA-447115	Interleukin-12 family signaling	AIP,ANXA2,ARF1,BOLA2,CA1,CANX,CAPZA1,CDC42,CFL1,CNN2,CRLF1,EBI3,GSTA2,GSTO1,HNRNPA2B1,HNRNPDL,HNRNPF,HSP9,HSPA9,IFNG,IL10,IL12A,IL12B,IL12RB1,IL12RB2,IL23A,IL23R,IL27,IL27RA,IL6ST,JAK1,JAK2,LCP1,LMNB1,MIF,MSN,MTAP,P4HB,PAK2,PDCD4,PITPNA,PPIA,PSME2,RALA,RAP1B,RPLP0,SERPINB2,SNRPA1,SOD1,SOD2,STAT1,STAT3,STAT4,TALDO1,TCP1,TYK2,VAMP7,,
R-HSA-194315	Signaling by Rho GTPases	AAAS,ABCD3,ABI1,ABI2,ABL1,ABL2,ABR,ACBD5,ACTB,ACTC1,ACTG1,ACTN1,ACTR2,ACTR3,ADD3,AHCTF1,AKAP12,AKAP13,ALDH3A2,ALS2,AMIGO2,ANKFY1,ANKLE2,ANKRD26,ANLN,APITD1,AR,ARAP1,ARAP2,ARAP3,ARFGAP2,ARFGAP3,ARHGAP1,ARHGAP10,ARHGAP11A,ARHGAP11B,ARHGAP12,ARHGAP15,ARHGAP17,ARHGAP18,ARHGAP19,ARHGAP20,ARHGAP21,ARHGAP22,ARHGAP23,ARHGAP24,ARHGAP25,ARHGAP26,ARHGAP27,ARHGAP28,ARHGAP29,ARHGAP30,ARHGAP31,ARHGAP32,ARHGAP33,ARHGAP35,ARHGAP39,ARHGAP4,ARHGAP40,ARHGAP42,ARHGAP44,ARHGAP5,ARHGAP6,ARHGAP8,ARHGAP9,ARHGDIA,ARHGDIB,ARHGDIG
R-HSA-8953897	Cellular responses to stimuli	18SrRNA, 28SrRNA, 5.8SrRNA, 5S rRNA, AAAS, ABCC1, ACADVL, ACD, ACTR10, ACTR1A,ADD1,AGO1,AGO3,AGO4,AJUBA,AKT1,AKT1S1,AKT2,AKT3,ALB,ANAPC1, ANAPC10,ANAPC11,ANAPC15,ANAPC16,ANAPC2,ANAPC4,ANAPC5,ANAPC7,APOA1,APOB,AQP8,AR,ARFGAP1,ARNT,ARNTL,ASF1A,ASNS,ATF2,ATF3,ATF4,ATF5,ATF6, ATM,ATOX1,ATP6V0B,ATP6V0C,ATP6V0D1,ATP6V0D2,ATP6V0E1,ATP6V0E2,ATP6V1A,ATP6V1B1,ATP6V1B2,ATP6V1C1,ATP6V1C2,ATP6V1D,ATP6V1E1,ATP6V1E2,ATP6V1F,ATP6V1G1,ATP6V1G2,ATP6V1G3,ATP6V1H,ATP7A,ATPV0D1,ATR,BACH1
R-HSA-2559584	Formation of Senescence-Associated Heterochromatin Foci (SAHF)	ASF1A,CABIN1,CDKN1A,EP400,H1-0,H1-1,H1-2,H1-3,H1-4,H1-5,HIRA,HMGA1,HMGA2,LMNB1,RB1,TP53,UBN1
R-HSA-2555396	Mitotic Metaphase and Anaphase	AHCTF1,ANAPC1,ANAPC10,ANAPC11,ANAPC15,ANAPC16,ANAPC2,ANAPC4,ANAPC5,ANAPC7,ANKLE2,APITD1,AURKB,B9D2,BANF1,BIRC5,BUB1,BUB1B,BUB3,CC2D1B,CCNB1,CCNB2,CDC16,CDC20,CDC23,CDC26,CDC27,CDCA5,CDCA8,CDK1,CENPA,CENPC,CENPE,CENPF,CENPH,CENPI,CENPK,CENPL,CENPM,CENPN,CENPO,CENPP,CENPQ,CENPT,CENPU,CHMP2A,CHMP2B,CHMP3,CHMP4A,CHMP4B,CHMP4C,CHMP6,CHMP7,CKAP5,CLASP1,CLASP2,CLIP1,DSN1,DYNC1H1,DYNC1I1,DYNC1I2,DYNC1LI1,DYNC1LI2,DYNLL1,DYNLL2,EMD,ERCC6L,ESPL1
R-HSA-9020591	Interleukin-12 signaling	AIP,ANXA2,ARF1,BOLA2,CA1,CAPZA1,CDC42,CFL1,CNN2,GSTA2,GSTO1,HNRNPA2B1,HNRNPDL,HNRNPF,HSP9,HSPA9,IFNG,IL10,IL12A,IL12B,IL12RB1,IL12RB2,JAK1,JAK2,LCP1,LMNB1,MIF,MSN,MTAP,P4HB,PAK2,PDCD4,PITPNA,PPIA,PSME2,RALA,RAP1B,RPLP0,SERPINB2,SNRPA1,SOD1,SOD2,STAT4,TALDO1,TCP1,TYK2,VAMP7
R-HSA-68875	Mitotic Prophase	AAAS,ARPP19,BANF1,BLZF1,CCNB1,CCNB2,CDK1,CNEP1R1,CTDNEP1,EMD,ENSA,GOLGA2,GORASP1,GORASP2,H2AB1,H2AC14,H2AC18,H2AC20,H2AC4,H2AC6,H2AC7,H2AFX,H2AJ,H2AZ1,H2AZ2,H2BC1,H2BC11,H2BC12,H2BC12L,H2BC13,H2BC14,H2BC15,H2BC17,H2BC21,H2BC3,H2BC4,H2BC5,H2BC9,H2BU1,H3-3A,H3-4,H3C1,H3C15,H4C1,KMT5A,LEMD2,LEMD3,LMNA,LMNB1,LPIN1,LPIN2,LPIN3,MAPK1,MAPK3,MASTL,MCPH1,NCAPD3,NCAPG2,NCAPH2,NDC1,NEK6,NEK7,NEK9,NUMA1,NUP107,NUP133,NUP153,NUP155
R-HSA-4419969	Depolymerisation of the Nuclear Lamina	CCNB1,CDK1,CNEP1R1,CTDNEP1,EMD,LEMD2,LEMD3,LMNA,LMNB1,LPIN1,LPIN2,LPIN3,PRKCA,PRKCB,TMPO,
R-HSA-2559586	DNA Damage/Telomere Stress Induced Senescence	ACD,ASF1A,ATM,CABIN1,CCNA1,CCNA2,CCNE1,CCNE2,CDK2,CDKN1A,CDKN1B,EP400,H1-0,H1-1,H1-2,H1-3,H1-4,H1-5,H2AB1,H2AC14,H2AC18,H2AC20,H2AC4,H2AC6,H2AC7,H2AFX,H2AJ,H2AZ1,H2AZ2,H2BC1,H2BC11,H2BC12,H2BC12L,H2BC13,H2BC14,H2BC15,H2BC17,H2BC21,H2BC3,H2BC4,H2BC5,H2BC9,H2BU1,H34,H4C1,HIRA,HMGA1,HMGA2,KAT5,LMNB1,MRE11,NBN,POT1,RAD50,RB1,TERF1,TERF2,TERF2IP,TINF2,TP53,UBN1,,
R-HSA-2995383	Initiation of Nuclear Envelope (NE) Reformation	ANKLE2,BANF1,CCNB1,CCNB2,CDK1,EMD,KPNB1,LBR,LEMD2,LEMD3,LMNA,LMNB1,PPP2CA,PPP2R1A,PPP2R2A,SIRT2,TMPO,VRK1,VRK2,

R-HSA-2995410	Nuclear Envelope (NE) Reassembly	AHCTF1,ANKLE2,BANF1,CC2D1B,CCNB1,CCNB2,CDK1,CHMP2A,CHMP2B,CHMP3,CHMP4A,CHMP4B,CHMP4C,CHMP6,CHMP7,EMD,IST1,KPNB1,LBR,LEMD2,LEMD3,LMNA,LMNB1,NDC1,NUP107,NUP133,NUP155,NUP160,NUP188,NUP205,NUP35,NUP37,NUP43,NUP54,NUP58,NUP62,NUP85,NUP93,NUP98,POM121,PPP2CA,PPP2R1A,PPP2R2A,RAN,RANGAP1,RCC1,SEC13,SEH1L,SIRT2,SPAST,SUMO1,TMPO,TNPO1,TUBA1A,TUBA1B,TUBA1C,TUBA3C,TUBA3D,TUBA3E,TUBA4A,TUBA4B,TUBA8,TUBAL3,TUBB1,TUBB2A,TUBB2B,TUBB3,TUBB4A
R-HSA-1500620	Meiosis	ACD,ATM,ATR,BLM,BRCA1,BRCA2,CDK2,CDK4,DIDO1,DMC1,FKBP6,H2AB1,H2AC14,H2AC18,H2AC20,H2AC4,H2AC6,H2AC7,H2AFX,H2AJ,H2AZ1,H2AZ2,H2BC1,H2BC11,H2BC12,H2BC12L,H2BC13,H2BC14,H2BC15,H2BC17,H2BC21,H2BC3,H2BC4,H2BC5,H2BC9,H2BU1,H3-3A,H3-4,H3C1,H3C15,H4C1,HSPA2,LMNA,LMNB1,MLH1,MLH3,MND1,MRE11,MSH4,MSH5,NBN,POT1,PRDM9,PSMC3IP,RAD21,RAD50,RAD51,RAD51C,RBBP8,REC8,RPA1,RPA2,RPA3,SMC1A,SMC1B,SMC3,SPO11,STAG1
R-HSA-5357801	Programmed Cell Death	3a,ACIN1,ADD1,AKT1,AKT2,AKT3,APAF1,APC,APIP,APPL1,ARHGAP10,AVEN,BAD,BAK1,BAX,BBC3,BCAP31,BCL2,BCL2L1,BCL2L11,BID,BIRC2,BIRC3,BMF,BMX,C1QBP,CARD8,CASP1,CASP3,CASP4,CASP5,CASP6,CASP7,CASP8,CASP9,CD14,CDC37,CDH1,CDKN2A,CFLAR,CHMP2A,CHMP2B,CHMP3,CHMP4A,CHMP4B,CHMP4C,CHMP6,CHMP7,CLSPN,CRMA,CTNNB1,CYCS,DAPK1,DAPK2,DAPK3,DBNL,DCC,DFFA,DFFB,DIABLO,DNM1L,DSG1,DSG2,DSG3,DSP,DYNLL1,DYNLL2,E2F1
R-HSA-68886	M Phase	AAAS,ACTR1A,AHCTF1,AKAP9,ALMS1,ANAPC1,ANAPC10,ANAPC11,ANAPC15,ANAPC16,ANAPC2,ANAPC4,ANAPC5,ANAPC7,ANKLE2,APITD1,ARPP19,AURKB,B9D2,BANF1,BIRC5,BLZF1,BUB1,BUB1B,BUB3,CC2D1B,CCNB1,CCNB2,CCP110,CDC16,CDC20,CDC23,CDC26,CDC27,CDCA5,CDCA8,CDK1,CDK5RAP2,CENPA,CENPC,CENPE,CENPF,CENPH,CENPI,CENPJ,CENPK,CENPL,CENPM,CENPN,CENPO,CENPP,CENPQ,CENPT,CENPU,CEP131,CEP135,CEP152,CEP164,CEP192,CEP250,CEP290,CEP41,CEP43,CEP57,CEP63,CEP70,CEP72,CEP76
R-HSA-9716542	Signaling by Rho GTPases, Miro GTPases and RHOBTB3	ABL1,BIRC5,AR,RHOA,RHOB,RHOC,RND3,RHOG,ARHGDIA,RHOH,BUB1,BUB1B,FMNL1,CALM3,CDC20,CDC25C,CDC42,CDH1,CDKN1B,CENPA,CENPC,CENPE,CENPF,CFL1,CFTR,CTNNA1,CTNNB1,CYBA,CYBB,DIAPH1,DLG4,CTTN,FLNA,SFN,NCKAP1L,INCENP,ITGB1,KIF2A,KTN1,LIMK1,MAD2L1,MEN1,MYL6,MYLK,PPP1R12A,PPP1R12B,NCF2,NCF4,NF2,PAFAH1B1,PAK1,PAK2,PAK3,PDPK1,PIK3C3,PIN1,PLK1,PPP1CB,PPP1CC,PKN1,MAPK1,MAPK3,RAC1,RAC2,RAC3,RANBP2,RANGAP1,RAP1GDS1,RPS27,RTKN,CLIP1,S100A8,S100A9,SEC13,SRC,SRF,XPO1,YWHAB,YWHAE,YWHAG,YWHAH,YWHAZ,RND2,MAD1L1,IQGAP1,PRC1,ZW10,BUB3,AURKB,KNTC1,CKAP5,RHOBTB1,KIF14,ARPC5,ARPC4,ARPC3,ARPC1B,ACTR3,ACTR2,ARPC2,BAIAP2,NCOA2,ARPC1A,NUDC,NCKAP1,IQGAP2,NOXA1,YWHAQ,KIF2C,CIT,ZWINT,CDC37,RHOBTB3,MAPRE1,DAAM1,KDM1A,KDM4C,CLASP2,RHOBTB2,NUP160,CLASP1,SRGAP2,RHOQ,AHCTF1,NOX1,RND1,RHOD,PIK3R4,TAX1BP3,NOX3,EVL,RHOF,ROPN1,NDE1,ERCC6L,SPDL1,ZWILCH,CDCA8,RHOT1,NUP133,CENPN,BRK1,RCC2,KNL1,GOPC,NUP107,RHOJ,TAOK1,MRTFA,RHOU,LIN7B,CENPM,NUP37,NUP85,CENPT,B9D2,NDEL1,DIAPH3,KIF18A,KIF2B,RHPN2,RHOT2,FMNL3,CENPL,PPP1R14A,FMNL2,RHPN1,NOXO1,IQGAP3,SGO2,SGO1,RHOV,SKA1,SCAI,SKA2,NUP43,H4C15,NCF1,CENPS-CORT
R-HSA-2559583	Cellular Senescence	ACD,AGO1,AGO3,AGO4,ANAPC1,ANAPC10,ANAPC11,ANAPC15,ANAPC16,ANAPC2,ANAPC4,ANAPC5,ANAPC7,ASF1A,ATM,BMI1,CABIN1,CBX2,CBX4,CBX6,CBX8,CCNA1,CCNA2,CCNE1,CCNE2,CDC16,CDC23,CDC26,CDC27,CDK2,CDK4,CDK6,CDKN1A,CDKN1B,CDKN2A,CDKN2B,CDKN2C,CDKN2D,CEBPB,CXCL8,E2F1,E2F2,E2F3,EED,EHMT1,EHMT2,EP400,ERF,ETS1,ETS2,EZH2,FOS,FZR1,H1-0,H1-1,H1-2,H1-3,H1-4,H1-5,H2AB1,H2AC14,H2AC18,H2AC20,H2AC4,H2AC6,H2AC7,H2AFX,H2AJ
R-HSA-449147	Signaling by Interleukins	8,AGER,AIP,AKT1,ALOX15,ALOX5,ALPK1,ANXA1,ANXA2,APP,ARF1,ATF1,ATF2,BATF,BCL2,BCL2L1,BCL6,BIRC5,BLNK,BOLA2,BRWD1,BTRC,CA1,CANX,CAPZA1,CASP1,CASP3,CASP8,CBL,CCL11,CCL19,CCL2,CCL20,CCL22,CCL3,CCL3L1,CCL4,CCL5,CCND1,CCR1,CCR2,CCR5,CD36,CD4,CD80,CD86,CDC42,CDKN1A,CEBPD,CFL1,CHUK,CISH,CLCF1,CNN2,CNTF,CNTFR,COL1A2,CREB1,CRK,CRKL,CRLF1,CRLF2,CSF1,CSF1R,CSF2,CSF2 gene,CSF2RA,CSF2RB
R-HSA-1474165	Reproduction	ACD,ACR,ADAM2,ADAM20,ADAM21,ADAM30,ATM,ATR,B4GALT1,BLM,BRCA1,BRCA2,CATSPER1,CATSPER2,CATSPER3,CATSPER4,CATSPERB,CATSPERD,CATSPERG,CD9,CDK2,CDK4,DIDO1,DMC1,FKBP6,H2AB1,H2AC14,H2AC18,H2AC20,H2AC4,H2AC6,H2AC7,H2AFX,H2AJ,H2AZ1,H2AZ2,H2BC1,H2BC11,H2BC12,H2BC12L,H2BC13,H2BC14,H2BC15,H2BC17,H2BC21,H2BC3,H2BC4,H2BC5,H2BC9,H2BU1,H3-3A,H3-4,H3C1,H3C15,H4C1,HSPA2,HVCN1,IZUMO1,IZUMO2,IZUMO3,IZUMO4,KCNU1,LMNA,LMNB1,MLH1,MLH3,MND1,MRE11

R-HSA-2980766	Nuclear Envelope Breakdown	AAAS,BANF1,CCNB1,CCNB2,CDK1,CNEP1R1,CTDNEP1,EMD,LEMD2,LEMD3,LMNA,LMNB1,LPIN1,LPIN2,LPIN3,NDC1,NEK6,NEK7,NEK9,NUP107,NUP133,NUP153,NUP155,NUP160,NUP188,NUP205,NUP210,NUP214,NUP35,NUP37,NUP42,NUP43,NUP50,NUP54,NUP58,NUP62,NUP85,NUP88,NUP93,NUP98,PLK1,POM121,POM121C,PRKCA,PRKCB,RAE1,RANBP2,SEC13,SEH1L,TMPO,TPR,VRK1,VRK2,
R-HSA-9645723	Diseases of programmed cell death	APP,BCL2L11,C1QBP,CAPN1,CAPN2,CAPNS1,CAPNS2,CASP8,CAST,CDC25A,CDC25B,CDC25C,CDK5,CDK5R1,CDKN2A,DNMT1,DNMT3A,DNMT3B,EED,EZH2,FADD,FASLG,FOXO3,GOLGA2,GSDME,H2AB1,H2AC14,H2AC18,H2AC20,H2AC4,H2AC6,H2AC7,H2AFX,H2AJ,H2AZ1,H2AZ2,H2BC1,H2BC11,H2BC12,H2BC12L,H2BC13,H2BC14,H2BC15,H2BC17,H2BC21,H2BC3,H2BC4,H2BC5,H2BC9,H2BU1,H3-3A,H3C1,H3C15,H4C1,JUN,LMNA,LMNB1,POLA1,POLA2,PRDX1,PRDX2,PRIM1,PRIM2,RBBP4,RBBP7,RIPK1,SOD2,SUZ12
R-HSA-2262752	Cellular responses to stress	18SrRNA, 28SrRNA, 5.8rRNA, 5S rRNA, AAAS, ABCC1, ACADVL, ACD, ACTR10, ACTR1A,ADD1,AGO1,AGO3,AGO4,AJUBA,AKT1,AKT1S1,AKT2,AKT3,ALB,ANAPC1, ANAPC10,ANAPC11,ANAPC15,ANAPC16,ANAPC2,ANAPC4,ANAPC5,ANAPC7,APOA1,APOB,AQP8,AR,ARFGAP1,ARNT,ARNTL,ASF1A,ASNS,ATF2,ATF3,ATF4,ATF5,ATF6, ATM,ATOX1,ATP6V0B,ATP6V0C,ATP6V0D1,ATP6V0D2,ATP6V0E1,ATP6V0E2,ATP6V1A,ATP6V1B1,ATP6V1B2,ATP6V1C1,ATP6V1C2,ATP6V1D,ATP6V1E1,ATP6V1E2,ATP6V1F,ATP6V1G1,ATP6V1G2,ATP6V1G3,ATP6V1H,ATP7A,ATPV0D1,ATR,BACH1
R-HSA-68882	Mitotic Anaphase	AHCTF1,ANAPC1,ANAPC10,ANAPC11,ANAPC15,ANAPC16,ANAPC2,ANAPC4,ANAPC5,ANAPC7,ANKLE2,APITD1,AURKB,B9D2,BANF1,BIRC5,BUB1,BUB1B,BUB3,CC2D1B,CCNB1,CCNB2,CDC16,CDC20,CDC23,CDC26,CDC27,CDCA5,CDCA8,CDK1,CENPA,CENPC,CENPE,CENPF,CENPH,CENPI,CENPK,CENPL,CENPM,CENPN,CENPO,CENPP,CENPQ,CENPT,CENPU,CHMP2A,CHMP2B,CHMP3,CHMP4A,CHMP4B,CHMP4C,CHMP6,CHMP7,CKAP5,CLASP1,CLASP2,CLIP1,DSN1,DYNC1H1,DYNC1I1,DYNC1I2,DYNC1LI1,DYNC1LI2,DYNLL1,DYNLL2,EMD,ERCC6L,ESPL1
R-HSA-8950505	Gene and protein expression by JAK-STAT signaling after Interleukin-12 stimulation	AIP,ANXA2,ARF1,BOLA2,CA1,CAPZA1,CDC42,CFL1,CNN2,GSTA2,GSTO1,HNRNPA2B1,HNRNPDL,HNRNPF,HSP9,HSPA9,IFNG,IL10,LCP1,LMNB1,MIF,MSN,MTAP,PAK2,PDCD4,PITPNA,PPIA,PSME2,RALA,RAP1B,RPLP0,SERPINB2,SNRPA1,SOD1,SOD2,STAT4,TALDO1,TCP1
R-HSA-1640170	Cell Cycle	AAAS,ABL1,ACD,ACTR1A,AHCTF1,AJUBA,AKAP9,AKT1,AKT2,AKT3,ALMS1,ANAPC1,ANAPC10,ANAPC11,ANAPC15,ANAPC16,ANAPC2,ANAPC4,ANAPC5,ANAPC7,ANKLE2,ANKRD28,APITD1,ARPP19,ATM,ATR,ATRIP,ATRX,AURKA,AURKB,B9D2,BABAM1,BANF1,BARD1,BIRC5,BLM,BLZF1,BORA,BRCA1,BRCA2,BRCC3,BRE,BRIP1,BTRC,BUB1,BUB1B,BUB3,CABLES1,CC2D1B,CCNA1,CCNA2,CCNB1,CCNB2,CCND1,CCND2,CCND3,CCNE1,CCNE2,CCNH,CCP110,CDC14A,CDC16,CDC20,CDC23,CDC25A,CDC25B,CDC25C,CDC26
R-HSA-9012999	RHO GTPase cycle	AAAS,ABCD3,ABI1,ABI2,ABL2,ABR,ACBD5,ACTB,ACTC1,ACTG1,ACTN1,ADD3,AKAP12,AKAP13,ALDH3A2,ALS2,AMIGO2,ANKFY1,ANKLE2,ANKRD26,ANLN,ARAP1,ARAP2,ARAP3,ARFGAP2,ARFGAP3,ARHGAP1,ARHGAP10,ARHGAP11A,ARHGAP11B,ARHGAP12,ARHGAP15,ARHGAP17,ARHGAP18,ARHGAP19,ARHGAP20,ARHGAP21,ARHGAP22,ARHGAP23,ARHGAP24,ARHGAP25,ARHGAP26,ARHGAP27,ARHGAP28,ARHGAP29,ARHGAP30,ARHGAP31,ARHGAP32,ARHGAP33,ARHGAP35,ARHGAP39,ARHGAP4,ARHGAP40,ARHGAP42,ARHGAP44,ARHGAP5,ARHGAP6,ARHGAP8,ARHGAP9,ARHGDIA,ARHGDIB,ARHGDIG,ARHGEF1,ARHGEF10,ARHGEF10L,ARHGEF11,ARHGEF12,ARHGEF15

R-HSA-69278	Cell Cycle, Mitotic	BIRC5,CCND1,BUB1,BUB1B,CCNA2,CCNB1,CCND2,CCND3,CCNE1,CCNH,CDK1,CDC6,CDC20,CDC25A,CDC25B,CDC25C,CDC27,CDK2,CDK4,CDK6,CDK7,CDKN1A,CDKN1B,CENPA,CENPC,CENPE,CENPF,CETN2,RCC1,CKS1B,CSNK1D,CSNK2B,DHFR,DNA2,DYNC1H1,DYNC1I2,DYRK1A,E2F1,E2F4,ENSA,EP300,FEN1,FOXM1,GOLGA2,GSK3B,HDAC1,HMMR,HSP90AA1,HSP90AB1,INCENP,KIF2A,KPNB1,LBR,LIG1,LMNB1,MAD2L1,MAX,MCM2,MCM3,MCM4,MCM5,MCM6,MCM7,RAB8A,MNAT1,MYBL2,MYC,PPP1R12A,NEK2,NUMA1,NUP88,ODF2,ORC1,ORC2,ORC4,ORC5,PAFAH1B1,PCM1,PCNA,PCNT,PLK1,POLA1,POLD1,POLD2,POLE,POLE2,PPP1CB,PPP1CC,PPP2CA,PPP2R1A,PPP2R2A,PRIM1,PRIM2,PRKACA,PRKAR2B,MAPK1,PSMA1,PSMA2,PSMA3,PSMA4,PSMA5,PSMA6,PSMA7,PSMB1,PSMB2,PSMB3,PSMB4,PSMB5,PSMB6,PSMB7,PSMB8,PSMB9,PSMB10,PSMC1,PSMC2,PSMC3,PSMC4,PSMC5,PSMC6,PSMD1,PSMD2,PSMD3,PSMD4,PSMD5,PSMD7,PSMD8,PSMD9,PSMD10,PSMD11,PSMD12,PSMD13,PSME1,PSME2,PTK6,RAB2A,RAD21,RAN,RANBP2,RANGAP1,RB1,RBBP4,RBL1,RBL2,RFC1,RFC2,RFC3,RFC4,RFC5,RPA1,RPA2,RPA3,RPS27,RRM2,CLIP1,SEC13,SET,SKP1,SKP2,SPAST,AURKA,TK1,TOP2A,TP53,TPR,TUBA4A,TUBG1,TYMS,UBE2D1,UBE2E1,UBE2I,SUMO1,WEE1,XPO1,YWHAE,YWHAG,ALMS1,TUBA1A,SEM1,NUP214,AAAS,SMC1A,CDC7,CDC45,MAD1L1,CUL1,RAE1,OFD1,BLZF1,USO1,SSNA1,DYNLL1,CDC23,BANF1,CDC16,CCNA1,BTRC,PKMYT1,SMC3,CCNB2,ZW10,BUB3,AURKB,PTTG1,PSMF1,KIF23,NUP155,CEP135,NUP93,ESPL1,CEP57,KNTC1,CCP110,CKAP5,IST1,SFI1,GINS1,PSMD6,POM121,NCAPD2,NUP153,RBX1,SMC4,KIF20A,ACTR1A,OPTN,AKAP9,PSME3,PSMD14,STAG1,TUBB4A,TUBB4B,ANAPC10,TUBGCP3,DCTN2,SMC2,POLD3,NUDC,PLK4,STAG2,NUP50,ARPP19,SDCCAG8,TUBGCP2,DBF4,KIF2C,CNTRL,UBE2C,NUP42,CEP43,ZWINT,CEP250,DCTN3,PHLDA1,CEP164,MAPRE1,SIRT2,TPX2,NINL,CEP131,CEP152,WAPL,CLASP2,ANKLE2,NUP205,FBXL7,PSME4,NUP210,NUP160,FBXW11,NCAPD3,CLASP1,HAUS5,MAU2,NCAPH,CTDNEP1,NUP188,ORC6,ORC3,NUP62,POLA2,NIPBL,ANAPC15,AHCTF1,CHMP2B,GORASP2,FBXO5,TUBG2,VPS4A,TUBGCP4,CHMP2A,UBE2S,EML4,PPP2R3B,CHMP4A,NCAPH2,ANAPC2,NME7,RPA4,ANAPC4,GMNN,FZR1,PPME1,ANAPC5,ANAPC7,LCMT1,GTSE1,ANAPC11,GINS2,NUP54,POLE3,HAUS6,NDE1,ERCC6L,NCAPG2,SPDL1,HAUS4,ZWILCH,CEP192,HAUS2,CDCA8,MCM10,HAUS7,NDC1,CEP72,NUP133,CDK5RAP2,CENPJ,CENPN,PPP2R2D,RCC2,LIN37,POLE4,KNL1,NUP107,TAOK1,POLD4,FKBPL,NCAPG,ANAPC1,GORASP1,GINS3,CENPM,NUP37,HAUS3,CHMP6,MCPH1,BORA,NUP85,CEP76,MZT2B,CENPT,CEP290,CEP63,CEP70,B9D2,NDEL1,CDT1,KIF18A,CEP78,GINS4,MCM8,KIF2B,MASTL,AJUBA,TUBGCP6,CENPL,LIN52,NEK9,CABLES1,CHMP7,CHMP4C,HAUS8,CEP41,CDCA5,TUBGCP5,HAUS1,ANAPC16,NEDD1,PSMB11,CHMP4B,NUP35,LIN54,PSMA8,SGO2,SGO1,CC2D1B,TUBB,SKA1,LEMD2,CDC26,CNEP1R1,LIN9,SKA2,NUP43,KMT5A,MZT1,H4C15,MZT2A,CENPS-CORT,RNF103-CHMP3,TPTEP2-CSNK1E
R-HSA-8862803	Deregulated CDK5 triggers multiple neurodegenerative pathways in Alzheimer's disease models	APP,BCL2L11,CAPN1,CAPN2,CAPNS1,CAPNS2,CAST,CDC25A,CDC25B,CDC25C,CDK5,CDK5R1,FASLG,FOXO3,GOLGA2,JUN,LMNA,LMNB1,PRDX1,PRDX2,SOD2,YWHAE
R-HSA-9734009	Defective Intrinsic Pathway for Apoptosis	APP,BCL2L11,C1QBP,CAPN1,CAPN2,CAPNS1,CAPNS2,CAST,CDC25A,CDC25B,CDC25C,CDK5,CDK5R1,CDKN2A,FASLG,FOXO3,GOLGA2,JUN,LMNA,LMNB1,PRDX1,PRDX2,SOD2,TP53,YWHAE

**Immune TFs**		
**ETS2**		
R-HSA-2559583	Cellular Senescence	ACD,AGO1,AGO3,AGO4,ANAPC1,ANAPC10,ANAPC11,ANAPC15,ANAPC16,ANAPC2,ANAPC4,ANAPC5,ANAPC7,ASF1A,ATM,BMI1,CABIN1,CBX2,CBX4,CBX6,CBX8,CCNA1,CCNA2,CCNE1,CCNE2,CDC16,CDC23,CDC26,CDC27,CDK2,CDK4,CDK6,CDKN1A,CDKN1B,CDKN2A,CDKN2B,CDKN2C,CDKN2D,CEBPB,CXCL8,E2F1,E2F2,E2F3,EED,EHMT1,EHMT2,EP400,ERF,ETS1,ETS2,EZH2,FOS,FZR1,H1-0,H1-1,H1-2,H1-3,H1-4,H1-5,H2AB1,H2AC14,H2AC18,H2AC20,H2AC4,H2AC6,H2AC7,H2AFX,H2AJ
R-HSA-2559585	Oncogene Induced Senescence	AGO1,AGO3,AGO4,CDK4,CDK6,CDKN2A,CDKN2B,CDKN2C,CDKN2D,E2F1,E2F2,E2F3,ERF,ETS1,ETS2,ID1,MAPK1,MAPK3,MDM2,MDM4,MIR241,MIR242,MOV10,RB1,RPS27A,SP1,TFDP1,TFDP2,TNRC6A,TNRC6B,TNRC6C,TP53,UBA52,UBB,UBC,

R-HSA-8953897	Cellular responses to stimuli	18SrRNA, 28SrRNA, 5.8SrRNA, 5S rRNA, AAAS, ABCC1, ACADVL, ACD, ACTR10,ACTR1A,ADD1,AGO1,AGO3,AGO4,AJUBA,AKT1,AKT1S1,AKT2,AKT3, ALB,ANAPC1,ANAPC10,ANAPC11,ANAPC15,ANAPC16,ANAPC2,ANAPC4,ANAPC5,ANAPC7,APOA1,APOB,AQP8,AR,ARFGAP1,ARNT,ARNTL,ASF1A,ASNS,ATF2,ATF3,ATF4,ATF5,ATF6,ATM,ATOX1,ATP6V0B,ATP6V0C,ATP6V0D1,ATP6V0D2,ATP6V0E1,ATP6V0E2,ATP6V1A,ATP6V1B1,ATP6V1B2,ATP6V1C1,ATP6V1C2,ATP6V1D,ATP6V1E1,ATP6V1E2,ATP6V1F,ATP6V1G1,ATP6V1G2,ATP6V1G3,ATP6V1H,ATP7A,ATPV0D1,ATR,BACH1
R-HSA-2262752	Cellular responses to stress	18SrRNA, 28SrRNA, 5.8SrRNA, 5SrRNA, AAAS, ABCC1, ACADVL, ACD, ACTR10, ACTR1A,ADD1,AGO1,AGO3,AGO4,AJUBA,AKT1,AKT1S1,AKT2,AKT3,ALB,ANAPC1,ANAPC10,ANAPC11,ANAPC15,ANAPC16,ANAPC2,ANAPC4,ANAPC5,ANAPC7,APOA1,APOB,AQP8,AR,ARFGAP1,ARNT,ARNTL,ASF1A,ASNS,ATF2,ATF3,ATF4,ATF5,ATF6,ATM,ATOX1,ATP6V0B,ATP6V0C,ATP6V0D1,ATP6V0D2,ATP6V0E1,ATP6V0E2,ATP6V1A,ATP6V1B1,ATP6V1B2,ATP6V1C1,ATP6V1C2,ATP6V1D,ATP6V1E1,ATP6V1E2,ATP6V1F,ATP6V1G1,ATP6V1G2,ATP6V1G3,ATP6V1H,ATP7A,ATPV0D1,ATR,BACH1
**BACH1**		
R-HSA-9711123	Cellular response to chemical stress	ABCC1,AKT1,AKT2,AKT3,ALB,AQP8,ATF4,ATOX1,ATP7A,BACH1,BLVRA,BLVRB,BTRC,CARM1,CAT,CCS,CDKN2A,CHD9,COX11,COX14,COX16,COX18,COX19,COX20,COX4I1,COX5A,COX5B,COX6A1,COX6B1,COX6C,COX7A2L,COX7B,COX7C,COX8A,CREBBP,CSNK2A1,CSNK2A2,CSNK2B,CUL1,CUL3,CYBA,CYBB,CYCS,EP300,ERO1A,FABP1,FBXL17,G6PD,GCLC,GCLM,GPX1,GPX2,GPX3, GPX5,GPX6,GPX7,GPX8,GSK3B,GSR,GSTA1,GSTA3,GSTP1,HBA1,HBB,HDAC3, HELZ2,HM13,HMOX1
R-HSA-9708530	Regulation of BACH1 activity	BACH1,CUL1,FBXL17,MAFK,MIR155,MIR196A1,MIR98,MIRLET7B,MIRLET7C, RBX1,RPS27A,SKP1,SKP2,UBA52,UBB,UBC
R-HSA-9707564	Cytoprotection by HMOX1	ABCC1,ALB,BACH1,BLVRA,BLVRB,CARM1,CHD9,COX11,COX14,COX16,COX18,COX19,COX20,COX4I1,COX5A,COX5B,COX6A1,COX6B1,COX6C,COX7A2L, COX7B,COX7C,COX8A,CREBBP,CYCS,FABP1,HBA1,HBB,HDAC3,HELZ2,HM13,HMOX1,HMOX2,LRPPRC,MAFK,MED1,MT-CO1, MTCO2, MTCO3, NCOA1, NCOA2, NCOA6, NCOR1, NCOR2, NDUFA4, NFE2L2,NLRP3,PPARA,PTK6,RXRA,SCO1,SCO2,SIN3A,SIN3B,SMARCD3,STAP2,STAT3,SURF1,TACO1,TBL1X,TBL1XR1,TGS1,TXNIP
R-HSA-9707587	Regulation of HMOX1 expression and activity	BACH1,HM13,HMOX1,MAFK,NFE2L2
R-HSA-9707616	Heme signaling	APOA1,APOB,ARNTL,ATF2,BACH1,CARM1,CHD9,CLEC1B,CLOCK,CREB1,CREBBP,CRTC1,CRTC2,CRTC3,EP300,HBA1,HBB,HDAC3,HELZ2,HMOX1,LY96,MAFK,MED1,MEF2C,MEF2D,NCOA1,NCOA2,NCOA6,NCOR1,NFE2L2,NPAS2,NR1D1,NRIP1,PPARA,PPARGC1,PSPHP1,RAI1,RORA,RXRA,SIRT1,SLC46A1,SMARCD3,TBL1X,TBL1XR1,TGS1,TLR4,XPO1
R-HSA-8953897	Cellular responses to stimuli	18SrRNA, 28SrRNA, 5.8SrRNA, 5S rRNA, AAAS, ABCC1, ACADVL, ACD, ACTR10, ACTR1A, ADD1, AGO1, AGO3, AGO4, AJUBA, AKT1, AKT1S1, AKT2, AKT3,ALB,ANAPC1,ANAPC10,ANAPC11,ANAPC15,ANAPC16,ANAPC2,ANAPC4,ANAPC5,ANAPC7,APOA1,APOB,AQP8,AR,ARFGAP1,ARNT,ARNTL,ASF1A,ASNS,ATF2,ATF3,ATF4,ATF5,ATF6,ATM,ATOX1,ATP6V0B,ATP6V0C,ATP6V0D1, ATP6V0D2,ATP6V0E1,ATP6V0E2,ATP6V1A,ATP6V1B1,ATP6V1B2,ATP6V1C1,ATP6V1C2,ATP6V1D,ATP6V1E1,ATP6V1E2,ATP6V1F,ATP6V1G1,ATP6V1G2,ATP6V1G3,ATP6V1H,ATP7A,ATPV0D1,ATR,BACH1
R-HSA-2262752	Cellular responses to stress	18SrRNA, 28SrRNA, 5.8SrRNA, 5S rRNA, AAAS, ABCC1, ACADVL, ACD, ACTR10, ACTR1A, ADD1, AGO1, AGO3, AGO4, AJUBA, AKT1, AKT1S1, AKT2, AKT3,ALB,ANAPC1,ANAPC10,ANAPC11,ANAPC15,ANAPC16,ANAPC2,ANAPC4,ANAPC5,ANAPC7,APOA1,APOB,AQP8,AR,ARFGAP1,ARNT,ARNTL,ASF1A,ASNS,ATF2,ATF3,ATF4,ATF5,ATF6,ATM,ATOX1,ATP6V0B,ATP6V0C,ATP6V0D1, ATP6V0D2,ATP6V0E1,ATP6V0E2,ATP6V1A,ATP6V1B1,ATP6V1B2,ATP6V1C1,ATP6V1C2,ATP6V1D,ATP6V1E1,ATP6V1E2,ATP6V1F,ATP6V1G1,ATP6V1G2,ATP6V1G3,ATP6V1H,ATP7A,ATPV0D1,ATR,BACH1
**STAT1**		
R-HSA-9673770	Signaling by PDGFRA extracellular domain mutants	GRB2,HRAS,KRAS,NRAS,PDGFRA,PIK3CA,PIK3CB,PIK3R1,PIK3R2,SOS1,STAT1,STAT3,

R-HSA-913531	Interferon Signaling	AAAS,ABCE1,ADAR,ARIH1,B2M,BST2,CAMK2A,CAMK2B,CAMK2D,CAMK2G,CD44,CIITA,DDX58,EGR1,EIF2AK2,EIF4A1,EIF4A2,EIF4A3,EIF4E,EIF4E2,EIF4E3,EIF4G1,EIF4G2,EIF4G3,FCGR1A,FCGR1B,FLNA,FLNB,GBP1,GBP2,GBP3,GBP4,GBP5,GBP6,GBP7,HERC5,HLA-A,HLA-B,HLA-C,HLA-DPA1,HLA-DPB1,HLA-DQA1,HLA-DQA2,HLA-DQB1,HLA-DQB2,HLA-DRA,HLA-DRB1,HLA-DRB3,HLA-DRB4,HLA-DRB5,HLA-E,HLA-F,HLA-G,HLAH, ICAM1, IFI27, IFI30, IFI35,IFI6,IFIT1,IFIT2,IFIT3,IFIT5,IFITM1,IFITM2,IFITM3,IFNA1,IFNA10
R-HSA-9674555	Signaling by CSF3 (G-CSF)	CSF3,CSF3R,CUL5,ELOB,ELOC,GAB2,GRB2,HCK,JAK1,JAK2,KRAS,LYN,PTPN11,RNF7,RPS27A,SHC1,SOCS1,SOCS3,STAT1,STAT3,STAT5A,STAT5B,SYK,TYK2,UBA52,UBB,UBC,UBE2D1,UBE2D2,UBE2D3,
R-HSA-9669938	Signaling by KIT in disease	FYN,GRB2,HRAS,JAK2,KIT,KRAS,LCK,LYN,NRAS,PIK3CA,PIK3R1,PIK3R2,PIK3R3,SOS1,SRC,STAT1,STAT3,STAT5A,STAT5B,YES1
R-HSA-9012852	Signaling by NOTCH3	ADAM10,APH1A,APH1B,CREBBP,DLGAP5,DLL1,DLL4,EGF,EGFR,EP300,FABP7,HES1,HES5,HEY1,HEY2,HEYL,IKZF1,JAG1,JAG2,KAT2A,KAT2B,MAML1,MAML2,MAML3,MAMLD1,MIB1,MIB2,NCSTN,NEURL1,NEURL1B,NOTCH1,NOTCH3,PBX1,PLXND1,PSEN1,PSEN2,PSENEN,PTCRA,RBPJ,RPS27A,SNW1,STAT1,TACC3,UBA52,UBB,UBC,WWC1,WWP2, YBX1,
R-HSA-157118	Signaling by NOTCH	ACTA2,ADAM10,ADAM17,AGO1,AGO2,AGO3,AGO4,AKT1,APH1A,APH1B,ARRB1,ARRB2,ATP2A1,ATP2A2,ATP2A3,B4GALT1,CCNC,CCND1,CDK8,CNTN1,CREB1,CREBBP,CUL1,DLGAP5,DLK1,DLL1,DLL4,DNER,DTX1,DTX2,DTX4,E2F1,E2F3,EGF,EGFR,ELF3,EP300,FABP7,FBXW7,FCER2,FLT4,FURIN,GZMB,H2AB1,H2AC14,H2AC18,H2AC20,H2AC4,H2AC6,H2AC7,H2AFX,H2AJ,H2AZ1,H2AZ2,H2BC1,H2BC11,H2BC12,H2BC12L,H2BC13,H2BC14,H2BC15,H2BC17,H2BC21,H2BC3,H2BC4,H2BC5,H2BC9,H2BU1
R-HSA-168256	Immune System	8,9b,A1BG,AAAS,AAMP,ABCA13,ABCE1,ABI1,ABI2,ABL1,ABL2,ACAA1,ACLY,ACP3,ACTB,ACTG1,ACTR10,ACTR1A,ACTR1B,ACTR2,ACTR3,ADAM10,ADAM17,ADAM8,ADAR,ADGRE3,ADGRE5,ADGRG3,AGA,AGER,AGL,AGPAT2,AHCYL1,AHSG,AIM2,AIP,AKT1,AKT2,AKT3,ALAD,ALDH3B1,ALDOA,ALDOC,ALOX15,ALOX5,ALPK1,AMPD3,ANAPC1,ANAPC10,ANAPC11,ANAPC13,ANAPC2,ANAPC4,ANAPC5,ANAPC7,ANO6,ANPEP,ANXA1,ANXA2,AOC1,AP1B1,AP1G1,AP1M1,AP1M2,AP1S1,AP1S2,AP1S3,AP2A1
R-HSA-1433557	Signaling by SCF-KIT	CBL,CHEK1,CMA1,FER,FES,FYN,GAB2,GRAP,GRAP2,GRB10,GRB2,GRB7,HRAS,JAK2,KIT,KITLG,KRAS,LCK,LYN,MMP9,NRAS,PIK3CA,PIK3R1,PIK3R2,PIK3R3,PRKCA,PTPN11,PTPN6,PTPRU,RAC1,SH2B2,SH2B3,SOCS1,SOCS6,SOS1,SRC,STAT1,STAT3,STAT5A,STAT5B,TEC,VAV1,YES1,
R-HSA-8984722	Interleukin-35 Signalling	CANX,EBI3,IL12A,IL12RB2,IL27RA,IL6ST,JAK1,JAK2,STAT1,STAT3,STAT4,TYK2,
R-HSA-9013508	NOTCH3 Intracellular Domain Regulates Transcription	CREBBP,DLGAP5,EP300,FABP7,HES1,HES5,HEY1,HEY2,HEYL,IKZF1,KAT2A,KAT2B,MAML1,MAML2,MAML3,MAMLD1,NOTCH1,NOTCH3,PBX1,PLXND1,PTCRA,RBPJ,SNW1,STAT1,WWC1
R-HSA-912694	Regulation of IFNA/IFNB signaling	IFNA1,IFNA10,IFNA14,IFNA16,IFNA17,IFNA2,IFNA21,IFNA4,IFNA5,IFNA6,IFNA7,IFNA8,IFNAR1,IFNAR2,IFNB1,JAK1,PTPN1,PTPN11,PTPN6,SOCS1,SOCS3,STAT1,STAT2,TYK2,USP18
R-HSA-9673767	Signaling by PDGFRA transmembrane, juxtamembrane and kinase domain mutants	PIK3CB,PIK3R2,STAT1,SOS1,PIK3CA,KRAS,PDGFRA,PIK3R1,STAT3,HRAS,GRB2,NRAS,HRAS
R-HSA-982772	Growth hormone receptor signaling	ADAM17,CISH,CSH1,GH1,GH2,GHR,IRS1,IRS2,JAK2,LYN,MAPK1,MAPK3,PRL,PRLR,PTPN1,PTPN6,SH2B1,SOCS1,SOCS2,SOCS3,STAT1,STAT3,STAT5A,STAT5B,
R-HSA-9020956	Interleukin-27 signaling	CANX,CRLF1,EBI3,IL27,IL27RA,IL6ST,JAK1,JAK2,STAT1,STAT3,TYK2,,
R-HSA-9671555	Signaling by PDGFR in disease	BIN2,ETV6,FIP1L1,GOLGA4,GRB2,HRAS,KANK1,KDR,KRAS,NRAS,PDGFRA,PIK3CA,PIK3CB,PIK3R1,PIK3R2,SOS1,STAT1,STAT3,STRN,WDR48

R-HSA-186763	Downstream signal transduction	BCAR1,CRK,CRKL,GRB2,GRB7,HRAS,KRAS,NCK1,NCK2,NRAS,PDGFA,PDGFB,PDGFRA,PDGFRB,PIK3CA,PIK3CB,PIK3R1,PIK3R2,PLCG1,PTPN11,RAPGEF1,RASA1,SOS1,SRC,STAT1,STAT3,STAT5A,STAT5B,STAT6
R-HSA-909733	Interferon alpha/beta signaling	ABCE1,ADAR,BST2,EGR1,GBP2,HLA-A,HLA-B,HLA-C,HLA-E,HLA-F,HLA-G,HLA-H, IFI27,IFI35,IFI6,IFIT1,IFIT2,IFIT3,IFIT5,IFITM1,IFITM2,IFITM3,IFNA1,IFNA10,IFNA14,IFNA16,IFNA17,IFNA2,IFNA21,IFNA4,IFNA5,IFNA6,IFNA7,IFNA8,IFNAR1,IFNAR2,IFNB1,IP6K2,IRF1,IRF2,IRF3,IRF4,IRF5,IRF6,IRF7,IRF8,IRF9,ISG15,ISG20,JAK1,KPNA1,KPNB1,MX1,MX2,N,OAS1,OAS2,OAS3,OASL,PSMB8,PTPN1,PTPN11,PTPN6,RNASEL,RSAD2,SAMHD1,SOCS1,SOCS3
R-HSA-8878166	Transcriptional regulation by RUNX2	ABL1,AKT1,AKT2,AKT3,AR,BAX,BGLAP,BMP2,CBFB,CCNB1,CCND1,CDK1,CDK4,CDKN1A,COL1A1,CUL1,DLX5,DLX6,ESR1,ESRRA,FBXW7,GLI2,GLI3,GSK3B,HAND2,HDAC3,HDAC4,HDAC6,HES1,HEY1,HEY2,HIVEP3,IHH,ITGA5,ITGBL1,LGALS3,MAF,MAPK1,MAPK3,MMP13,MSX2,NKX3-2, NR3C1, PPARGC1A, PPARGC1B, PPM1D, PSMA1, PSMA2, PSMA3,PSMA4,PSMA5,PSMA6,PSMA7,PSMA8,PSMB1,PSMB10,PSMB11,PSMB2,PSMB3,PSMB4,PSMB5,PSMB6,PSMB7,PSMB8,PSMB9,PSMC1,PSMC2,PSMC3
R-HSA-449147	Signaling by Interleukins	8,AGER,AIP,AKT1,ALOX15,ALOX5,ALPK1,ANXA1,ANXA2,APP,ARF1,ATF1,ATF2,BATF,BCL2,BCL2L1,BCL6,BIRC5,BLNK,BOLA2,BRWD1,BTRC,CA1,CANX,CAPZA1,CASP1,CASP3,CASP8,CBL,CCL11,CCL19,CCL2,CCL20,CCL22,CCL3,CCL3L1,CCL4,CCL5,CCND1,CCR1,CCR2,CCR5,CD36,CD4,CD80,CD86,CDC42,CDKN1A,CEBPD,CFL1,CHUK,CISH,CLCF1,CNN2,CNTF,CNTFR,COL1A2,CREB1,CRK,CRKL,CRLF1,CRLF2,CSF1,CSF1R,CSF2,CSF2 gene,CSF2RA,CSF2RB
R-HSA-9705462	Inactivation of CSF3 (G-CSF) signaling	CSF3,CSF3R,CUL5,ELOB,ELOC,HCK,JAK1,JAK2,LYN,RNF7,RPS27A,SOCS1,SOCS3,STAT1,STAT3,STAT5A,STAT5B,SYK,TYK2,UBA52,UBB,UBC,UBE2D1,UBE2D2,UBE2D3
R-HSA-1643685	Disease	18S rRNA,1a,28S rRNA,3a,5.8S rRNA,5S rRNA,6,7SL RNA (ENSG00000222619),7SL RNA (ENSG00000222639),7a,8,8b,9b,AAAS,ABCA1,ABCA12,ABCA3,ABCA4,ABCB11,ABCB4,ABCB6,ABCC2,ABCC6,ABCC8,ABCC9,ABCD1,ABCD4,ABCG5,ABCG8,ABI1,ABI2,ABL1,ACACA,ACAN,ACE2,ACTB,ACTG1,ACTR2,ACTR3,ACY1,ADA,ADAM10,ADAM17,ADAMTS1,ADAMTS10,ADAMTS12,ADAMTS13,ADAMTS14,ADAMTS15,ADAMTS16,ADAMTS17,ADAMTS18,ADAMTS19,ADAMTS2,ADAMTS20,ADAMTS3,ADAMTS4,ADAMTS5,ADAMTS6,ADAMTS7,ADAMTS8,ADAMTS9,ADAMTSL1,ADAMTSL2,ADAMTSL3,ADAMTSL4,ADAMTSL5,ADCY1
R-HSA-212436	Generic Transcription Pathway	ABCA6,ABL1,ACTL6A,ACTL6B,AGO1,AGO2,AGO3,AGO4,AGRP,AIFM2,AKT1,AKT2,AKT3,ANAPC1,ANAPC10,ANAPC11,ANAPC15,ANAPC16,ANAPC2,ANAPC4,ANAPC5,ANAPC7,APAF1,APOE,AR,ARID1A,ARID1B,ARID2,ARID3A,ASH2L,ATAD2,ATF2,ATM,ATP1B4,ATR,ATRIP,ATXN3,AURKA,AURKB,AUTS2,AXIN1,BANP,BARD1,BAX,BBC3,BCL2L11,BCL2L14,BCL6,BDNF,BGLAP,BID,BIRC5,BLK,BLM,BMI1,BMP2,BNIP3L,BRCA1,BRD1,BRD2,BRD7,BRIP1,BRPF1,BRPF3,BTG1,BTG2,CALM1,CAMK2A
R-HSA-1839117	Signaling by cytosolic FGFR1 fusion mutants	BCR,CEP43,CNTRL,CPSF6,CUX1,FGFR1OP2,GAB2,GRB2,LRRFIP1,MYO18A,PIK3CA,PIK3R1,STAT1,STAT3,STAT5A,STAT5B,TRIM24,ZMYM2
R-HSA-1169410	Antiviral mechanism by IFN-stimulated genes	AAAS,ABCE1,ARIH1,DDX58,EIF2AK2,EIF4A1,EIF4A2,EIF4A3,EIF4E,EIF4E2,EIF4E3,EIF4G1,EIF4G2,EIF4G3,FLNA,FLNB,HERC5,IFIT1,IRF3,ISG15,JAK1,KPNA1,KPNA2,KPNA3,KPNA4,KPNA5,KPNA7,KPNB1,MAPK3,MX1,MX2,NDC1,NEDD4,NS,NUP107,NUP133,NUP153,NUP155,NUP160,NUP188,NUP205,NUP210,NUP214,NUP35,NUP37,NUP42,NUP43,NUP50,NUP54,NUP58,NUP62,NUP85,NUP88,NUP93,NUP98,OAS1,OAS2,OAS3,OASL,PDE12,PIN1,PLCG1,POM121,POM121C,PPM1B,RAE1,RANBP2,RNASEL
R-HSA-1169408	ISG15 antiviral mechanism	AAAS,ARIH1,DDX58,EIF2AK2,EIF4A1,EIF4A2,EIF4A3,EIF4E,EIF4E2,EIF4E3,EIF4G1,EIF4G2,EIF4G3,FLNB,HERC5,IFIT1,IRF3,ISG15,JAK1,KPNA1,KPNA2,KPNA3,KPNA4,KPNA5,KPNA7,KPNB1,MAPK3,MX1,MX2,NDC1,NEDD4,NS,NUP107,NUP133,NUP153,NUP155,NUP160,NUP188,NUP205,NUP210,NUP214,NUP35,NUP37,NUP42,NUP43,NUP50,NUP54,NUP58,NUP62,NUP85,NUP88,NUP93,NUP98,PIN1,PLCG1,POM121,POM121C,PPM1B,RAE1,RANBP2,RPS27A,SEC13,SEH1L,STAT1,TPR,TRIM25,UBA52,UBA7

R-HSA-447115	Interleukin-12 family signaling	AIP,ANXA2,ARF1,BOLA2,CA1,CANX,CAPZA1,CDC42,CFL1,CNN2,CRLF1,EBI3,GSTA2,GSTO1,HNRNPA2B1,HNRNPDL,HNRNPF,HSP9,HSPA9,IFNG,IL10,IL12A,IL12B,IL12RB1,IL12RB2,IL23A,IL23R,IL27,IL27RA,IL6ST,JAK1,JAK2,LCP1,LMNB1,MIF,MSN,MTAP,P4HB,PAK2,PDCD4,PITPNA,PPIA,PSME2,RALA,RAP1B,RPLP0,SERPINB2,SNRPA1,SOD1,SOD2,STAT1,STAT3,STAT4,TALDO1,TCP1,TYK2,VAMP7
R-HSA-1059683	Interleukin-6 signaling	CBL,IL6,IL6R,IL6ST,JAK1,JAK2,PTPN11,SOCS3,STAT1,STAT3,TYK2,,
R-HSA-6785807	Interleukin-4 and Interleukin-13 signaling	AKT1,ALOX15,ALOX5,ANXA1,BATF,BCL2,BCL2L1,BCL6,BIRC5,CCL11,CCL2,CCL22,CCND1,CD36,CDKN1A,CEBPD,COL1A2,CXCL8,F13A1,FASLG,FCER2,FGF2,FN1,FOS,FOXO1,FOXO3,FSCN1,GATA3,HGF,HIF1A,HMOX1,HSP90AA1,HSP90B1,HSPA8,ICAM1,IGHE,IGHG1,IGHG4,IL10,IL12A,IL12B,IL13,IL13RA1,IL13RA2,IL17A,IL17F,IL18,IL1A,IL1B,IL23A,IL23R,IL2RG,IL4,IL4R,IL6,IL6R,IL8,IRF4,ITGAM,ITGAX,ITGB1,ITGB2,JAK1,JAK2,JAK3,JUNB,LAMA5,LBP
R-HSA-5663202	Diseases of signal transduction by growth factor receptors and second messengers	ADAM10,ADAM17,AGGF1,AGK,AGTRAP,AKAP9,AKT1,AKT1S1,AKT2,AKT3,ALK,AMER1,AP3B1,APBB1IP,APC,APH1A,APH1B,ARAF,AREG,ARRB1,ARRB2,ATG7,ATIC,AXIN1,BAD,BAG4,BCL11A,BCL2A1,BCL2L1,BCL2L11,BCR,BIN2,BIRC6,BRAF,BRAP,BTC,CALM1,CAMK2A,CAMK2B,CAMK2D,CAMK2G,CARS1,CASP9,CBL,CCNC,CD19,CD28,CD80,CD86,CDC37,CDK8,CDKN1A,CDKN1B,CEBPB,CEP43,CHUK,CLCN6,CLTC,CNKSR1,CNKSR2,CNTRL,CPSF6,CREB1,CREBBP,CSK,CSNK1A1,CTBP1,CTBP2
R-HSA-5655302	Signaling by FGFR1 in disease	BAG4,BCR,CEP43,CNTRL,CPSF6,CUX1,ERLIN2,FGF1,FGF17,FGF2,FGF20,FGF23,FGF4,FGF5,FGF6,FGF8,FGF9,FGFR1,FGFR1OP2,FRS2,GAB1,GAB2,GRB2,HRAS,KRAS,LRRFIP1, MYO18A,NRAS,PIK3CA,PIK3R1,PLCG1,SOS1,STAT1,STAT3,STAT5A,STAT5B,TRIM24,ZMYM2
R-HSA-186797	Signaling by PDGF	BCAR1,COL4A1,COL4A2,COL4A3,COL4A4,COL4A5,COL6A1,COL6A2,COL6A3,COL6A5,COL6A6,COL9A1,COL9A2,COL9A3,CRK,CRKL,FURIN,GRB2,GRB7,HRAS,KRAS,NCK1,NCK2,NRAS,PDGFA,PDGFB,PDGFC,PDGFD,PDGFRA,PDGFRB,PIK3CA,PIK3CB,PIK3R1,PIK3R2,PLAT,PLCG1,PLG,PTPN11,PTPN12,RAPGEF1,RASA1,SOS1,SPP1,SRC,STAT1,STAT3,STAT5A,STAT5B,STAT6,THBS1,THBS2,THBS3,THBS4,
R-HSA-1839124	FGFR1 mutant receptor activation	BAG4,BCR,CEP43,CNTRL,CPSF6,CUX1,ERLIN2,FGF1,FGF17,FGF2,FGF20,FGF23,FGF4,FGF5,FGF6,FGF8,FGF9,FGFR1,FGFR1OP2,GAB2,GRB2,LRRFIP1,MYO18A,PIK3CA,PIK3R1,STAT1,STAT3,STAT5A,STAT5B,TRIM24,ZMYM2,
R-HSA-74160	Gene expression (Transcription)	18SrRNA, 5.8SrRNA, 28SrRNA, 28SrRNA, 45Spre-rRNA, 5.8SrRNA, 5S rRNA, AAAS, ABCA6, ABL1,ACTB,ACTL6A,ACTL6B,AEBP2,AFF4,AGO1,AGO2,AGO3,AGO4,AGRP,AIFM2,AKT1,AKT2,AKT3,ALYREF,ANAPC1,ANAPC10,ANAPC11,ANAPC15,ANAPC16,ANAPC2,ANAPC4,ANAPC5,ANAPC7,ANG,APAF1,APOE,AR,ARID1A,ARID1B,ARID2,ARID3A,ARID4B,ASH2L,ASUN,ASZ1,ATAD2,ATF2,ATM,ATP1B4,ATR,ATRIP,ATXN3,AURKA,AURKB,AUTS2,AXIN1,BANP,BARD1,BAX,BAZ1B,BAZ2A,BBC3,BCDIN3D,BCL2L11,BCL2L14,BCL6,BDNF
R-HSA-1226099	Signaling by FGFR in disease	BAG4,BCR,CEP43,CNTRL,CPSF6,CUX1,ERLIN2,FGF1,FGF10,FGF16,FGF17,FGF18,FGF2,FGF20,FGF22,FGF23,FGF3,FGF4,FGF5,FGF6,FGF7,FGF8,FGF9,FGFR1,FGFR1OP2,FGFR2,FGFR3,FGFR4,FRS2,GAB1,GAB2,GRB2,GTF2F1,GTF2F2,HRAS,KRAS,LRRFIP1,MYO18A,NCBP1,NCBP2,NRAS,PIK3CA,PIK3R1,PLCG1,POLR2A,POLR2B,POLR2C,POLR2D,POLR2E,POLR2F,POLR2G,POLR2H,POLR2I,POLR2J,POLR2K,POLR2L,SOS1,STAT1,STAT3,STAT5A,STAT5B,TRIM24,ZMYM2
R-HSA-451927	Interleukin-2 family signaling	CSF2,CSF2RA,CSF2RB,GAB2,GRB2,HAVCR2,IL15,IL15RA,IL2,IL21,IL21R,IL2RA,IL2RB,IL2RG,IL3,IL3RA,IL5,IL5RA,IL9,IL9R,INPP5D,INPPL1,JAK1,JAK2,JAK3,LCK,LGALS9,PIK3CA,PIK3CB,PIK3CD,PIK3R1,PIK3R2,PIK3R3,PTK2B,PTPN6,SHC1,SOS1,SOS2,STAT1,STAT3,STAT4,STAT5A,STAT5B,SYK,
R-HSA-1280215	Cytokine Signaling in Immune system	8,AAAS,ABCE1,ABL2,ADAM17,ADAR,AGER,AIP,AKT1,AKT2,AKT3,ALOX15,ALOX5,ALPK1,ANXA1,ANXA2,APP,ARF1,ARIH1,ATF1,ATF2,B2M,BATF,BCL2,BCL2L1,BCL2L11,BCL6,BIRC2,BIRC3,BIRC5,BLNK,BOLA2,BRWD1,BST2,BTRC,CA1,CAMK2A,CAMK2B,CAMK2D,CAMK2G,CANX,CAPZA1,CASP1,CASP3,CASP8,CBL,CCL11,CCL19,CCL2,CCL20,CCL22,CCL3,CCL3L1,CCL4,CCL5,CCND1,CCR1,CCR2,CCR5,CD27,CD36,CD4,CD40,CD40LG,CD44,CD70,CD80,CD86

R-HSA-162582	Signal Transduction	AAAS,AAMP,AATF,ABCA1,ABCD3,ABCG1,ABCG5,ABCG8,ABHD12,ABHD17A,ABHD17B,ABHD17C,ABHD6,ABI1,ABI2,ABL1,ABL2,ABR,ACBD5,ACKR1,ACKR2,ACKR3,ACKR4,ACTA2,ACTB,ACTC1,ACTG1,ACTN1,ACTN2,ACTR2,ACTR3,ACVR1B,ACVR1C,ACVR2A,ACVR2B,ACVRL1,ADAM10,ADAM12,ADAM17,ADAP1,ADCY1,ADCY10,ADCY2,ADCY3,ADCY4,ADCY5,ADCY6,ADCY7,ADCY8,ADCY9,ADCYAP1,ADCYAP1R1,ADD3,ADGRE1,ADGRE2,ADGRE3,ADGRE5,ADH1A,ADH1C,ADH4,ADM,ADM2,ADORA1,ADORA2A,ADORA2B,ADORA3,ADRA1A,ADRA1B
R-HSA-73857	RNA Polymerase II Transcription	ABCA6,ABL1,ACTL6A,ACTL6B,AFF4,AGO1,AGO2,AGO3,AGO4,AGRP,AIFM2,AKT1,AKT2,AKT3,ALYREF,ANAPC1,ANAPC10,ANAPC11,ANAPC15,ANAPC16,ANAPC2,ANAPC4,ANAPC5,ANAPC7,APAF1,APOE,AR,ARID1A,ARID1B,ARID2,ARID3A,ASH2L,ASUN,ATAD2,ATF2,ATM,ATP1B4,ATR,ATRIP,ATXN3,AURKA,AURKB,AUTS2,AXIN1,BANP,BARD1,BAX,BBC3,BCL2L11,BCL2L14,BCL6,BDNF,BGLAP,BID,BIRC5,BLK,BLM,BMI1,BMP2,BNIP3L,BRCA1,BRD1,BRD2,BRD7,BRIP1,BRPF1,BRPF3,BTG1
R-HSA-9020958	Interleukin-21 signaling	IL21,IL21R,IL2RG,JAK1,JAK3,STAT1,STAT3,STAT4,STAT5A,STAT5B
R-HSA-6783589	Interleukin-6 family signaling	CBL,CLCF1,CNTF,CNTFR,CRLF1,CTF1,IL11,IL11RA,IL31,IL31RA,IL6,IL6R,IL6ST,JAK1,JAK2,LIF,LIFR,OSM,OSMR,PTPN11,SOCS3,STAT1,STAT3,TYK2,
R-HSA-9006934	Signaling by Receptor Tyrosine Kinases	AAMP,ABI1,ABI2,ADAM10,ADAM12,ADAM17,ADAP1,ADCYAP1,ADCYAP1R1,ADORA2A,AHCYL1,AKT1,AKT2,AKT3,ALK,ANOS1,AP2A1,AP2A2,AP2B1,AP2M1,AP2S1,APH1A,APH1B,APOE,ARC,AREG,ARF6,ARHGEF7,ASCL1,ATF1,ATF2,ATP6AP1,ATP6V0A1,ATP6V0A2,ATP6V0A4,ATP6V0B,ATP6V0C,ATP6V0D1,ATP6V0D2,ATP6V0E1,ATP6V0E2,ATP6V1A,ATP6V1B1,ATP6V1B2,ATP6V1C1,ATP6V1C2,ATP6V1D,ATP6V1E1,ATP6V1E2,ATP6V1F,ATP6V1G1,ATP6V1G2,ATP6V1G3,ATP6V1H,AXL,BAIAP2,BAX,BCAR1,BDNF,BRAF,BRK1,BTC,CALM1,CAV1,CBL,CD274,CDC37,CDC42
R-HSA-8939902	Regulation of RUNX2 expression and activity	BMP2,CBFB,CUL1,DLX5,DLX6,ESR1,ESRRA,FBXW7,GSK3B,HIVEP3,MSX2,NKX3-2, NR3C1,PPARGC1A,PPARGC1B,PSMA1,PSMA2,PSMA3,PSMA4,PSMA5,PSMA6,PSMA7,PSMA8,PSMB1,PSMB10,PSMB11,PSMB2,PSMB3,PSMB4,PSMB5,PSMB6,PSMB7,PSMB8,PSMB9,PSMC1,PSMC2,PSMC3,PSMC4,PSMC5,PSMC6,PSMD1,PSMD10,PSMD11,PSMD12,PSMD13,PSMD14,PSMD2,PSMD3,PSMD4,PSMD5,PSMD6,PSMD7,PSMD8,PSMD9,PSME1,PSME2,PSME3,PSME4,PSMF1,RBX1,RPS27A,RUNX2,SEM1,SKP1,SKP2,SMURF1,STAT1,STUB1
R-HSA-8985947	Interleukin-9 signaling	IL2RG,IL9,IL9R,JAK1,JAK3,STAT1,STAT3,STAT5A,STAT5B,
R-HSA-8854691	Interleukin-20 family signaling	IFNL1,IFNL2,IFNL3,IFNLR1,IL10RB,IL19,IL20,IL20RA,IL20RB,IL22,IL22RA1,IL22RA2,IL24,IL26,JAK1,JAK2,JAK3,PTPN11,SOCS3,STAT1,STAT2,STAT3,STAT4,STAT5A,STAT5B,TYK2

**Ribosomal TFs**		
**OGT**		
R-HSA-5688426	Deubiquitination	ACTB,ACTL6A,ACTR5,ACTR8,ADRB2,ADRM1,APC,AR,ARRB1,ARRB2,ASXL1,ASXL2,ATXN3,ATXN3L,ATXN7,AXIN1,AXIN2,BABAM1,BAP1,BARD1,BECN1,BIRC2,BIRC3,BRCA1,BRCC3,BRE,CCNA1,CCNA2,CCP110,CDC20,CDC25A,CDK1,CFTR,CLSPN,CYLD,DDB2,DDX58,EP300,ESR1,FAM175A,FAM175B,FKBP8,FOXK1,FOXK2,FOXO4,GATA3,H2AC1,H2AC11,H2AC12,H2AC14,H2AC18,H2AC20,H2AC21,H2AC4,H2AC6,H2AC7,H2AW,H2BC1,H2BC11,H2BC12,H2BC13,H2BC14,H2BC15,H2BC17,H2BC18,H2BC21,H2BC3,H2BC4
R-HSA-5689603	UCH proteinases	ACTB,ACTL6A,ACTR5,ACTR8,ADRM1,ASXL1,ASXL2,BAP1,BARD1,FOXK1,FOXK2,H2AC1,H2AC11,H2AC12,H2AC14,H2AC18,H2AC20,H2AC21,H2AC4,H2AC6,H2AC7,H2AW,HCFC1,INO80,INO80B,INO80C,INO80D,INO80E,KDM1B,MBD5,MBD6,MCRS1,NEDD8,NFRKB,OGT,PSMA1,PSMA2,PSMA3,PSMA4,PSMA5,PSMA6,PSMA7,PSMA8,PSMB1,PSMB10,PSMB11,PSMB2,PSMB3,PSMB4,PSMB5,PSMB6,PSMB7,PSMB8,PSMB9,PSMC1,PSMC2,PSMC3,PSMC4,PSMC5,PSMC6,PSMD1, PSMD10,PSMD11,PSMD12,PSMD13,PSMD14,PSMD2,PSMD3

R-HSA-5218859	Regulated Necrosis	3a,BAK1,BAX,BIRC2,BIRC3,CASP1,CASP3,CASP4,CASP5,CASP8,CDC37,CFLAR,CHMP2A,CHMP2B,CHMP3,CHMP4A,CHMP4B,CHMP4C,CHMP6,CHMP7,CRMA,CYCS,ELANE,FADD,FAS,FASLG,FLOT1,FLOT2,GSDMD,GSDME,GZMB,HMGB1,HSP90AA1,IL18,IL1A,IL1B,IRF1,IRF2,MLKL,NS,OGT,PDCD6IP,PELI1,RIPK1,RIPK3,RIR1,RPS27A,SDCBP,SPI-2,STUB1,TNFRSF10A,TNFRSF10B,TNFSF10,TP53,TP63,TRADD,TRAF2,UBA52,UBB,UBC,UL36,XIAP,
R-HSA-3214847	HATs acetylate histones	ACTB,ACTL6A,ATF2,ATXN7,ATXN7L3,BRD1,BRD8,BRPF1,BRPF3,CLOCK,CREBBP,DMAP1,DR1,ELP2,ELP3,ELP4,ELP5,ELP6,ENY2,EP300,EP400,EPC1,H2AC1,H2AC11,H2AC12,H2AC14,H2AC18,H2AC20,H2AC21,H2AC4,H2AC6,H2AC7,H2AW,H2BC1,H2BC11,H2BC12,H2BC13,H2BC14,H2BC15,H2BC17,H2BC18,H2BC21,H2BC3,H2BC4,H2BC5,H2BC9,H2BU1,H3C1,H3C15,H4C1,HAT1,HCFC1,IKBKAP,ING3,ING4,ING5,JADE1,JADE2,JADE3,KANSL1,KANSL2,KANSL3,KAT14,KAT2A,KAT2B,KAT5,KAT6A,KAT6B
R-HSA-5675482	Regulation of necroptotic cell death	BIRC2,BIRC3,CASP8,CDC37,CFLAR,CRMA,FADD,FAS,FASLG,FLOT1,FLOT2,HSP90AA1,MLKL,OGT,PDCD6IP,PELI1,RIPK1,RIPK3,RPS27A,SDCBP,SPI-2,STUB1,TNFRSF10A,TNFRSF10B,TNFSF10,TRADD,TRAF2,UBA52,UBB,UBC,XIAP,
R-HSA-5357801	Programmed Cell Death	3a,ACIN1,ADD1,AKT1,AKT2,AKT3,APAF1,APC,APIP,APPL1,ARHGAP10,AVEN,BAD,BAK1,BAX,BBC3,BCAP31,BCL2,BCL2L1,BCL2L11,BID,BIRC2,BIRC3,BMF,BMX,C1QBP,CARD8,CASP1,CASP3,CASP4,CASP5,CASP6,CASP7,CASP8,CASP9,CD14,CDC37,CDH1,CDKN2A,CFLAR,CHMP2A,CHMP2B,CHMP3,CHMP4A,CHMP4B,CHMP4C,CHMP6,CHMP7,CLSPN,CRMA,CTNNB1,CYCS,DAPK1,DAPK2,DAPK3,DBNL,DCC,DFFA,DFFB,DIABLO,DNM1L,DSG1,DSG2,DSG3,DSP,DYNLL1,DYNLL2,E2F1
R-HSA-5213460	RIPK1-mediated regulated necrosis	3a,BIRC2,BIRC3,CASP8,CDC37,CFLAR,CRMA,FADD,FAS,FASLG,FLOT1,FLOT2,HSP90AA1,MLKL,NS,OGT,PDCD6IP,PELI1,RIPK1,RIPK3,RIR1,RPS27A,SDCBP,SPI-2, STUB1, TNFRSF10A, TNFRSF10B, TNFSF10, TRADD, TRAF2, UBA52,UBB,UBC,UL36,XIAP,
R-HSA-392499	Metabolism of proteins	18S rRNA,28S rRNA,5.8S rRNA,5S rRNA, 7SLRNA, A4GNT, AAAS, AARS1, AARS2,ABCA3,ACE,ACE2,ACHE,ACTB,ACTL6A,ACTR10,ACTR1A,ACTR5,ACTR8,ADAM10,ADAMTS1,ADAMTS10,ADAMTS12,ADAMTS13,ADAMTS14,ADAMTS15,ADAMTS16,ADAMTS17,ADAMTS18,ADAMTS19,ADAMTS2,ADAMTS20,ADAMTS3,ADAMTS4,ADAMTS5,ADAMTS6,ADAMTS7,ADAMTS8,ADAMTS9,ADAMTSL1,ADAMTSL2,ADAMTSL3,ADAMTSL4,ADAMTSL5,ADGRF5,ADORA2A,ADORA2B,ADRA2A,ADRA2C,ADRB2,ADRM1,AFP,AGBL1,AGBL2,AGBL3,AGBL4,AGBL5,AGT,AGTPBP1,AHSG,AIMP1,AIMP2,ALB,ALG1,ALG10,ALG10B,ALG11
R-HSA-597592	Post-translational protein modification	A4GNT,AAAS,ACTB,ACTL6A,ACTR10,ACTR1A,ACTR5,ACTR8,ADAM10,ADAMTS1,ADAMTS10,ADAMTS12,ADAMTS13,ADAMTS14,ADAMTS15,ADAMTS16,ADAMTS17,ADAMTS18,ADAMTS19,ADAMTS2,ADAMTS20,ADAMTS3,ADAMTS4,ADAMTS5,ADAMTS6,ADAMTS7,ADAMTS8,ADAMTS9,ADAMTSL1,ADAMTSL2,ADAMTSL3,ADAMTSL4,ADAMTSL5,ADRB2,ADRM1,AFP,AGBL1,AGBL2,AGBL3,AGBL4,AGBL5,AGTPBP1,AHSG,ALB,ALG1,ALG10,ALG10B,ALG11,ALG12,ALG13,ALG14,ALG2,ALG3,ALG5,ALG6,ALG8,ALG9,ALPG,ALPI,ALPL,AMBN,AMDHD2,AMELX,AMFR,AMTN,ANK1,ANK2,ANK3
R-HSA-4839726	Chromatin organizatio	ACTB,ACTL6A,ACTL6B,AEBP2,ARID1A,ARID1B,ARID2,ARID4A,ARID4B,ARID5B,ASH1L,ASH2L,ATF2,ATF7IP,ATXN7,ATXN7L3,BRD1,BRD8,BRMS1,BRPF1,BRPF3,BRWD1,CARM1,CCND1,CDK4,CHD3,CHD4,CLOCK,COPRS,CREBBP,DMAP1,DNMT3A,DOT1L,DPY30,DR1,EED,EHMT1,EHMT2,ELP2,ELP3,ELP4,ELP5,ELP6,ENY2,EP300,EP400,EPC1,EZH2,GATAD2A,GATAD2B,GPS2,H2AB1,H2AC1,H2AC11,H2AC12,H2AC14,H2AC18,H2AC20,H2AC21,H2AC4,H2AC6,H2AC7,H2AFX,H2AJ,H2AW,H2AZ1,H2AZ2,H2BC1
R-HSA-3247509	Chromatin modifying enzymes	ACTB,ACTL6A,ACTL6B,AEBP2,ARID1A,ARID1B,ARID2,ARID4A,ARID4B,ARID5B,ASH1L,ASH2L,ATF2,ATF7IP,ATXN7,ATXN7L3,BRD1,BRD8,BRMS1,BRPF1,BRPF3,BRWD1,CARM1,CCND1,CDK4,CHD3,CHD4,CLOCK,COPRS,CREBBP,DMAP1,DNMT3A,DOT1L,DPY30,DR1,EED,EHMT1,EHMT2,ELP2,ELP3,ELP4,ELP5,ELP6,ENY2,EP300,EP400,EPC1,EZH2,GATAD2A,GATAD2B,GPS2,H2AB1,H2AC1,H2AC11,H2AC12,H2AC14,H2AC18,H2AC20,H2AC21,H2AC4,H2AC6,H2AC7,H2AFX,H2AJ,H2AW,H2AZ1,H2AZ2,H2BC1
**PRKDC**		

R-HSA-392499	Metabolism of proteins	18S rRNA,28S rRNA,5.8S rRNA,5S rRNA, 7SLRNA, A4GNT, AAAS, AARS1, AARS2,ABCA3,ACE,ACE2,ACHE,ACTB,ACTL6A,ACTR10,ACTR1A,ACTR5,ACTR8,ADAM10,ADAMTS1,ADAMTS10,ADAMTS12,ADAMTS13,ADAMTS14,ADAMTS15,ADAMTS16,ADAMTS17,ADAMTS18,ADAMTS19,ADAMTS2,ADAMTS20,ADAMTS3,ADAMTS4,ADAMTS5,ADAMTS6,ADAMTS7,ADAMTS8,ADAMTS9,ADAMTSL1,ADAMTSL2,ADAMTSL3,ADAMTSL4,ADAMTSL5,ADGRF5,ADORA2A,ADORA2B,ADRA2A,ADRA2C,ADRB2,ADRM1,AFP,AGBL1,AGBL2,AGBL3,AGBL4,AGBL5,AGT,AGTPBP1,AHSG,AIMP1,AIMP2,ALB,ALG1,ALG10,ALG10B,ALG11
R-HSA-597592	Post-translational protein modification	A4GNT,AAAS,ACTB,ACTL6A,ACTR10,ACTR1A,ACTR5,ACTR8,ADAM10,ADAMTS1,ADAMTS10,ADAMTS12,ADAMTS13,ADAMTS14,ADAMTS15,ADAMTS16,ADAMTS17,ADAMTS18,ADAMTS19,ADAMTS2,ADAMTS20,ADAMTS3,ADAMTS4,ADAMTS5,ADAMTS6,ADAMTS7,ADAMTS8,ADAMTS9,ADAMTSL1,ADAMTSL2,ADAMTSL3,ADAMTSL4,ADAMTSL5,ADRB2,ADRM1,AFP,AGBL1,AGBL2,AGBL3,AGBL4,AGBL5,AGTPBP1,AHSG,ALB,ALG1,ALG10,ALG10B,ALG11,ALG12,ALG13,ALG14,ALG2,ALG3,ALG5,ALG6,ALG8,ALG9,ALPG,ALPI,ALPL,AMBN,AMDHD2,AMELX,AMFR,AMTN,ANK1,ANK2,ANK3
R-HSA-1834941	STING mediated induction of host immune responses	CGAS,DDX41,DTX4,IFI16,IRF3,MRE11,NLRC3,NLRP4,PRKDC,STAT6,STING1,TBK1,TREX1,TRIM21,XRCC5,XRCC6
R-HSA-3270619	IRF3-mediated induction of type I IFN	DDX41,DTX4,IFI16,IRF3,MRE11,NLRC3,NLRP4,PRKDC,STING1,TBK1,TREX1,XRCC5,XRCC6
R-HSA-5693532	DNA Double-Strand Break Repair	ABL1,APBB1,ATM,ATR,ATRIP,BABAM1,BAP1,BARD1,BAZ1B,BLM,BRCA1,BRCA2,BRCC3,BRE,BRIP1,CCNA1,CCNA2,CDK2,CHEK1,CHEK2,CLSPN,DCLRE1C,DNA2,EME1,EME2,ERCC1,ERCC4,EXO1,EYA1,EYA2,EYA3,EYA4,FAM175A,FEN1,GEN1,H2AFX,H2BC1,H2BC11,H2BC12,H2BC12L,H2BC13,H2BC14,H2BC15,H2BC17,H2BC21,H2BC3,H2BC4,H2BC5,H2BC9,H2BU1,H3-4,H4C1,HERC2,HUS1,KAT5,KDM4A,KDM4B,KPNA2,LIG3,LIG4,MAPK8,MDC1,MRE11,MUS81,NBN,NHEJ1,NSD2,PALB2
R-HSA-5693571	Nonhomologous End-Joining (NHEJ)	ATM,BABAM1,BARD1,BRCA1,BRCC3,BRE,DCLRE1C,FAM175A,H2AFX,H2BC1,H2BC11,H2BC12,H2BC12L,H2BC13,H2BC14,H2BC15,H2BC17,H2BC21,H2BC3,H2BC4,H2BC5,H2BC9,H2BU1,H3-4,H4C1,HERC2,KAT5,LIG4,MDC1,MRE11,NBN,NHEJ1,NSD2,PAXIP1,PIAS4,POLL,POLM,PRKDC,RAD50,RIF1,RNF168,RNF8,TDP1,TDP2,TP53BP1,UBE2N,UBE2V2,UIMC1,XRCC4,XRCC5,XRCC6
R-HSA-73894	DNA Repair	ABL1,ACD,ACTB,ACTL6A,ACTR5,ACTR8,ADPRS,ALKBH2,ALKBH3,ALKBH5,APBB1,APEX1,APITD1,AQR,ASCC1,ASCC2,ASCC3,ATM,ATR,ATRIP,BABAM1,BAP1,BARD1,BAZ1B,BLM,BRCA1,BRCA2,BRCC3,BRE,BRIP1,CCNA1,CCNA2,CCNH,CDK2,CDK7,CETN2,CHD1L,CHEK1,CHEK2,CLSPN,COPS2,COPS3,COPS4,COPS5,COPS6,COPS7A,COPS7B,COPS8,CUL4A,CUL4B,DCLRE1A,DCLRE1B,DCLRE1C,DDB1,DDB2,DNA2,DTL,ELL,EME1,EME2,EP300,ERCC1,ERCC2,ERCC3,ERCC4,ERCC5,ERCC6,ERCC8
R-HSA-8852135	Protein ubiquitination	BCL10,CDC34,CDC73,CTR9,DERL1,H2BC1,H2BC11,H2BC12,H2BC13,H2BC14,H2BC15,H2BC17,H2BC3,H2BC4,H2BC5,H2BC9,HLA-A,HLA-B,HLTF,LEO1,OTULIN,PAF1,PCNA,PEX10,PEX12,PEX13,PEX14,PEX2,PEX5,PRKDC,RAD18,RNF144A,RNF152,RNF181,RNF20,RNF40,RPS27A,RRAGA,RTF1,SELENOS,SHPRH,TMEM129,UBA1,UBA52,UBA6,UBB,UBC,UBE2A,UBE2B,UBE2C,UBE2D1,UBE2D2,UBE2D3,UBE2E1,UBE2E3,UBE2G1,UBE2G2,UBE2H,UBE2J2,UBE2K,UBE2L3,UBE2N,UBE2Q2,UBE2R2,UBE2S,UBE2T,UBE2V2,UBE2W
R-HSA-8866654	E3 ubiquitin ligases ubiquitinate target proteins	BCL10,CDC73,CTR9,DERL1,H2BC1,H2BC11,H2BC12,H2BC13,H2BC14,H2BC15,H2BC17,H2BC3,H2BC4,H2BC5,H2BC9,HLA-A,HLA-B,HLTF,LEO1,PAF1,PCNA,PEX10,PEX12,PEX13,PEX14,PEX2,PEX5,PRKDC,RAD18,RNF144A,RNF152,RNF181,RNF20,RNF40,RPS27A,RRAGA,RTF1,SELENOS,SHPRH,TMEM129,UBA52,UBB,UBC,UBE2A,UBE2B,UBE2D1,UBE2D2,UBE2D3,UBE2E1,UBE2J2,UBE2L3,UBE2N,UBE2V2,US11,VCP,WAC,WDR61
R-HSA-168249	Innate Immune System	9b,A1BG,AAMP,ABCA13,ABI1,ABI2,ABL1,ACAA1,ACLY,ACP3,ACTB,ACTG1,ACTR10,ACTR1B,ACTR2,ACTR3,ADAM10,ADAM8,ADGRE3,ADGRE5,ADGRG3,AGA,AGER,AGL,AGPAT2,AHCYL1,AHSG,AIM2,ALAD,ALDH3B1,ALDOA,ALDOC,ALOX5,ALPK1,AMPD3,ANO6,ANPEP,ANXA2,AOC1,AP1M1,AP2A2,APAF1,APEH,APOB,APP,APRT,ARG1,ARHGAP9,ARL8A,ARMC8,ARPC1A,ARPC1B,ARPC2,ARPC3,ARPC4,ARPC5,ARSA,ARSB,ART1,ASAH1,ATAD3B,ATF1,ATF2,ATG12,ATG5,ATG7,ATOX1,ATP11A

R-HSA-1834949	Cytosolic sensors of pathogen-associated DNA	AIM2,CGAS,CHUK,CRCP,CREBBP,CTNNB1,DDX41,DHX36,DHX9,DTX4,EP300,IFI16,IKBKB,IKBKG,IRF3,IRF7,LRRFIP1,MRE11,MYD88,NFKB1,NFKB2,NFKBIA,NFKBIB,NKIRAS1,NKIRAS2,NLRC3,NLRP4,POLR1C,POLR1D,POLR2E,POLR2F,POLR2H,POLR2K,POLR2L,POLR3A,POLR3B,POLR3C,POLR3D,POLR3E,POLR3F,POLR3G,POLR3GL,POLR3H,POLR3K,PRKDC,RELA,RIPK1,RIPK3,RPS27A,STAT6,STING1,TBK1,TICAM1,TLR3,TREX1,TRIM21,TRIM32,TRIM56,UBA52,UBB,UBC,XRCC5,XRCC6,ZBP1,
R-HSA-168256	Immune System	8,9b,A1BG,AAAS,AAMP,ABCA13,ABCE1,ABI1,ABI2,ABL1,ABL2,ACAA1,ACLY,ACP3,ACTB,ACTG1,ACTR10,ACTR1A,ACTR1B,ACTR2,ACTR3,ADAM10,ADAM17,ADAM8,ADAR,ADGRE3,ADGRE5,ADGRG3,AGA,AGER,AGL,AGPAT2,AHCYL1,AHSG,AIM2,AIP,AKT1,AKT2,AKT3,ALAD,ALDH3B1,ALDOA,ALDOC,ALOX15,ALOX5,ALPK1,AMPD3,ANAPC1,ANAPC10,ANAPC11,ANAPC13,ANAPC2,ANAPC4,ANAPC5,ANAPC7,ANO6,ANPEP,ANXA1,ANXA2,AOC1,AP1B1,AP1G1,AP1M1,AP1M2,AP1S1,AP1S2,AP1S3,AP2A1
**NFKB1**		
R-HSA-168249	Innate Immune System	9b,A1BG,AAMP,ABCA13,ABI1,ABI2,ABL1,ACAA1,ACLY,ACP3,ACTB,ACTG1,ACTR10,ACTR1B,ACTR2,ACTR3,ADAM10,ADAM8,ADGRE3,ADGRE5,ADGRG3,AGA,AGER,AGL,AGPAT2,AHCYL1,AHSG,AIM2,ALAD,ALDH3B1,ALDOA,ALDOC,ALOX5,ALPK1,AMPD3,ANO6,ANPEP,ANXA2,AOC1,AP1M1,AP2A2,APAF1,APEH,APOB,APP,APRT,ARG1,ARHGAP9,ARL8A,ARMC8,ARPC1A,ARPC1B,ARPC2,ARPC3,ARPC4,ARPC5,ARSA,ARSB,ART1,ASAH1,ATAD3B,ATF1,ATF2,ATG12,ATG5,ATG7,ATOX1,ATP11A
R-HSA-1834949	Cytosolic sensors of pathogen-associated DNA	AIM2,CGAS,CHUK,CRCP,CREBBP,CTNNB1,DDX41,DHX36,DHX9,DTX4,EP300,IFI16,IKBKB,IKBKG,IRF3,IRF7,LRRFIP1,MRE11,MYD88,NFKB1,NFKB2,NFKBIA,NFKBIB,NKIRAS1,NKIRAS2,NLRC3,NLRP4,POLR1C,POLR1D,POLR2E,POLR2F,POLR2H,POLR2K,POLR2L,POLR3A,POLR3B,POLR3C,POLR3D,POLR3E,POLR3F,POLR3G,POLR3GL,POLR3H,POLR3K,PRKDC,RELA,RIPK1,RIPK3,RPS27A,STAT6,STING1,TBK1,TICAM1,TLR3,TREX1,TRIM21,TRIM32,TRIM56,UBA52,UBB,UBC,XRCC5,XRCC6,ZBP1,
R-HSA-168256	Immune System	8,9b,A1BG,AAAS,AAMP,ABCA13,ABCE1,ABI1,ABI2,ABL1,ABL2,ACAA1,ACLY,ACP3,ACTB,ACTG1,ACTR10,ACTR1A,ACTR1B,ACTR2,ACTR3,ADAM10,ADAM17,ADAM8,ADAR,ADGRE3,ADGRE5,ADGRG3,AGA,AGER,AGL,AGPAT2,AHCYL1,AHSG,AIM2,AIP,AKT1,AKT2,AKT3,ALAD,ALDH3B1,ALDOA,ALDOC,ALOX15,ALOX5,ALPK1,AMPD3,ANAPC1,ANAPC10,ANAPC11,ANAPC13,ANAPC2,ANAPC4,ANAPC5,ANAPC7,ANO6,ANPEP,ANXA1,ANXA2,AOC1,AP1B1,AP1G1,AP1M1,AP1M2,AP1S1,AP1S2,AP1S3,AP2A1
R-HSA-4839726	Chromatin organization	ACTB,ACTL6A,ACTL6B,AEBP2,ARID1A,ARID1B,ARID2,ARID4A,ARID4B,ARID5B,ASH1L,ASH2L,ATF2,ATF7IP,ATXN7,ATXN7L3,BRD1,BRD8,BRMS1,BRPF1,BRPF3,BRWD1,CARM1,CCND1,CDK4,CHD3,CHD4,CLOCK,COPRS,CREBBP,DMAP1,DNMT3A,DOT1L,DPY30,DR1,EED,EHMT1,EHMT2,ELP2,ELP3,ELP4,ELP5,ELP6,ENY2,EP300,EP400,EPC1,EZH2,GATAD2A,GATAD2B,GPS2,H2AB1,H2AC1,H2AC11,H2AC12,H2AC14,H2AC18,H2AC20,H2AC21,H2AC4,H2AC6,H2AC7,H2AFX,H2AJ,H2AW,H2AZ1,H2AZ2,H2BC1
R-HSA-3247509	Chromatin modifying enzymes	ACTB,ACTL6A,ACTL6B,AEBP2,ARID1A,ARID1B,ARID2,ARID4A,ARID4B,ARID5B,ASH1L,ASH2L,ATF2,ATF7IP,ATXN7,ATXN7L3,BRD1,BRD8,BRMS1,BRPF1,BRPF3,BRWD1,CARM1,CCND1,CDK4,CHD3,CHD4,CLOCK,COPRS,CREBBP,DMAP1,DNMT3A,DOT1L,DPY30,DR1,EED,EHMT1,EHMT2,ELP2,ELP3,ELP4,ELP5,ELP6,ENY2,EP300,EP400,EPC1,EZH2,GATAD2A,GATAD2B,GPS2,H2AB1,H2AC1,H2AC11,H2AC12,H2AC14,H2AC18,H2AC20,H2AC21,H2AC4,H2AC6,H2AC7,H2AFX,H2AJ,H2AW,H2AZ1,H2AZ2,H2BC1
R-HSA-166016	Toll Like Receptor 4 (TLR4) Cascade	AGER,ALPK1,APP,ATF1,ATF2,BIRC2,BIRC3,BPI,BTK,BTRC,CASP8,CD14,CD180,CD36,CHUK,CREB1,CUL1,DNM1,DNM2,DNM3,DUSP3,DUSP4,DUSP6,DUSP7,ECSIT,ELK1,FADD,FBXW11,FGA,FGB,FGG,FOS,HMGB1,IKBIP,IKBKB,IKBKE,IKBKG,IRAK1,IRAK2,IRAK3,IRAK4,IRF3,IRF7,ITGAM,ITGB2,JUN,LBP,LRRC14,LY86,LY96,MAP2K1,MAP2K3,MAP2K4,MAP2K6,MAP2K7,MAP3K1,MAP3K7,MAP3K8,MAPK1,MAPK10,MAPK11,MAPK14,MAPK3,MAPK7,MAPK8,MAPK9,MAPKAPK2,MAPKAPK3
R-HSA-2871837	FCERI mediated NF-kB activation	BCL10,BTRC,CARD11,CDC34,CHUK,CUL1,FBXW11,IGHE,IGHV,IGHV1-2,IGHV1-46,IGHV1-69,IGHV2-5,IGHV2-70,IGHV3-11,IGHV3-13,IGHV3-23, IGHV3-30,IGHV3-33,IGHV3-48,IGHV3-53,IGHV3-7,IGHV3-9,IGHV4-34, IGHV4-39,IGHV4-59,IGHV7-81,IGKC,IGKV1-12,IGKV1-16,IGKV1-17,IGKV1-33,IGKV1-39,IGKV1-5,IGKV1D-12,IGKV1D-16,IGKV1D-33,IGKV1D-39, IGKV2-28,IGKV2-29,IGKV2-30,IGKV2D-28,IGKV2D-30,IGKV2D-40,IGKV3-11,IGKV3-15,IGKV3-20,IGKV3D-20,IGKV4-1,IGKV5-2, IGLC1, IGLC2, IGLC3,

		IGLC6,IGLC7,IGLV,IGLV1-36,IGLV1-40,IGLV1-44,IGLV1-47,IGLV1-51, IGLV10-54,IGLV11-55,IGLV2-11,IGLV2-14,IGLV2-18,IGLV2-23,IGLV2-33
R-HSA-202403	TCR signaling	BCL10,BTRC,CARD11,CD101,CD247,CD3D,CD3E,CD3G,CD4,CDC34,CHUK,CSK,CUL1,ENAH,EVL,FBXW11,FYB,GRAP2,HLA-DPA1,HLA-DPB1,HLA-DQA1,HLA-DQA2,HLA-DQB1,HLA-DQB2,HLA-DRA,HLA-DRB1,HLA-DRB3,HLA-DRB4,HLA-DRB5, IKBKB, IKBKG, INPP5D, ITK, LAT, LCK, LCP2,MALT1,MAP3K7,NCK1,NFKB1,NFKBIA,PAG1,PAK1,PAK2,PAK3,PDPK1,PIK3CA,PIK3CB,PIK3R1,PIK3R2,PLCG1,PLCG2,PRKCQ,PSMA1,PSMA2,PSMA3,PSMA4,PSMA5,PSMA6,PSMA7,PSMA8,PSMB1,PSMB10,PSMB11,PSMB2,PSMB3,PSMB4,PSMB5
R-HSA-1280218	Adaptive Immune System	8,ACTR10,ACTR1A,ACTR1B,AHCYL1,AKT1,AKT2,AKT3,ANAPC1,ANAPC10,ANAPC11,ANAPC13,ANAPC2,ANAPC4,ANAPC5,ANAPC7,AP1B1,AP1G1,AP1M1,AP1M2,AP1S1,AP1S2,AP1S3,AP2A1,AP2A2,AP2B1,AP2M1,AP2S1,AREL1,ARF1,ARIH2,ASB1,ASB10,ASB11,ASB12,ASB13,ASB14,ASB15,ASB16,ASB17,ASB18,ASB2,ASB3,ASB4,ASB5,ASB6,ASB7,ASB8,ASB9,ATG14,ATG7,B2M,BCL10,BECN1,BLK,BLMH,BLNK,BTBD1,BTBD6,BTK,BTLA,BTN1A1,BTN2A1,BTN2A2,BTN3A1,BTN3A2,BTN3A3,BTNL2
R-HSA-2559583	Cellular Senescence	ACD,AGO1,AGO3,AGO4,ANAPC1,ANAPC10,ANAPC11,ANAPC15,ANAPC16,ANAPC2,ANAPC4,ANAPC5,ANAPC7,ASF1A,ATM,BMI1,CABIN1,CBX2,CBX4,CBX6,CBX8,CCNA1,CCNA2,CCNE1,CCNE2,CDC16,CDC23,CDC26,CDC27,CDK2,CDK4,CDK6,CDKN1A,CDKN1B,CDKN2A,CDKN2B,CDKN2C,CDKN2D,CEBPB,CXCL8,E2F1,E2F2,E2F3,EED,EHMT1,EHMT2,EP400,ERF,ETS1,ETS2,EZH2,FOS,FZR1,H1-0,H1-1,H1-2,H1-3,H1-4,H1-5, H2AB1, H2AC14, H2AC18,H2AC20,H2AC4,H2AC6,H2AC7,H2AFX,H2AJ
R-HSA-1168372	Downstream signaling events of B Cell Receptor (BCR)	BCL10,BTRC,CALM1,CARD11,CHUK,CUL1,FBXW11,FKBP1A,HRAS,IKBKB,IKBKG,KRAS,MALT1,MAP3K7,NFATC1,NFATC2,NFATC3,NFKB1,NFKBIA,NFKBIB,NFKBIE,NRAS,PPIA,PPP3CA,PPP3CB,PPP3R1,PRKCB,PSMA1,PSMA2,PSMA3,PSMA4,PSMA5,PSMA6,PSMA7,PSMA8,PSMB1,PSMB10,PSMB11,PSMB2,PSMB3,PSMB4,PSMB5,PSMB6,PSMB7,PSMB8,PSMB9,PSMC1,PSMC2,PSMC3,PSMC4,PSMC5,PSMC6,PSMD1,PSMD10,PSMD11,PSMD12,PSMD13,PSMD14,PSMD2,PSMD3,PSMD4,PSMD5,PSMD6,PSMD7,PSMD8,PSMD9,PSME1,PSME2
R-HSA-166166	MyD88-independent TLR4 cascade	AGER,ALPK1,APP,ATF1,ATF2,BIRC2,BIRC3,BTRC,CASP8,CD14,CHUK,CREB1,CUL1,DUSP3,DUSP4,DUSP6,DUSP7,ELK1,FADD,FBXW11,FOS,HMGB1,IKBIP,IKBKB,IKBKE,IKBKG,IRAK1,IRAK2,IRF3,IRF7,JUN,LRRC14,LY96,MAP2K1,MAP2K3,MAP2K4,MAP2K6,MAP2K7,MAP3K7,MAP3K8,MAPK1,MAPK10,MAPK11,MAPK14,MAPK3,MAPK7,MAPK8,MAPK9,MAPKAPK2,MAPKAPK3,MEF2A,MEF2C,N,N4BP1,NFKB1,NFKB2,NFKBIA,NFKBIB,NKIRAS1,NKIRAS2,NLRC5,NLRX1,NOD1,NOD2,PPP2CA,PPP2CB,PPP2R1A,PPP2R1B
R-HSA-168181	Toll Like Receptor 7/8 (TLR7/8) Cascade	AGER,ALPK1,APP,ATF1,ATF2,BTRC,CASP8,CD14,CHUK,CREB1,CUL1,DUSP3,DUSP4,DUSP6,DUSP7,ECSIT,ELK1,FBXW11,FOS,HMGB1,IKBIP,IKBKB,IKBKG,IRAK1,IRAK2,IRAK4,IRF7,JUN,LRRC14,LY96,MAP2K1,MAP2K3,MAP2K4,MAP2K6,MAP2K7,MAP3K1,MAP3K7,MAP3K8,MAPK1,MAPK10,MAPK11,MAPK14,MAPK3,MAPK7,MAPK8,MAPK9,MAPKAPK2,MAPKAPK3,MEF2A,MEF2C,MYD88,N,N4BP1,NFKB1,NFKB2,NFKBIA,NFKBIB,NKIRAS1,NKIRAS2,NLRC5,NLRX1,NOD1,NOD2,PELI1,PELI2,PELI3,PPP2CA,PPP2CB
R-HSA-1280215	Cytokine Signaling in Immune system	8,AAAS,ABCE1,ABL2,ADAM17,ADAR,AGER,AIP,AKT1,AKT2,AKT3,ALOX15,ALOX5,ALPK1,ANXA1,ANXA2,APP,ARF1,ARIH1,ATF1,ATF2,B2M,BATF,BCL2,BCL2L1,BCL2L11,BCL6,BIRC2,BIRC3,BIRC5,BLNK,BOLA2,BRWD1,BST2,BTRC,CA1,CAMK2A,CAMK2B,CAMK2D,CAMK2G,CANX,CAPZA1,CASP1,CASP3,CASP8,CBL,CCL11,CCL19,CCL2,CCL20,CCL22,CCL3,CCL3L1,CCL4,CCL5,CCND1,CCR1,CCR2,CCR5,CD27,CD36,CD4,CD40,CD40LG,CD44,CD70,CD80,CD86
R-HSA-5602358	Diseases associated with the TLR signaling cascade	BTK,CD14,CD36,CHUK,FGA,FGB,FGG,HMGB1,IKBKB,IKBKG,IRAK4,LY96,MYD88,NFKB1,NFKB2,NFKBIA,RELA,S100A1,S100A8,S100A9,TICAM1,TIRAP,TLR1,TLR10,TLR2,TLR3,TLR4,TLR5,TLR6,TRAF3,UNC93B1,fliC,mip,porB

R-HSA-450294	MAP kinase activation	ATF1,ATF2,BTRC,CHUK,CREB1,CUL1,DUSP3,DUSP4,DUSP6,DUSP7,ELK1,FBXW11,FOS,IKBKB,IKBKG,IRAK1,IRAK2,JUN,MAP2K1,MAP2K3,MAP2K4,MAP2K6,MAP2K7,MAP3K7,MAP3K8,MAPK1,MAPK10,MAPK11,MAPK14,MAPK3,MAPK7,MAPK8,MAPK9,MAPKAPK2,MAPKAPK3,MEF2A,MEF2C,NFKB1,NOD1,NOD2,PPP2CA,PPP2CB,PPP2R1A,PPP2R1B,PPP2R5D,RIPK2,RPS27A,RPS6KA1,RPS6KA2,RPS6KA3,RPS6KA5,SKP1,TAB1,TAB2,TAB3,TNIP2,TRAF6,UBA52,UBB,UBC,UBE2N,UBE2V1,VRK3
R-HSA-381340	Transcriptional regulation of white adipocyte differentiation	ADIPOQ,ADIRF,ANGPTL4,CARM1,CCNC,CCND3,CD36,CDK19,CDK4,CDK8,CEBPA,CEBPB,CEBPD,CHD9,CREBBP,EBF1,EGR2,EP300,FABP4,FAM120B,HDAC3,HELZ2,KLF4,KLF5,LEP,LPL,MED1,MED10,MED11,MED12,MED13,MED13L,MED14,MED15,MED16,MED17,MED18,MED19,MED20,MED21,MED22,MED23,MED24,MED25,MED26,MED27,MED28,MED29,MED30,MED31,MED4,MED6,MED7,MED8,MED9,NCOA1,NCOA2,NCOA3,NCOA6,NCOR1,NCOR2,NFKB1,NR2F2,PCK1,PLIN1,PPARA,PPARG,PPARGC1A
R-HSA-448424	Interleukin-17 signaling	8,ATF1,ATF2,BTRC,CHUK,CREB1,CUL1,DUSP3,DUSP4,DUSP6,DUSP7,ELK1,FBXW11,FOS,IKBKB,IKBKG,IL17A,IL17C,IL17F,IL17RA,IL17RB,IL17RC,IL17RE,IL25,IRAK1,IRAK2,JUN,MAP2K1,MAP2K3,MAP2K4,MAP2K6,MAP2K7,MAP3K7,MAP3K8,MAPK1,MAPK10,MAPK11,MAPK14,MAPK3,MAPK7,MAPK8,MAPK9,MAPKAPK2,MAPKAPK3,MEF2A,MEF2C,NFKB1,NOD1,NOD2,PPP2CA,PPP2CB,PPP2R1A,PPP2R1B,PPP2R5D,RIPK2,RPS27A,RPS6KA1,RPS6KA2,RPS6KA3,RPS6KA5,SKP1,TAB1,TAB2,TAB3,TNIP2,TRAF6,UBA52,UBB
R-HSA-5260271	Diseases of Immune System	BTK,CD14,CD36,CHUK,FGA,FGB,FGG,HMGB1,IKBKB,IKBKG,IRAK4,LY96,MYD88,NFKB1,NFKB2,NFKBIA,RELA,S100A1,S100A8,S100A9,TICAM1,TIRAP,TLR1,TLR10,TLR2,TLR3,TLR4,TLR5,TLR6,TRAF3,UNC93B1,fliC,mip,porB
R-HSA-9020702	Interleukin-1 signaling	AGER,ALPK1,APP,BTRC,CASP8,CHUK,CUL1,FBXW11,HMGB1,IKBIP,IKBKB,IKBKG,IL1A,IL1B,IL1R1,IL1R2,IL1RAP,IL1RN,IRAK1,IRAK2,IRAK3,IRAK4,LRRC14,MAP2K1,MAP2K4,MAP2K6,MAP3K3,MAP3K7,MAP3K8,MYD88,N,N4BP1,NFKB1,NFKB2,NFKBIA,NFKBIB,NKIRAS1,NKIRAS2,NLRC5,NLRX1,NOD1,NOD2,PELI1,PELI2,PELI3,PSMA1,PSMA2,PSMA3,PSMA4,PSMA5,PSMA6,PSMA7,PSMA8,PSMB1,PSMB10,PSMB11,PSMB2,PSMB3,PSMB4,PSMB5,PSMB6,PSMB7,PSMB8,PSMB9,PSMC1,PSMC2,PSMC3,PSMC4
R-HSA-445989	TAK1-dependent IKK and NF-kappa-B activation	AGER,ALPK1,APP,CASP8,CHUK,HMGB1,IKBIP,IKBKB,IKBKG,IRAK1,IRAK2,LRRC14,MAP3K7,N,N4BP1,NFKB1,NFKB2,NFKBIA,NFKBIB,NKIRAS1,NKIRAS2,NLRC5,NLRX1,NOD1,NOD2,RELA,RIPK2,RPS27A,S100A12,S100B,SAA1,TAB1,TAB2,TAB3,TIFA,TP53,TRAF2,TRAF6,UBA52,UBB,UBC,UBE2N,UBE2V1,USP14,USP18
R-HSA-844456	The NLRP3 inflammasome	APP,CASP1,HMOX1,HSP90AB1,MEFV,NFKB1,NFKB2,NLRP3,P2RX7,PANX1,PSTPIP1,PYCARD,RELA,SUGT1,TXN,TXNIP,hly
R-HSA-1169091	Activation of NF-kappaB in B cells	BCL10,BTRC,CARD11,CHUK,CUL1,FBXW11,IKBKB,IKBKG,MALT1,MAP3K7,NFKB1,NFKBIA,NFKBIB,NFKBIE,PRKCB,PSMA1,PSMA2,PSMA3,PSMA4,PSMA5,PSMA6,PSMA7,PSMA8,PSMB1,PSMB10,PSMB11,PSMB2,PSMB3,PSMB4,PSMB5,PSMB6,PSMB7,PSMB8,PSMB9,PSMC1,PSMC2,PSMC3,PSMC4,PSMC5,PSMC6,PSMD1,PSMD10,PSMD11,PSMD12,PSMD13,PSMD14,PSMD2,PSMD3,PSMD4,PSMD5,PSMD6,PSMD7,PSMD8,PSMD9,PSME1,PSME2,PSME3,PSME4,PSMF1,REL,RELA,RPS27A,SEM1,SKP1,UBA52,UBB,UBC,
R-HSA-168164	Toll Like Receptor 3 (TLR3) Cascade	AGER,ALPK1,APP,ATF1,ATF2,BIRC2,BIRC3,BTRC,CASP8,CHUK,CREB1,CUL1,DUSP3,DUSP4,DUSP6,DUSP7,ELK1,FADD,FBXW11,FOS,HMGB1,IKBIP,IKBKB,IKBKE,IKBKG,IRAK1,IRAK2,IRF3,IRF7,JUN,LRRC14,MAP2K1,MAP2K3,MAP2K4,MAP2K6,MAP2K7,MAP3K7,MAP3K8,MAPK1,MAPK10,MAPK11,MAPK14,MAPK3,MAPK7,MAPK8,MAPK9,MAPKAPK2,MAPKAPK3,MEF2A,MEF2C,N,N4BP1,NFKB1,NFKB2,NFKBIA,NFKBIB,NKIRAS1,NKIRAS2,NLRC5,NLRX1,NOD1,NOD2,PPP2CA,PPP2CB,PPP2R1A,PPP2R1B,PPP2R5D,RELA
R-HSA-168179	Toll Like Receptor TLR1:TLR2 Cascade	AGER,ALPK1,APP,ATF1,ATF2,BTK,BTRC,CASP8,CD14,CD36,CHUK,CREB1,CUL1,DUSP3,DUSP4,DUSP6,DUSP7,ECSIT,ELK1,FBXW11,FGA,FGB,FGG,FOS,HMGB1,IKBIP,IKBKB,IKBKG,IRAK1,IRAK2,IRAK3,IRAK4,JUN,LRRC14,LY96,MAP2K1,MAP2K3,MAP2K4,MAP2K6,MAP2K7,MAP3K1,MAP3K7,MAP3K8,MAPK1,MAPK10,MAPK11,MAPK14,MAPK3,MAPK7,MAPK8,MAPK9,MAPKAPK2,MAPKAPK3,MEF2A,MEF2C,MYD88,N,N4BP1,NFKB1,NFKB2,NFKBIA,NFKBIB,NKIRAS1,NKIRAS2,NLRC5,NLRX1,NOD1,NOD2

R-HSA-5607764	CLEC7A (Dectin-1) signaling	AHCYL1,BCL10,BTRC,CALM1,CARD11,CARD9,CASP8,CCL17,CCL22,CDC34,CHUK,CLEC7A,CUL1,FBXW11,IKBKB,IKBKG,IL1B,ITPR1,ITPR2,ITPR3,MALT1,MAP3K14,MAP3K7,NFATC1,NFATC2,NFATC3,NFKB1,NFKB2,NFKBIA,PDPK1,PLCG2,PPP3CA,PPP3CB,PPP3R1,PRKCD,PSMA1,PSMA2,PSMA3,PSMA4,PSMA5,PSMA6,PSMA7,PSMA8,PSMB1,PSMB10,PSMB11,PSMB2,PSMB3,PSMB4,PSMB5,PSMB6,PSMB7,PSMB8,PSMB9,PSMC1,PSMC2,PSMC3,PSMC4,PSMC5,PSMC6,PSMD1,PSMD10,PSMD11,PSMD12,PSMD13,PSMD14,PSMD2,PSMD3
R-HSA-3134963	DEx/H-box helicases activate type I IFN and inflammatory cytokines production	DHX36,DHX9,IRF7,MYD88,NFKB1,NFKB2,RELA,
R-HSA-5684264	MAP3K8 (TPL2)-dependent MAPK1/3 activation	BTRC,CHUK,CUL1,FBXW11,IKBKB,IKBKG,MAP2K1,MAP2K4,MAP3K8,NFKB1,RPS27A,SKP1,TNIP2,UBA52,UBB,UBC
R-HSA-446652	Interleukin-1 family signaling	AGER,ALOX5,ALPK1,APP,BTRC,CASP1,CASP8,CHUK,CTSG,CUL1,FBXW11,GSDMD,HMGB1,IKBIP,IKBKB,IKBKG,IL13,IL18,IL18BP,IL18R1,IL18RAP,IL1A,IL1B,IL1F10,IL1R1,IL1R2,IL1RAP,IL1RAPL1,IL1RL1,IL1RL2,IL1RN,IL33,IL36A,IL36B,IL36G,IL36RN,IL37,IL4,IRAK1,IRAK2,IRAK3,IRAK4,LRRC14,MAP2K1,MAP2K4,MAP2K6,MAP3K3,MAP3K7,MAP3K8,MAPK8,MYD88,N,N4BP1,NFKB1,NFKB2,NFKBIA,NFKBIB,NKIRAS1,NKIRAS2,NLRC5,NLRX1,NOD1,NOD2,PELI1,PELI2,PELI3,PSMA1,PSMA2
R-HSA-9664424	Cell recruitment (pro-inflammatory response)	C3,C3AR1,CASP1,CTSG,ENTPD1,ENTPD5,GSDMD,HMOX1,HSP90AB1,IL18,IL1A,IL1B,MEFV,NFKB1,NFKB2,NLRP3,NT5E,P2RX4,P2RX7,PSTPIP1,PYCARD,RELA,SUGT1,TXN,TXNIP
R-HSA-933542	TRAF6 mediated NF-kB activation	AGER,APP,CHUK,DDX58,HMGB1,IFIH1,IKBKB,IKBKG,MAP3K1,MAVS,NFKB1,NFKB2,NFKBIA,NFKBIB,NKIRAS1,NKIRAS2,RELA,RNF135,S100A12,S100B,SAA1,TRAF2,TRAF6,TRIM25,TRIM4
R-HSA-168643	Nucleotide-binding domain, leucine rich repeat containing receptor (NLR) signaling pathways	BIRC3,TAB2,CASP8,NFKB2,ITCH,CYLD,P2RX7,NLRC4,NLRP1,HSP90AB1,HMOX1,TAB1,MEFV,PYCARD,RIPK2,IKBKB,NOD1,CASP2,MAP2K6,NFKB1,PANX1,BIRC2,MAPK14,TNFAIP3,AAMP,CASP9,IRAK2,MAP3K7,TXN,CASP1,PSTPIP1,APP,RPS27A,UBC,MAPK13,TAB3,NLRP3,AIM2,SUGT1,NOD2,UBB,BCL2L1,BCL2,RELA,TRAF6,UBE2N,IRAK1,MAPK11,CARD9,MAPK12,CASP4,CHUK,UBA52,UBE2V1,TXNIP,IKBKG
R-HSA-9609646	HCMV Infection	AAAS,CBX1,CEBPD,CHMP1A,CHMP2A,CHMP2B,CHMP3,CHMP4A,CHMP4B,CHMP4C,CHMP6,CHMP7,CREB1,CVC1,CVC2,DAXX,DBP,DUT,DYNC1H1,DYNC1I1,DYNC1I2,DYNC1LI1,DYNC1LI2,DYNLL1,DYNLL2,EED,EGFR,ELK1,EZH2,GPS2,H2AC1,H2AC11,H2AC12,H2AC14,H2AC18,H2AC20,H2AC21,H2AC4,H2AC6,H2AC7,H2AW,H2BC1,H2BC11,H2BC12,H2BC13,H2BC14,H2BC15,H2BC17,H2BC18,H2BC21,H2BC3,H2BC4,H2BC5,H2BC9,H2BU1,H3C1,H3C15,H4C1,HDAC3,HELI,HNRNPK,IRS1,ITGB1,MCP,MVB12A,MVB12B,NCOR1
R-HSA-3214841	PKMTs methylate histone lysines	AEBP2,ASH1L,ASH2L,ATF7IP,DOT1L,DPY30,EED,EHMT1,EHMT2,EZH2,H3C1,H3C15,H4C1,KMT2A,KMT2B,KMT2C,KMT2D,KMT2E,KMT5A,KMT5B,KMT5C,MECOM,NFKB1,NFKB2,NSD1,NSD2,NSD3,PRDM16,PRDM9,RBBP4,RBBP5,RBBP7,RELA,SETD1A,SETD1B,SETD2,SETD3,SETD6,SETD7,SETDB1,SETDB2,SMYD2,SMYD3,SUV39H1,SUV39H2,SUZ12,WDR5
R-HSA-168176	Toll Like Receptor 5 (TLR5) Cascade	AGER,ALPK1,APP,ATF1,ATF2,BTRC,CASP8,CHUK,CREB1,CUL1,DUSP3,DUSP4,DUSP6,DUSP7,ECSIT,ELK1,FBXW11,FOS,HMGB1,IKBIP,IKBKB,IKBKG,IRAK1,IRAK2,IRAK4,JUN,LRRC14,MAP2K1,MAP2K3,MAP2K4,MAP2K6,MAP2K7,MAP3K1,MAP3K7,MAP3K8,MAPK1,MAPK10,MAPK11,MAPK14,MAPK3,MAPK7,MAPK8,MAPK9,MAPKAPK2,MAPKAPK3,MEF2A,MEF2C,MYD88,N,N4BP1,NFKB1,NFKB2,NFKBIA,NFKBIB,NKIRAS1,NKIRAS2,NLRC5,NLRX1,NOD1,NOD2,PELI1,PELI2,PELI3,PPP2CA,PPP2CB,PPP2R1A,PPP2R1B,PPP2R5D
R-HSA-212436	Generic Transcription Pathway	ABCA6,ABL1,ACTL6A,ACTL6B,AGO1,AGO2,AGO3,AGO4,AGRP,AIFM2,AKT1,AKT2,AKT3,ANAPC1,ANAPC10,ANAPC11,ANAPC15,ANAPC16,ANAPC2,ANAPC4,ANAPC5,ANAPC7,APAF1,APOE,AR,ARID1A,ARID1B,ARID2,ARID3A,ASH2L,ATAD2,ATF2,ATM,ATP1B4,ATR,ATRIP,ATXN3,AURKA,AURKB,AUTS2,AXIN1,BANP,BARD1,BAX,BBC3,BCL2L11,BCL2L14,BCL6,BDNF,BGLAP,BID,BIRC5,BLK,BLM,BMI1,BMP2,BNIP3L,BRCA1,BRD1,BRD2,BRD7,BRIP1,BRPF1,BRPF3,BTG1,BTG2,CALM1,CAMK2A
R-HSA-8853884	Transcriptional Regulation by VENTX	AGO1,AGO3,AGO4,ANAPC1,ANAPC10,ANAPC11,ANAPC15,ANAPC16,ANAPC2,ANAPC4,ANAPC5,ANAPC7,CCND1,CDC16,CDC23,CDC26,CDC27,CDKN2A,CEBPB,CSF1R,CTNNB1,EHMT1,EHMT2,FZR1,IL6,LEF1,MIR24-1,MIR24-2, MOV10,

		NFKB1, RELA, TCF7L2, TNRC6A, TNRC6B, TNRC6C, TP53,UBE2C,UBE2D1,UBE2E1,UBE2S,VENTX
R-HSA-975155	MyD88 dependent cascade initiated on endosome	AGER,ALPK1,APP,ATF1,ATF2,BTRC,CASP8,CD14,CHUK,CREB1,CUL1,DUSP3, DUSP4,DUSP6,DUSP7,ECSIT,ELK1,FBXW11,FOS,HMGB1,IKBIP,IKBKB,IKBKG, IRAK1,IRAK2,IRAK4,IRF7,JUN,LRRC14,LY96,MAP2K1,MAP2K3,MAP2K4,MAP2K6,MAP2K7,MAP3K1,MAP3K7,MAP3K8,MAPK1,MAPK10,MAPK11,MAPK14,MAPK3,MAPK7,MAPK8,MAPK9,MAPKAPK2,MAPKAPK3,MEF2A,MEF2C,MYD88,N,N4BP1,NFKB1,NFKB2,NFKBIA,NFKBIB,NKIRAS1,NKIRAS2,NLRC5,NLRX1,NOD1,NOD2,PELI1,PELI2,PELI3,PPP2CA,PPP2CB
R-HSA-448706	Interleukin-1 processing	CASP1,CTSG,GSDMD,IL18,IL1A,IL1B,NFKB1,NFKB2,RELA,
R-HSA-983705	Signaling by the B Cell Receptor (BCR)	AHCYL1,BCL10,BLK,BLNK,BTK,BTRC,CALM1,CARD11,CD19,CD22,CD79A,CD79B,CHUK,CUL1,DAPP1,FBXW11,FKBP1A,FYN,GRB2,HRAS,IGHD,IGHM,IGHV,IGHV1-2,IGHV1-46,IGHV1-69,IGHV2-5,IGHV2-70,IGHV3-11, IGHV3-13,IGHV3-23,IGHV3-30,IGHV3-33,IGHV3-48,IGHV3-53,IGHV3-7, IGHV3-9,IGHV4-34,IGHV4-39,IGHV4-59,IGHV7-81,IGKC,IGKV1-12,IGKV1-16, IGKV1-17, IGKV1-33, IGKV1-39, IGKV1-5, IGKV1D-12, IGKV1D-16, IGKV1D-33,IGKV1D-39,IGKV2-28,IGKV2-29,IGKV2-30,IGKV2D-28,IGKV2D-30,IGKV2D-40,IGKV3-11,IGKV3-15,IGKV3-20,IGKV3D-20,IGKV4-1,IGKV5-2,IGLC1,IGLC2,IGLC3,IGLC6
R-HSA-975138	TRAF6 mediated induction of NFkB and MAP kinases upon TLR7/8 or 9 activation	AGER,ALPK1,APP,ATF1,ATF2,BTRC,CASP8,CD14,CHUK,CREB1,CUL1,DUSP3, DUSP4,DUSP6,DUSP7,ECSIT,ELK1,FBXW11,FOS,HMGB1,IKBIP,IKBKB,IKBKG, IRAK1,IRAK2,IRAK4,JUN,LRRC14,LY96,MAP2K1,MAP2K3,MAP2K4,MAP2K6, MAP2K7,MAP3K1,MAP3K7,MAP3K8,MAPK1,MAPK10,MAPK11,MAPK14,MAPK3,MAPK7,MAPK8,MAPK9,MAPKAPK2,MAPKAPK3,MEF2A,MEF2C,MYD88,N,N4BP1,NFKB1,NFKB2,NFKBIA,NFKBIB,NKIRAS1,NKIRAS2,NLRC5,NLRX1,NOD1,NOD2,PELI1,PELI2,PELI3,PPP2CA,PPP2CB,PPP2R1A
R-HSA-8953897	Cellular responses to stimuli	18S rRNA, 28S rRNA, 5.8S rRNA, 5S rRNA, AAAS, ABCC1, ACADVL, ACD, ACTR10,ACTR1A,ADD1,AGO1,AGO3,AGO4,AJUBA,AKT1,AKT1S1,AKT2,AKT3, ALB,ANAPC1,ANAPC10,ANAPC11,ANAPC15,ANAPC16,ANAPC2,ANAPC4,ANAPC5,ANAPC7,APOA1,APOB,AQP8,AR,ARFGAP1,ARNT,ARNTL,ASF1A,ASNS,ATF2,ATF3,ATF4,ATF5,ATF6,ATM,ATOX1,ATP6V0B,ATP6V0C,ATP6V0D1,ATP6V0D2,ATP6V0E1,ATP6V0E2,ATP6V1A,ATP6V1B1,ATP6V1B2,ATP6V1C1,ATP6V1C2,ATP6V1D,ATP6V1E1,ATP6V1E2,ATP6V1F,ATP6V1G1,ATP6V1G2,ATP6V1G3,ATP6V1H,ATP7A,ATPV0D1,ATR,BACH1
R-HSA-74160	Gene expression (Transcription)	18S rRNA, 5.8S rRNA, 28S rRNA,28S rRNA,45S pre-rRNA gene,5.8S rRNA,5S rRNA,AAAS,ABCA6,ABL1,ACTB,ACTL6A,ACTL6B,AEBP2,AFF4,AGO1,AGO2,AGO3,AGO4,AGRP,AIFM2,AKT1,AKT2,AKT3,ALYREF,ANAPC1,ANAPC10,ANAPC11,ANAPC15,ANAPC16,ANAPC2,ANAPC4,ANAPC5,ANAPC7,ANG,APAF1,APOE,AR,ARID1A,ARID1B,ARID2,ARID3A,ARID4B,ASH2L,ASUN,ASZ1,ATAD2,ATF2,ATM,ATP1B4,ATR,ATRIP,ATXN3,AURKA,AURKB,AUTS2,AXIN1,BANP,BARD1,BAX,BAZ1B,BAZ2A,BBC3,BCDIN3D,BCL2L11,BCL2L14,BCL6,BDNF
R-HSA-622312	Inflammasomes	AIM2,APP,BCL2,BCL2L1,CASP1,HMOX1,HSP90AB1,MEFV,NFKB1,NFKB2,NLRC4,NLRP1,NLRP3,P2RX7,PANX1,PSTPIP1,PYCARD,RELA,SUGT1,TXN,TXNIP,fliC,fljB,hly,prgJ
R-HSA-2559582	Senescence-Associated Secretory Phenotype (SASP)	ANAPC1,ANAPC10,ANAPC11,ANAPC15,ANAPC16,ANAPC2,ANAPC4,ANAPC5, ANAPC7,CCNA1,CCNA2,CDC16,CDC23,CDC26,CDC27,CDK2,CDK4,CDK6,CDKN1A,CDKN1B,CDKN2A,CDKN2B,CDKN2C,CDKN2D,CEBPB,CXCL8,EHMT1,EHMT2,FOS,FZR1,H2AB1,H2AC14,H2AC18,H2AC20,H2AC4,H2AC6,H2AC7,H2AFX, H2AJ,H2AZ1,H2AZ2,H2BC1,H2BC11,H2BC12,H2BC12L,H2BC13,H2BC14,H2BC15,H2BC17,H2BC21,H2BC3,H2BC4,H2BC5,H2BC9,H2BU1,H3-3A, H3C1, H3C15, H4C1, IGFBP7, IL1A, IL6, IL8, JUN, MAPK1, MAPK3, MAPK7,NFKB1
R-HSA-975871	MyD88 cascade initiated on plasma membrane	AGER,ALPK1,APP,ATF1,ATF2,BTRC,CASP8,CHUK,CREB1,CUL1,DUSP3,DUSP4,DUSP6,DUSP7,ECSIT,ELK1,FBXW11,FOS,HMGB1,IKBIP,IKBKB,IKBKG,IRAK1, IRAK2,IRAK4,JUN,LRRC14,MAP2K1,MAP2K3,MAP2K4,MAP2K6,MAP2K7,MAP3K1,MAP3K7,MAP3K8,MAPK1,MAPK10,MAPK11,MAPK14,MAPK3,MAPK7,MAPK8,MAPK9,MAPKAPK2,MAPKAPK3,MEF2A,MEF2C,MYD88,N,N4BP1,NFKB1, NFKB2,NFKBIA,NFKBIB,NKIRAS1,NKIRAS2,NLRC5,NLRX1,NOD1,NOD2,PELI1,PELI2,PELI3,PPP2CA,PPP2CB,PPP2R1A,PPP2R1B,PPP2R5D

R-HSA-6798695	Neutrophil degranulation	A1BG,ABCA13,ACAA1,ACLY,ACP3,ACTR10,ACTR1B,ACTR2,ADAM10,ADAM8,ADGRE3,ADGRE5,ADGRG3,AGA,AGL,AGPAT2,AHSG,ALAD,ALDH3B1,ALDOA,ALDOC,ALOX5,AMPD3,ANO6,ANPEP,ANXA2,AOC1,AP1M1,AP2A2,APAF1,APEH,APRT,ARG1,ARHGAP9,ARL8A,ARMC8,ARPC5,ARSA,ARSB,ASAH1,ATAD3B,ATG7,ATP11A,ATP11B,ATP6AP2,ATP6V0A1,ATP6V0C,ATP6V1D,ATP8A1,ATP8B4,AZU1,B2M,B4GALT1,BIN2,BPI,BRI3,BST1,BST2,C3,C3AR1,C5AR1,C6orf120,CAB39,CALML5,CAMP,CAND1,CANT1,CAP1
R-HSA-5603029	IkBA variant leads to EDA-ID	CHUK,IKBKB,IKBKG,NFKB1,NFKB2,NFKBIA,RELA,
R-HSA-9609690	HCMV Early Events	AAAS,CBX1,CREB1,CVC1,CVC2,DAXX,DBP,DUT,DYNC1H1,DYNC1I1,DYNC1I2,DYNC1LI1,DYNC1LI2,DYNLL1,DYNLL2,EED,EGFR,ELK1,EZH2,GPS2,H2AC1,H2AC11,H2AC12,H2AC14,H2AC18,H2AC20,H2AC21,H2AC4,H2AC6,H2AC7,H2AW,H2BC1,H2BC11,H2BC12,H2BC13,H2BC14,H2BC15,H2BC17,H2BC18,H2BC21,H2BC3,H2BC4,H2BC5,H2BC9,H2BU1,H3C1,H3C15,H4C1,HDAC3,HELI,IRS1,ITGB1,MCP,NCOR1,NCOR2,NDC1,NEC1,NEC2,NFKB1,NUP107,NUP133,NUP153,NUP155,NUP160,NUP188,NUP205,NUP210
R-HSA-202424	Downstream TCR signaling	BCL10,BTRC,CARD11,CD247,CD3D,CD3E,CD3G,CD4,CDC34,CHUK,CUL1,FBXW11,HLA-DPA1,HLA-DPB1,HLA-DQA1,HLA-DQA2,HLA-DQB1,HLA-DQB2,HLA-DRA,HLA-DRB1,HLA-DRB3,HLA-DRB4,HLA-DRB5, IKBKB, IKBKG,INPP5D,LCK,MALT1,MAP3K7,NFKB1,NFKBIA,PDPK1,PIK3CA,PIK3CB,PIK3R1,PIK3R2,PRKCQ,PSMA1,PSMA2,PSMA3,PSMA4,PSMA5,PSMA6,PSMA7,PSMA8,PSMB1,PSMB10,PSMB11,PSMB2,PSMB3,PSMB4,PSMB5,PSMB6,PSMB7,PSMB8,PSMB9,PSMC1,PSMC2,PSMC3,PSMC4,PSMC5,PSMC6,PSMD1,PSMD10,PSMD11,PSMD12,PSMD13,PSMD14
R-HSA-193692	Regulated proteolysis of p75NTR	ADAM17,APH1A,APH1B,NCSTN,NFKB1,NGFR,PSEN1,PSEN2,PSENEN,RELA,TRAF6,,
R-HSA-5663205	Infectious disease	18S rRNA, 28S rRNA,5.8S rRNA, 5S rRNA, 7SL RNA, AAAS, ABI1, ABI2, ABL1, ACE2,ACTB,ACTG1,ACTR2,ACTR3,ADAM17,ADCY1,ADCY2,ADCY3,ADCY4,ADCY5,ADCY6,ADCY7,ADCY8,ADCY9,ADCYAP1,ADCYAP1R1,ADM,ADM2,ADORA2A,ADORA2B,ADRB1,ADRB2,ADRB3,AGRN,AHCYL1,AKT1,AKT2,AKT3,ANTXR1,ANTXR2,AP1B1,AP1G1,AP1M1,AP1M2,AP1S1,AP1S2,AP1S3,AP2A1,AP2A2,AP2B1,AP2M1,AP2S1,APOBEC3G,ARF1,ARID4A,ARID4B,ARPC1A,ARPC1B,ARPC2,ARPC3
R-HSA-181438	Toll Like Receptor 2 (TLR2) Cascade	AGER,ALPK1,APP,ATF1,ATF2,BTK,BTRC,CASP8,CD14,CD36,CHUK,CREB1,CUL1,DUSP3,DUSP4,DUSP6,DUSP7,ECSIT,ELK1,FBXW11,FGA,FGB,FGG,FOS,HMGB1,IKBIP,IKBKB,IKBKG,IRAK1,IRAK2,IRAK3,IRAK4,JUN,LRRC14,LY96,MAP2K1,MAP2K3,MAP2K4,MAP2K6,MAP2K7,MAP3K1,MAP3K7,MAP3K8,MAPK1,MAPK10,MAPK11,MAPK14,MAPK3,MAPK7,MAPK8,MAPK9,MAPKAPK2,MAPKAPK3,MEF2A,MEF2C,MYD88,N,N4BP1,NFKB1,NFKB2,NFKBIA,NFKBIB,NKIRAS1,NKIRAS2,NLRC5,NLRX1,NOD1,NOD2
R-HSA-5660668	CLEC7A/inflammasome pathway	CASP8,IL1B,MALT1,NFKB1,PYCARD,RELA,,
R-HSA-73887	Death Receptor Signalling	AATF,ABR,ADAM17,AKAP13,APH1A,APH1B,ARHGDIA,ARHGEF1,ARHGEF10,ARHGEF10L,ARHGEF11,ARHGEF12,ARHGEF15,ARHGEF16,ARHGEF17,ARHGEF18,ARHGEF19,ARHGEF2,ARHGEF26,ARHGEF3,ARHGEF33,ARHGEF35,ARHGEF37,ARHGEF38,ARHGEF39,ARHGEF4,ARHGEF40,ARHGEF5,ARHGEF6,ARHGEF7,ARHGEF9,BAD,BAG4,BCL2L11,BEX3,BIRC2,BIRC3,CASP10,CASP2,CASP3,CASP8,CFLAR,CHUK,CLIP3,CRMA,CYLD,ECT2,FADD,FAS,FASLG,FGD1,FGD2,FGD3,FGD4,GNA13,HDAC1,HDAC2,HDAC3,IKBKB,IKBKG,IRAK1,ITGB3BP,ITSN1,KALRN,LINGO1,MADD,MAG,MAGED1
R-HSA-9660826	Purinergic signaling in leishmaniasis infection	C3,C3AR1,CASP1,CTSG,ENTPD1,ENTPD5,GSDMD,HMOX1,HSP90AB1,IL18,IL1A,IL1B,MEFV,NFKB1,NFKB2,NLRP3,NT5E,P2RX4,P2RX7,PSTPIP1,PYCARD,RELA,SUGT1,TXN,TXNIP
R-HSA-168138	Toll Like Receptor 9 (TLR9) Cascade	AGER,ALPK1,APP,ATF1,ATF2,BTRC,CASP8,CD14,CHUK,CREB1,CUL1,DUSP3,DUSP4,DUSP6,DUSP7,ECSIT,EEA1,ELK1,FBXW11,FOS,HMGB1,IKBIP,IKBKB,IKBKG,IRAK1,IRAK2,IRAK4,IRF7,JUN,LRRC14,LY96,MAP2K1,MAP2K3,MAP2K4,MAP2K6,MAP2K7,MAP3K1,MAP3K7,MAP3K8,MAPK1,MAPK10,MAPK11,MAPK14,MAPK3,MAPK7,MAPK8,MAPK9,MAPKAPK2,MAPKAPK3,MEF2A,MEF2C,MYD88,N,N4BP1,NFKB1,NFKB2,NFKBIA,NFKBIB,NKIRAS1,NKIRAS2,NLRC5,NLRX1,NOD1,NOD2,PELI1,PELI2,PELI3,PIK3C3

R-HSA-449147	Signaling by Interleukins	8,AGER,AIP,AKT1,ALOX15,ALOX5,ALPK1,ANXA1,ANXA2,APP,ARF1,ATF1,ATF2,BATF,BCL2,BCL2L1,BCL6,BIRC5,BLNK,BOLA2,BRWD1,BTRC,CA1,CANX,CAPZA1,CASP1,CASP3,CASP8,CBL,CCL11,CCL19,CCL2,CCL20,CCL22,CCL3,CCL3L1,CCL4,CCL5,CCND1,CCR1,CCR2,CCR5,CD36,CD4,CD80,CD86,CDC42,CDKN1A,CEBPD,CFL1,CHUK,CISH,CLCF1,CNN2,CNTF,CNTFR,COL1A2,CREB1,CRK,CRKL,CRLF1,CRLF2,CSF1,CSF1R,CSF2,CSF2 gene,CSF2RA,CSF2RB
R-HSA-9658195	Leishmania infection	ABI1,ABI2,ABL1,ACTB,ACTG1,ACTR2,ACTR3,ADAM17,ADCY1,ADCY2,ADCY3,ADCY4,ADCY5,ADCY6,ADCY7,ADCY8,ADCY9,ADCYAP1,ADCYAP1R1,ADM,ADM2,ADORA2A,ADORA2B,ADRB1,ADRB2,ADRB3,AHCYL1,ARPC1A,ARPC1B,ARPC2,ARPC3,ARPC4,ARPC5,AVP,AVPR2,BAIAP2,BRK1,BTK,C3,C3AR1,CALCA,CALCB,CALCR,CALCRL,CALM1,CASP1,CD163,CD247,CD3G,CDC42,CGA,CREB1,CRH,CRHR1,CRHR2,CRK,CTSG,CYBA,CYFIP1,CYFIP2,CYSLTR1,CYSLTR2,DOCK1,DPEP1,DPEP2,DPEP3,DRD1,DRD5
R-HSA-73857	RNA Polymerase II Transcription	ABCA6,ABL1,ACTL6A,ACTL6B,AFF4,AGO1,AGO2,AGO3,AGO4,AGRP,AIFM2,AKT1,AKT2,AKT3,ALYREF,ANAPC1,ANAPC10,ANAPC11,ANAPC15,ANAPC16,ANAPC2,ANAPC4,ANAPC5,ANAPC7,APAF1,APOE,AR,ARID1A,ARID1B,ARID2,ARID3A,ASH2L,ASUN,ATAD2,ATF2,ATM,ATP1B4,ATR,ATRIP,ATXN3,AURKA,AURKB,AUTS2,AXIN1,BANP,BARD1,BAX,BBC3,BCL2L11,BCL2L14,BCL6,BDNF,BGLAP,BID,BIRC5,BLK,BLM,BMI1,BMP2,BNIP3L,BRCA1,BRD1,BRD2,BRD7,BRIP1,BRPF1,BRPF3,BTG1
R-HSA-2454202	Fc epsilon receptor (FCERI) signaling	AHCYL1,BCL10,BTK,BTRC,CALM1,CARD11,CDC34,CHUK,CUL1,FBXW11,FCER1A,FCER1G,FOS,FYN,GAB2,GRAP2,GRB2,HRAS,IGHE,IGHV,IGHV1-2, IGHV1-46,IGHV1-69,IGHV2-5,IGHV2-70,IGHV3-11,IGHV3-13,IGHV3-23, IGHV3-30,IGHV3-33,IGHV3-48,IGHV3-53,IGHV3-7,IGHV3-9,IGHV4-34, IGHV4-39,IGHV4-59,IGHV7-81,IGKC,IGKV1-12,IGKV1-16,IGKV1-17,IGKV1-33,IGKV1-39,IGKV1-5,IGKV1D-12,IGKV1D-16,IGKV1D-33,IGKV1D-39, IGKV2-28,IGKV2-29,IGKV2-30,IGKV2D-28,IGKV2D-30,IGKV2D-40,IGKV3-11,IGKV3-15,IGKV3-20,IGKV3D-20,IGKV4-1,IGKV5-2, IGLC1, IGLC2, IGLC3, IGLC6,IGLC7,IGLV,IGLV1-36
R-HSA-5621575	CD209 (DC-SIGN) signaling	CD209,CREBBP,EP300,FYN,HRAS,ICAM2,ICAM3,KRAS,LYN,NFKB1,NRAS,PAK1,PAK2,PAK3,PRKACA,PRKACB,PRKACG,RAF1,RELA,RELB,RPS6KA5
R-HSA-1606322	ZBP1(DAI) mediated induction of type I IFNs	CHUK,DHX9,DTX4,IKBKB,IKBKG,IRF3,MYD88,NFKB1,NFKB2,NFKBIA,NFKBIB,NKIRAS1,NKIRAS2,NLRP4,RELA,RIPK1,RIPK3,TBK1,TICAM1,TLR3,ZBP1
R-HSA-168898	Toll-like Receptor Cascades	AGER,ALPK1,APOB,APP,ATF1,ATF2,BIRC2,BIRC3,BPI,BTK,BTRC,CASP8,CD14,CD180,CD36,CHUK,CNPY3,CREB1,CTSB,CTSK,CTSL,CTSS,CTSV,CUL1,DNM1,DNM2,DNM3,DUSP3,DUSP4,DUSP6,DUSP7,ECSIT,EEA1,ELK1,FADD,FBXW11,FGA,FGB,FGG,FOS,GSDMD,GSDME,HMGB1,HSP90B1,IKBIP,IKBKB,IKBKE,IKBKG,IRAK1,IRAK2,IRAK3,IRAK4,IRF3,IRF7,ITGAM,ITGB2,JUN,LBP,LGMN,LRRC14,LY86,LY96,MAP2K1,MAP2K3,MAP2K4,MAP2K6,MAP2K7,MAP3K1
R-HSA-193639	p75NTR signals via NF-kB	IKBKB,IRAK1,MYD88,NFKB1,NFKBIA,NGF,NGFR,PRKCI,RELA,RIPK2,RPS27A,SQSTM1,TRAF6,UBA52,UBB,UBC
R-HSA-1266738	Developmental Biology	18S rRNA, 28S rRNA, 5.8S rRNA, 5S rRNA, ABL1, ABL2, ABLIM1, ABLIM2, ABLIM3,ACTB,ACTG1,ACTR2,ACTR3,ACVR1B,ACVR1C,ACVR2A,ACVR2B,ADAM10,ADAM11,ADAM22,ADAM23,ADGRG6,ADGRV1,ADIPOQ,ADIRF,AGAP2,AGRN,AJUBA,AKAP5,AKT1,AKT2,AKT3,ALCAM,AMH,ANGPTL4,ANK1,ANK2,ANK3,AP2A1,AP2A2,AP2B1,AP2M1,AP2S1,APH1A,APH1B,ARHGAP35,ARHGAP39,ARHGEF11,ARHGEF12,ARHGEF28,ARHGEF7,ARPC1A,ARPC1B,ARPC2,ARPC3,ARPC4,ARPC5,ARTN,ASH2L,BMP4,BNIP2,BOC,CACNA1C,CACNA1D,CACNA1G,CACNA1H,CACNA1I,CACNA1S
R-HSA-937061	TRIF(TICAM1)-mediated TLR4 signaling	AGER,ALPK1,APP,ATF1,ATF2,BIRC2,BIRC3,BTRC,CASP8,CD14,CHUK,CREB1,CUL1,DUSP3,DUSP4,DUSP6,DUSP7,ELK1,FADD,FBXW11,FOS,HMGB1,IKBIP,IKBKB,IKBKE,IKBKG,IRAK1,IRAK2,IRF3,IRF7,JUN,LRRC14,LY96,MAP2K1,MAP2K3,MAP2K4,MAP2K6,MAP2K7,MAP3K7,MAP3K8,MAPK1,MAPK10,MAPK11,MAPK14,MAPK3,MAPK7,MAPK8,MAPK9,MAPKAPK2,MAPKAPK3,MEF2A,MEF2C,N,N4BP1,NFKB1,NFKB2,NFKBIA,NFKBIB,NKIRAS1,NKIRAS2,NLRC5,NLRX1,NOD1,NOD2,PPP2CA,PPP2CB,PPP2R1A,PPP2R1B
R-HSA-2262752	Cellular responses to stress	18S rRNA, 28S rRNA, 5.8S rRNA, 5S rRNA, AAAS, ABCC1, ACADVL, ACD,ACTR10,ACTR1A,ADD1,AGO1,AGO3,AGO4,AJUBA,AKT1,AKT1S1,AKT2,AKT3,ALB,ANAPC1,ANAPC10,ANAPC11,ANAPC15,ANAPC16,ANAPC2,ANAPC4,ANAPC5,ANAPC7,APOA1,APOB,AQP8,AR,ARFGAP1,ARNT,ARNTL,ASF1A,ASNS,ATF2,ATF3,ATF4,ATF5,ATF6,ATM,ATOX1,ATP6V0B,ATP6V0C,ATP6V0D1,ATP6V0D2,ATP6V0E1,ATP6V0E2,ATP6V1A,ATP6V1B1,ATP6V1B2,ATP6V1C1,A

		TP6V1C2,ATP6V1D,ATP6V1E1,ATP6V1E2,ATP6V1F,ATP6V1G1,ATP6V1G2,ATP6V1G3,ATP6V1H,ATP7A,ATPV0D1,ATR,BACH1
R-HSA-168142	Toll Like Receptor 10 (TLR10) Cascade	AGER,ALPK1,APP,ATF1,ATF2,BTRC,CASP8,CHUK,CREB1,CUL1,DUSP3,DUSP4,DUSP6,DUSP7,ECSIT,ELK1,FBXW11,FOS,HMGB1,IKBIP,IKBKB,IKBKG,IRAK1,IRAK2,IRAK4,JUN,LRRC14,MAP2K1,MAP2K3,MAP2K4,MAP2K6,MAP2K7,MAP3K1,MAP3K7,MAP3K8,MAPK1,MAPK10,MAPK11,MAPK14,MAPK3,MAPK7,MAPK8,MAPK9,MAPKAPK2,MAPKAPK3,MEF2A,MEF2C,MYD88,N,N4BP1,NFKB1,NFKB2,NFKBIA,NFKBIB,NKIRAS1,NKIRAS2,NLRC5,NLRX1,NOD1,NOD2,PELI1,PELI2,PELI3,PPP2CA,PPP2CB,PPP2R1A,PPP2R1B,PPP2R5D
R-HSA-209560	NF-kB is activated and signals survival	IKBKB,IRAK1,NFKB1,NFKBIA,NGF,NGFR,RELA,RPS27A,SQSTM1,TRAF6,UBA52,UBB,UBC
R-HSA-168188	Toll Like Receptor TLR6:TLR2 Cascade	AGER,ALPK1,APP,ATF1,ATF2,BTK,BTRC,CASP8,CD14,CD36,CHUK,CREB1,CUL1,DUSP3,DUSP4,DUSP6,DUSP7,ECSIT,ELK1,FBXW11,FGA,FGB,FGG,FOS,HMGB1,IKBIP,IKBKB,IKBKG,IRAK1,IRAK2,IRAK3,IRAK4,JUN,LRRC14,LY96,MAP2K1,MAP2K3,MAP2K4,MAP2K6,MAP2K7,MAP3K1,MAP3K7,MAP3K8,MAPK1,MAPK10,MAPK11,MAPK14,MAPK3,MAPK7,MAPK8,MAPK9,MAPKAPK2,MAPKAPK3,MEF2A,MEF2C,MYD88,N,N4BP1,NFKB1,NFKB2,NFKBIA,NFKBIB,NKIRAS1,NKIRAS2,NLRC5,NLRX1,NOD1,NOD2
R-HSA-193704	p75 NTR receptor-mediated signalling	AATF,ABR,ADAM17,AKAP13,APH1A,APH1B,ARHGDIA,ARHGEF1,ARHGEF10,ARHGEF10L,ARHGEF11,ARHGEF12,ARHGEF15,ARHGEF16,ARHGEF17,ARHGEF18,ARHGEF19,ARHGEF2,ARHGEF26,ARHGEF3,ARHGEF33,ARHGEF35,ARHGEF37,ARHGEF38,ARHGEF39,ARHGEF4,ARHGEF40,ARHGEF5,ARHGEF6,ARHGEF7,ARHGEF9,BAD,BCL2L11,BEX3,CASP2,CASP3,ECT2,FGD1,FGD2,FGD3,FGD4,GNA13,HDAC1,HDAC2,HDAC3,IKBKB,IRAK1,ITGB3BP,ITSN1,KALRN,LINGO1,MAG,MAGED1,MAPK8,MCF2,MCF2L,MYD88,NCSTN,NET1,NFKB1,NFKBIA,NGEF,NGF,NGFR,OBSCN,OMG,PLEKHG2,PLEKHG5
R-HSA-166058	MyD88:MAL(TIRAP) cascade initiated on plasma membrane	AGER,ALPK1,APP,ATF1,ATF2,BTK,BTRC,CASP8,CD14,CD36,CHUK,CREB1,CUL1,DUSP3,DUSP4,DUSP6,DUSP7,ECSIT,ELK1,FBXW11,FGA,FGB,FGG,FOS,HMGB1,IKBIP,IKBKB,IKBKG,IRAK1,IRAK2,IRAK3,IRAK4,JUN,LRRC14,LY96,MAP2K1,MAP2K3,MAP2K4,MAP2K6,MAP2K7,MAP3K1,MAP3K7,MAP3K8,MAPK1,MAPK10,MAPK11,MAPK14,MAPK3,MAPK7,MAPK8,MAPK9,MAPKAPK2,MAPKAPK3,MEF2A,MEF2C,MYD88,N,N4BP1,NFKB1,NFKB2,NFKBIA,NFKBIB,NKIRAS1,NKIRAS2,NLRC5,NLRX1,NOD1,NOD2
R-HSA-168928	DDX58/IFIH1-mediated induction of interferon-alpha/beta	9b,AGER,APP,ATG12,ATG5,CASP10,CASP8,CHUK,CREBBP,CYLD,DDX58,DHX58,EP300,FADD,HERC5,HMGB1,HSP90AA1,HSP90AB1,IFIH1,IFNA1,IFNA10,IFNA14,IFNA16,IFNA17,IFNA2,IFNA21,IFNA4,IFNA5,IFNA6,IFNA7,IFNA8,IFNB1,IKBKB,IKBKE,IKBKG,IRF3,IRF7,ISG15,ITCH,M,MAP3K1,MAVS,N,NFKB1,NFKB2,NFKBIA,NFKBIB,NKIRAS1,NKIRAS2,NLRC5,NLRX1,OTUD5,PCBP2,PIN1,RELA,RIPK1,RNF125,RNF135,RNF216,RPS27A,S100A12,S100B,SAA1,SIKE1,TANK,TAX1BP1,TBK1,TKFC
R-HSA-1643685	Disease	18S rRNA, 28S rRNA, 5.8S rRNA,5S rRNA,7SL RNA,AAAS,ABCA1,ABCA12,ABCA3,ABCA4,ABCB11,ABCB4,ABCB6,ABCC2,ABCC6,ABCC8,ABCC9,ABCD1,ABCD4,ABCG5,ABCG8,ABI1,ABI2,ABL1,ACACA,ACAN,ACE2,ACTB,ACTG1,ACTR2,ACTR3,ACY1,ADA,ADAM10,ADAM17,ADAMTS1,ADAMTS10,ADAMTS12,ADAMTS13,ADAMTS14,ADAMTS15,ADAMTS16,ADAMTS17,ADAMTS18,ADAMTS19,ADAMTS2,ADAMTS20,ADAMTS3,ADAMTS4,ADAMTS5,ADAMTS6,ADAMTS7,ADAMTS8,ADAMTS9,ADAMTSL1,ADAMTSL2,ADAMTSL3,ADAMTSL4,ADAMTSL5,ADCY1
R-HSA-5621481	C-type lectin receptors (CLRs)	AHCYL1,BCL10,BTRC,CALM1,CARD11,CARD9,CASP8,CCL17,CCL22,CD209,CDC34,CHUK,CLEC10A,CLEC4A,CLEC4C,CLEC4D,CLEC4E,CLEC6A,CLEC7A,CREBBP,CUL1,EP300,FBXW11,FCER1G,FYN,HRAS,ICAM2,ICAM3,IKBKB,IKBKG,IL1B,ITPR1,ITPR2,ITPR3,KRAS,LYN,MALT1,MAP3K14,MAP3K7,MUC1,MUC12,MUC13,MUC15,MUC16,MUC17,MUC19,MUC2,MUC20,MUC21,MUC3A,MUC3B,MUC4,MUC5AC,MUC5B,MUC6,MUC7,MUCL1,NFATC1,NFATC2,NFATC3,NFKB1,NFKB2,NFKBIA,NRAS,PAK1,PAK2,PAK3,PDPK1

R-HSA-162582	Signal Transduction	AAAS,AAMP,AATF,ABCA1,ABCD3,ABCG1,ABCG5,ABCG8,ABHD12,ABHD17A,ABHD17B,ABHD17C,ABHD6,ABI1,ABI2,ABL1,ABL2,ABR,ACBD5,ACKR1,ACKR2,ACKR3,ACKR4,ACTA2,ACTB,ACTC1,ACTG1,ACTN1,ACTN2,ACTR2,ACTR3,ACVR1B,ACVR1C,ACVR2A,ACVR2B,ACVRL1,ADAM10,ADAM12,ADAM17,ADAP1,ADCY1,ADCY10,ADCY2,ADCY3,ADCY4,ADCY5,ADCY6,ADCY7,ADCY8,ADCY9,ADCYAP1,ADCYAP1R1,ADD3,ADGRE1,ADGRE2,ADGRE3,ADGRE5,ADH1A,ADH1C,ADH4,ADM,ADM2,ADORA1,ADORA2A,ADORA2B,ADORA3,ADRA1A,ADRA1B
R-HSA-1810476	RIP-mediated NFkB activation via ZBP1	CHUK,DHX9,IKBKB,IKBKG,MYD88,NFKB1,NFKB2,NFKBIA,NFKBIB,NKIRAS1,NKIRAS2,RELA,RIPK1,RIPK3,TICAM1,TLR3,ZBP1

Supplementary table S6. Immune cell sub-types abundance captured for 64 different cell types.

**Celltype distribution**	**IGIB11301042671V**	**IGIB1130797709V**	**IGIB1130540315V**	**IGIB1130538756V**	**IGIB1130536690V**	**IGIB1130534678V**	**IGIB1130534086V**	**IGIB1130532747V**	**IGIB1130527553V**	**IGIB1130519238V**	**IGIB1130519233V**
Adipocytes	1549.6	2661.5	837.0	2569.5	2061.7	2016.4	3365.9	2788.0	1705.2	1308.5	2501.0
Astrocytes	1693.9	1733.7	660.6	2178.4	1820.8	2108.6	2033.9	2315.7	1811.9	1189.0	2065.8
B-cells	1380.6	1173.9	1109.3	1949.5	1631.9	672.8	1050.4	1446.6	1000.0	1596.5	1003.4
Basophils	1150.0	1522.4	815.9	1141.9	1613.2	1588.1	2059.4	1555.1	1195.0	949.8	1537.1
CD4+ T-cells	1240.7	1150.9	1051.4	929.8	1592.9	1182.3	838.6	1712.1	1291.5	1123.5	1221.1
CD4+ Tcm	989.9	1290.0	825.5	839.0	966.7	1227.8	519.3	909.2	1062.7	876.5	1164.6
CD4+ Tem	706.1	1840.4	1468.6	778.4	1080.5	1447.2	1471.9	612.2	1742.5	998.4	1578.7
CD4+ memory T-cells	791.9	1005.5	1119.9	1160.3	1102.1	1100.1	1411.9	1331.9	1654.3	902.3	1387.5
CD4+ naive T-cells	1069.0	1222.5	1022.1	1451.3	1562.9	1442.0	1075.1	1758.9	1044.5	1017.6	1551.8
CD8+ T-cells	509.0	1396.2	876.8	1507.0	1541.3	1467.7	1411.3	1568.3	1155.6	651.9	1385.3
CD8+ Tcm	474.8	1354.5	259.5	1827.7	1125.3	1543.9	1744.9	1038.4	1437.4	774.0	1931.8
CD8+ Tem	1063.7	1414.9	655.8	1059.9	1340.0	1629.1	1546.3	1215.1	1178.1	1011.6	1607.7
CD8+ naive T-cells	662.4	879.7	980.4	1485.9	1074.2	503.4	1733.0	897.4	1876.1	1020.9	1135.7
CLP	417.0	748.0	1590.2	1263.5	766.3	1223.5	1412.9	995.7	1031.4	1203.5	611.9
CMP	1824.2	1850.0	1346.5	2061.6	2235.9	1930.4	2526.7	2172.7	2140.1	1663.2	1606.4
Chondrocytes	1220.8	971.2	533.8	1203.6	1160.8	1417.2	1334.9	860.0	1364.5	870.3	1096.5
Class-switched memory B-cells	1317.2	1697.8	910.4	2090.6	2047.5	2744.6	1597.7	1880.4	1986.8	2001.5	1372.5
DC	998.6	2934.8	984.8	2575.8	1114.0	1708.7	3733.4	1425.4	1681.9	1055.1	3881.8
Endothelial cells	1938.7	2058.3	1068.8	1937.1	1918.5	1932.6	1938.2	1769.4	1523.4	1245.0	2145.1
Eosinophils	987.1	1395.1	824.3	1505.1	1057.5	1719.3	2076.7	758.1	1434.3	739.4	1729.3
Epithelial cells	3172.1	1752.3	4120.1	1299.5	712.9	1290.2	1207.6	1027.7	2052.0	3176.4	1793.8
Erythrocytes	1243.0	2561.0	993.4	2005.7	1185.1	1732.0	3429.1	1548.5	1643.7	693.4	3424.9
Fibroblasts	1367.1	833.9	786.4	1231.4	1403.8	1549.1	548.5	1414.6	1250.2	1457.7	705.8
GMP	1264.0	1704.1	1323.7	1164.2	1260.2	1083.8	2105.2	1350.6	1714.9	1090.5	1884.0
HSC	1504.5	2804.2	852.9	1799.0	1503.7	2247.9	2692.3	2235.6	1382.1	1056.4	3358.2
Hepatocytes	1040.4	2155.2	534.8	1542.8	1341.1	1571.5	2355.3	1461.5	1306.3	1048.2	2577.8
Keratinocytes	1690.9	1726.9	2684.2	1473.6	688.5	1108.8	1524.6	600.4	1256.1	1791.8	1776.9
MEP	978.7	2003.6	1080.3	1446.4	1163.0	1497.2	1931.2	1196.2	858.6	753.3	1983.3
MPP	1141.5	2277.0	1295.9	2059.6	1946.7	1534.0	2154.9	1686.3	1687.7	1440.9	2132.6
MSC	913.5	1577.3	1618.1	1304.1	1154.5	2072.6	1306.7	804.2	1204.4	1338.2	1361.7
Macrophages	707.5	1306.8	1425.3	898.2	1131.9	1068.7	1342.4	706.7	1132.8	985.9	1430.8
Macrophages M1	620.2	1375.4	1234.8	687.2	952.1	1343.6	1625.9	386.7	945.1	574.6	1780.7
Macrophages M2	1287.2	1584.7	1465.2	1436.6	1755.5	1534.3	1369.2	1528.1	1399.8	1193.5	1333.8
Mast cells	787.7	173.6	950.4	822.5	1323.2	338.5	621.6	929.7	373.2	958.1	257.4
Megakaryocytes	1928.5	1621.8	1574.6	2795.6	1601.8	2310.0	1360.3	1883.4	700.3	1549.0	1357.6
Melanocytes	1569.1	1194.1	1064.2	2003.0	1763.8	1163.8	938.6	1658.0	1350.8	1448.7	1374.0
Memory B-cells	1179.0	1237.9	576.5	1771.9	1542.2	1093.1	1249.1	1215.9	1565.5	1120.1	1195.4
Mesangial cells	1920.2	1997.6	1393.1	1609.0	2003.0	1959.4	2202.0	1667.6	1633.1	1212.3	2387.2
Monocytes	1033.4	1871.9	2170.4	1365.9	1970.7	1816.2	1129.6	1792.1	1866.2	1802.8	1689.8
Myocytes	1272.4	1774.0	448.6	1924.0	1385.9	1956.2	1712.8	1336.8	998.2	1075.6	1788.7

NK cells	1257.1	1589.0	573.1	1305.9	1889.7	1878.2	1725.0	1619.4	1482.5	1403.8	1424.0
NKT	1935.7	2079.1	989.5	1144.3	1855.3	1511.8	1910.1	1212.1	1803.4	1260.5	2000.3
Neurons	1816.7	1917.9	673.5	1757.2	1873.9	1600.8	1811.0	1942.5	1815.2	1330.9	1996.3
Neutrophils	854.1	2118.2	2245.5	2005.3	2129.3	2904.9	2228.7	2532.3	2244.6	1478.1	2601.8
Osteoblast	758.8	759.9	1000.0	662.1	1079.5	924.1	432.5	835.2	1029.2	861.4	441.5
Pericytes	1879.7	1846.0	1637.5	1890.3	1675.8	1938.7	2263.2	1880.0	1840.6	2005.0	2026.5
Plasma cells	1399.8	947.9	957.4	1449.5	1300.0	1518.8	748.9	1456.6	776.0	1095.1	624.9
Platelets	655.1	1006.3	553.4	1028.7	466.0	1398.4	969.6	898.2	938.0	813.3	1043.0
Preadipocytes	1266.6	1155.6	1257.5	1527.3	1576.8	1464.9	1283.0	1661.6	1633.0	1624.4	1718.0
Sebocytes	1566.6	1561.3	2450.7	1469.7	766.1	1138.9	1248.6	716.4	1478.2	1590.8	1624.5
Skeletal muscle	1449.9	2769.9	792.2	1915.0	1311.4	2250.7	3125.1	1622.0	1812.6	923.3	3609.2
Smooth muscle	1129.0	1112.1	1625.9	1144.6	986.5	631.5	1056.3	1122.1	1252.7	1104.0	990.7
Tgd cells	139.8	643.7	174.4	552.8	495.2	253.3	600.7	453.2	423.4	166.2	471.5
Th1 cells	578.6	815.1	1567.8	647.2	456.4	633.0	1420.4	890.4	1341.8	712.0	1615.8
Th2 cells	1525.7	1866.0	240.5	2870.9	1626.9	1083.4	2416.2	2057.6	810.2	1028.9	1855.2
Tregs	1856.7	1952.9	1944.9	2377.9	2922.1	2080.1	1877.7	2761.4	2845.9	2010.9	2302.4
aDC	1090.4	2440.1	1402.2	1804.5	1523.2	1942.5	3172.5	1897.8	1499.4	858.4	3234.0
cDC	1113.7	2987.5	1138.7	2329.9	1747.8	1624.1	2765.6	2109.7	2357.4	1241.3	3462.4
iDC	1160.7	3137.4	1741.8	2464.6	1203.5	1454.9	4210.0	564.1	2122.3	1678.9	4168.0
ly Endothelial cells	1547.1	1819.7	1339.4	2721.6	2222.7	2859.3	2028.8	2137.8	1462.4	1590.4	2169.8
mv Endothelial cells	990.5	1023.5	806.2	1457.5	1055.6	1395.0	928.6	852.1	981.0	949.6	951.0
naive B-cells	1138.5	1512.4	713.4	1423.6	1378.9	933.9	1164.7	1501.5	1085.6	1391.9	1024.2
pDC	1162.6	2231.8	431.7	1948.6	2110.3	2260.4	2539.2	1826.4	3317.0	1749.8	2436.6
pro B-cells	1091.2	1790.8	760.0	1571.7	906.6	1633.4	2453.3	1192.7	1842.7	993.9	2215.6

**Cell-type distribution**	**IGIB1130515988V**	**IGIB1130508933V**	**IGIB1130493051V**	**IGIB1130482058V**	**IGIB1130469734V**	**IGIB1130429966V**	**IGIB1130412732V**	**IGIB1130371022V**	**IGIB1130359925V**	**IGIB1130341678V**	**IGIB1130228653V**
Adipocytes	2700.6	2718.2	2004.3	1555.3	2659.1	2019.8	3029.4	1736.3	2022.2	1261.1	1957.9
Astrocytes	2083.4	2218.1	1601.6	1468.1	2108.3	2059.2	2288.3	1969.3	1766.4	1825.2	1875.4
B-cells	1203.3	2167.5	1611.5	1473.1	1189.2	1592.3	781.7	1605.8	1570.0	1275.4	1482.1
Basophils	1690.8	1897.3	1545.4	974.9	1663.0	1452.0	1684.1	1728.7	1553.5	1241.9	1173.9
CD4+ T-cells	1123.7	918.8	1495.8	1234.5	1090.0	896.5	1241.9	1523.9	1547.1	882.5	1482.6
CD4+ Tcm	759.8	1056.9	844.8	710.4	757.9	948.3	708.9	1037.4	866.2	886.5	955.5
CD4+ Tem	1196.4	1047.7	915.1	840.9	1164.6	1329.5	770.6	1124.7	1163.2	837.7	1148.9
CD4+ memory T-cells	1529.8	1252.1	1252.9	1000.1	1492.3	712.4	1162.3	1002.3	1311.8	836.9	1143.3
CD4+ naive T-cells	1231.5	1007.2	1162.5	1095.4	1197.2	1286.8	1160.4	1551.9	1300.4	1186.1	1514.0
CD8+ T-cells	1586.4	1225.1	1188.0	922.4	1604.1	1260.9	1184.3	1620.1	1011.8	1287.6	1450.8
CD8+ Tcm	2129.0	1447.0	1221.8	531.9	2093.1	1184.4	1783.4	1177.0	664.0	755.2	1180.6
CD8+ Tem	1677.4	1628.0	1475.0	782.8	1653.7	1776.3	1541.1	1295.3	840.2	1375.8	1067.9
CD8+ naive T-cells	1422.0	1681.0	1057.2	560.0	1388.7	351.9	1052.7	1185.5	692.1	709.0	1131.0
CLP	1053.0	1504.2	1652.3	1401.3	1304.9	201.7	1285.8	1166.8	1193.6	1405.1	1174.7
CMP	1878.3	1887.9	2447.7	1483.2	1856.2	1689.9	1851.1	1942.7	2124.0	2052.5	1877.9
Chondrocytes	804.1	1721.8	1305.2	1010.7	780.5	1158.6	717.0	791.8	963.4	1632.4	978.9
Class-switched	1463.4	2917.8	2247.5	2190.6	1433.3	2544.9	1993.7	2171.1	2210.7	2377.3	2328.8

memory B-cells											
DC	4413.4	3270.4	1828.2	1108.2	4364.6	1084.9	3791.7	767.7	1291.9	1257.7	1518.2
Endothelial cells	1714.2	2555.8	1640.0	1544.9	1698.1	2023.7	1755.6	1635.9	1675.0	2072.0	1770.0
Eosinophils	2080.2	1660.2	1079.0	747.9	2053.6	1470.6	1722.6	1184.1	886.1	1016.7	1395.1
Epithelial cells	1403.5	856.1	2524.7	3097.6	1394.7	995.6	1051.9	479.3	928.8	2652.3	2605.8
Erythrocytes	3917.8	2602.5	2050.8	531.3	3877.8	1234.2	3435.0	1253.2	1411.1	1564.6	2045.9
Fibroblasts	621.6	896.2	1655.6	1283.3	646.0	1429.6	874.2	1481.5	1595.9	1319.0	1533.5
GMP	2045.7	1741.1	2194.1	922.1	2012.9	763.6	1598.0	1037.4	1056.9	682.6	938.7
HSC	3207.8	2505.1	3290.2	1001.1	3168.7	1379.1	3197.9	1929.0	1985.3	1913.8	1461.9
Hepatocytes	2725.6	2222.0	1426.0	925.6	2709.8	1457.3	2337.0	1388.9	1323.4	1274.7	1505.2
Keratinocytes	1628.5	1193.3	1471.5	1663.2	1602.1	1223.3	1485.4	416.5	616.7	1832.5	1916.4
MEP	2189.5	1867.0	1342.0	752.7	2162.1	916.5	1982.2	843.9	912.2	966.4	887.7
MPP	2236.1	1571.6	2286.1	954.4	2203.0	1088.0	1828.5	1302.3	1318.3	1558.6	1434.6
MSC	1457.7	2151.4	1672.0	1278.9	1439.4	1629.9	2173.5	1466.2	1267.1	1587.8	1869.4
Macrophages	1722.4	981.1	1397.4	704.6	1689.5	778.6	1319.9	823.3	1076.6	751.7	506.6
Macrophages M1	2118.5	1105.9	1266.0	437.7	2083.7	533.0	1568.6	487.7	723.8	746.2	560.9
Macrophages M2	1563.5	1547.0	1637.3	1277.1	1544.2	1225.2	1386.1	1279.2	1645.0	1520.6	1129.3
Mast cells	371.1	592.2	862.2	953.3	406.7	1132.1	36.7	992.8	1107.7	850.0	845.6
Megakaryocytes	1805.7	1823.7	1901.2	1494.4	1779.2	1734.3	1519.2	1981.8	1620.3	1897.0	1705.7
Melanocytes	1064.5	1757.3	1595.2	1151.2	1048.9	1245.4	1562.2	1687.8	1960.2	1155.7	1094.5
Memory B-cells	1590.7	2200.2	1489.2	1001.7	1560.8	1848.3	1531.4	1384.9	1151.3	1027.8	1697.0
Mesangial cells	2417.8	2297.9	1486.8	1307.2	2395.5	2378.3	2703.5	1762.6	1516.9	2001.4	1948.1
Monocytes	1317.7	1173.6	1583.9	1500.6	1289.0	1871.8	1447.0	2172.5	1933.1	1766.0	1406.5
Myocytes	1858.5	2613.5	1408.5	1220.4	2087.5	1840.6	1850.8	1298.5	1374.0	1129.2	1140.9
NK cells	1693.1	1888.9	2172.6	1033.4	1661.6	2110.2	1216.0	1951.3	1526.1	1415.6	1505.2
NKT	1859.8	1657.9	1524.6	1167.0	1826.3	2408.1	1742.3	1853.5	1719.6	1100.0	1237.1
Neurons	1978.1	2000.2	1672.5	1288.3	2035.8	1696.8	1885.4	1889.9	1817.5	1746.2	1973.2
Neutrophils	2427.0	2512.2	2247.3	1395.0	2389.5	1526.8	2506.8	1919.6	2277.2	1842.1	1697.0
Osteoblast	467.7	578.9	1284.3	948.7	468.8	1000.4	703.2	799.9	794.1	927.7	1198.1
Pericytes	2114.8	1980.9	1662.9	1831.2	2243.2	2061.1	1793.8	1974.2	1758.2	1761.5	1957.7
Plasma cells	637.6	936.8	1373.4	1208.9	612.4	1332.1	1272.3	2079.7	1200.0	1365.3	1107.1
Platelets	1241.3	1301.9	943.2	628.5	1250.0	1131.4	1467.4	973.1	813.5	910.3	970.0
Preadipocytes	1176.0	1089.4	1073.8	1804.6	1140.6	2156.5	1554.7	1642.9	1680.7	1870.5	1573.1
Sebocytes	1742.2	1719.1	1309.7	1487.8	1708.9	1148.1	1432.9	467.1	434.2	1919.5	1989.3
Skeletal muscle	3402.7	3118.4	2618.0	1031.4	3366.1	1453.3	2933.0	1248.6	1306.6	2488.2	1898.2
Smooth muscle	1084.7	1250.6	1634.4	1436.5	1062.4	840.0	970.3	1036.2	1167.8	1173.2	1247.9
Tgd cells	701.8	339.5	487.8	20.6	677.3	546.6	264.1	495.7	474.9	389.7	212.7
Th1 cells	1423.6	1388.0	2055.6	605.3	1395.2	1852.7	1446.1	1310.4	364.5	1237.0	847.7
Th2 cells	2242.6	2062.9	1220.1	852.0	2205.8	1239.5	1735.8	2184.3	1466.2	1364.3	768.8
Tregs	1895.4	1787.2	2363.5	2480.0	1866.1	2480.5	1584.8	2624.2	2953.0	2022.8	2448.2
aDC	3614.1	2673.7	2523.6	902.0	3597.2	1307.4	3276.7	1274.0	1670.1	1578.4	1723.6
cDC	3510.4	2639.7	1639.4	1286.8	3464.6	1413.2	3049.1	1085.4	1610.5	1548.6	1652.4
iDC	4829.8	3484.7	1791.2	990.7	4777.4	864.8	3962.3	1183.6	1092.1	1430.0	961.7
ly Endothelial cells	1739.1	3030.3	1794.2	1859.9	1727.9	2605.3	1949.2	2333.6	2321.5	2678.1	2704.6
mv Endothelial cells	748.1	1467.6	816.8	1047.9	753.2	1367.3	1055.2	1013.4	990.4	1264.7	1120.7
naive B-cells	1152.0	2171.8	1517.8	1218.5	1186.8	1295.3	900.0	1499.1	1439.0	1183.3	1606.7
pDC	2197.2	1875.9	2140.1	1525.5	2163.8	1962.3	1522.6	1852.7	1814.0	1871.7	2366.5

pro B-cells	2163.2	2036.1	1276.3	882.5	2130.9	1257.3	1814.9	1276.4	1392.2	939.3	1080.1

**Cell-type distribution**	**IGIB1130222666V**	**IGIB113094002V**	**IGIB113059193V**	**IGIB113050041V**	**IGIB113023885V**	**IGIB1130111V**	**IGIB113081916**	**IGIB113071549**	**IGIB113071357**	**IGIB113052554**	**IGIB113042520**
Adipocytes	1889.7	264.6	1807.6	914.6	1645.6	2000.5	1365.1	1146.5	1793.1	2061.5	716.5
Astrocytes	2167.0	1305.1	2220.9	851.5	2154.1	1576.7	1202.1	1889.3	1852.1	1822.5	2024.1
B-cells	1813.6	1603.3	1307.8	934.6	1928.6	1640.5	544.2	1731.2	888.4	339.4	1327.1
Basophils	1305.9	988.8	774.0	705.1	1517.0	1039.0	835.1	1447.0	1119.8	1304.0	1607.8
CD4+ T-cells	1432.8	987.0	858.3	1227.5	1543.2	1606.5	1195.2	876.4	1444.4	1305.5	910.8
CD4+ Tcm	917.4	753.7	1472.4	737.6	889.5	1012.6	443.4	984.5	668.7	1203.3	1075.1
CD4+ Tem	960.3	1218.4	1493.6	1002.6	816.4	1162.4	971.2	1558.2	1002.8	1455.8	1687.6
CD4+ memory T-cells	982.1	979.7	405.7	1079.5	1326.7	1239.8	1472.3	604.8	1399.0	995.3	354.6
CD4+ naive T-cells	1404.4	746.2	1240.1	1288.0	1441.7	1447.7	1260.1	719.3	1486.7	1235.7	1294.5
CD8+ T-cells	1245.6	839.6	981.7	758.4	1259.4	1346.6	1123.5	1078.9	1650.7	1721.8	924.1
CD8+ Tcm	843.8	809.8	948.6	304.5	1307.2	1018.9	1563.5	1021.0	1802.9	1749.2	432.7
CD8+ Tem	1264.2	776.7	1527.0	534.4	1040.4	1212.6	1191.1	1488.7	1203.0	1862.2	1672.8
CD8+ naive T-cells	974.6	1152.6	443.0	317.4	1242.2	905.5	1248.2	641.0	1234.0	671.5	34.2
CLP	679.5	1258.6	626.8	1889.6	741.2	1271.9	2110.2	1249.4	1500.4	442.1	168.0
CMP	1963.0	1188.2	2155.5	1097.9	1899.9	1802.4	1530.2	1803.6	2123.1	1621.3	1609.0
Chondrocytes	1052.1	900.2	1412.7	1173.1	1101.2	792.1	565.5	1621.7	1008.3	703.0	1849.4
Class-switched memory B-cells	2392.9	1999.2	2368.6	1937.8	2116.8	2330.1	707.2	2068.0	988.1	1493.8	2648.8
DC	1133.8	841.2	1694.9	760.9	998.4	1356.5	2767.0	1505.1	2367.5	2888.8	586.8
Endothelial cells	1566.4	1436.3	1723.5	1299.8	1627.0	1101.1	943.7	2049.8	2216.1	1637.6	1906.5
Eosinophils	801.7	1209.3	2153.2	1133.8	538.2	755.3	800.5	1403.6	1010.9	1512.8	1288.8
Epithelial cells	655.5	3185.8	1552.8	3887.4	31.3	1651.3	2716.6	1463.3	2579.4	2835.3	2078.3
Erythrocytes	1147.2	804.2	1665.9	420.8	1207.4	458.8	2615.3	1174.8	2357.2	2768.1	424.1
Fibroblasts	1385.7	1228.0	1555.4	1024.0	1459.9	1306.3	718.9	1348.1	1102.2	874.9	1439.0
GMP	933.1	718.9	1155.8	1148.2	1049.2	987.0	1695.4	1138.5	1708.6	1616.5	532.2
HSC	1663.9	1921.1	583.9	1532.4	2126.1	1409.3	2011.3	1758.8	1612.8	2122.6	527.9
Hepatocytes	1190.1	1012.0	1225.8	695.9	1548.4	1028.6	1490.9	1600.9	1777.3	1970.0	1021.5
Keratinocytes	561.3	1822.9	1497.9	2430.2	40.7	481.2	1839.9	1155.0	1344.3	2204.7	1613.1
MEP	992.5	1122.2	1110.8	717.2	971.4	714.5	1171.6	1351.4	1384.0	1916.5	665.4
MPP	1328.3	1197.7	1375.9	730.4	1024.5	1520.3	1553.9	1795.2	1422.7	1507.6	1312.0
MSC	1529.9	1419.5	1997.5	1007.2	1098.5	978.4	1025.6	1985.1	1471.0	1966.3	2326.0
Macrophages	1160.8	1053.4	733.5	1067.7	838.7	900.1	1241.4	715.4	1190.6	1151.7	881.8
Macrophages M1	820.9	577.7	837.5	937.3	329.8	931.6	1381.1	817.6	932.0	1132.4	737.9
Macrophages M2	1729.4	1527.2	1640.6	1598.6	1068.0	1213.9	1172.0	988.0	1216.4	1306.0	1502.3
Mast cells	1067.6	1282.7	822.4	1142.6	845.8	1023.0	338.9	653.9	783.5	33.7	827.0
Megakaryocytes	1282.4	1658.8	1329.1	1193.3	2114.6	1397.6	931.8	1638.4	1634.1	1473.0	2093.4
Melanocytes	1824.9	1313.2	1850.4	1575.9	1866.9	1707.4	1022.3	1947.4	1079.8	1024.1	2165.8
Memory B-cells	1398.2	1299.8	1828.0	1004.9	1581.1	1420.9	535.9	1569.7	1243.1	728.7	982.1
Mesangial cells	1806.2	1174.5	2781.0	1033.2	1899.7	1551.5	1474.8	2177.3	1628.1	2059.5	2036.9
Monocytes	1923.9	1873.0	1241.9	2087.6	1548.6	1609.6	1158.9	2626.4	889.2	1450.4	2505.4

Myocytes	1052.8	1270.3	1729.8	1154.2	1325.3	852.7	1758.1	1607.9	1714.4	1633.6	1528.4
NK cells	1506.5	1250.7	1309.4	863.1	1651.3	1705.8	1034.7	1607.4	1615.2	1624.6	1723.6
NKT	2184.4	1219.3	2439.6	1382.9	1294.9	1591.8	1077.1	2120.1	1128.1	1682.6	2652.0
Neurons	1627.2	890.7	1883.7	666.1	1976.3	1426.9	1044.1	1780.4	1377.9	1615.8	1545.1
Neutrophils	1930.4	1948.6	1852.1	2230.3	1494.6	1946.0	2100.8	3168.7	1231.8	1871.1	4270.1
Osteoblast	839.8	916.6	872.9	1087.3	867.3	736.3	975.9	924.0	1045.8	657.0	662.8
Pericytes	2075.3	1463.3	2145.1	1156.1	1726.1	2007.3	1507.8	1894.8	1351.3	1788.2	2326.8
Plasma cells	1371.9	1264.6	1681.1	1723.8	1177.7	1358.3	287.8	1196.9	658.9	1226.8	1570.7
Platelets	898.6	339.1	1183.3	142.3	1101.7	791.3	1178.0	407.5	655.4	713.5	699.1
Preadipocytes	1841.0	1498.8	2043.9	1394.0	1768.2	1533.3	1388.2	1632.9	1238.3	1150.2	1856.3
Sebocytes	645.5	1779.0	759.7	2194.1	0.0	162.3	1899.4	1085.9	863.5	2003.3	1427.0
Skeletal muscle	1293.1	1044.1	2057.9	1090.8	1407.6	781.5	1851.5	2055.1	2098.6	2581.5	1716.0
Smooth muscle	882.0	1793.0	872.2	1813.8	1118.9	1060.6	2060.0	666.7	2003.4	606.4	254.8
Tgd cells	530.9	143.3	366.3	196.4	471.5	571.5	813.6	519.3	437.1	443.5	200.0
Th1 cells	1054.4	1008.9	1648.7	1121.2	900.9	982.9	1242.2	1077.8	1975.4	779.0	1034.2
Th2 cells	1229.4	692.1	2077.8	798.8	2441.9	1262.9	1369.4	2137.2	1363.3	1610.3	989.6
Tregs	2720.0	1502.7	2479.2	1539.0	2853.6	2459.5	1864.2	1834.6	1917.1	2371.6	1651.8
aDC	1323.3	710.8	1565.8	1106.4	1298.6	1936.8	2543.5	1706.1	1950.0	2520.8	769.8
cDC	1380.4	1712.5	1618.9	1496.7	1439.0	1181.7	2378.7	1856.9	1869.2	2713.4	918.0
iDC	637.2	1628.8	1650.3	1136.1	1018.2	1426.2	3223.7	1549.5	2662.0	2905.3	511.8
ly Endothelial cells	2378.6	2132.8	3028.6	1668.5	1983.8	1590.9	755.2	2523.7	2518.1	2525.7	2991.5
mv Endothelial cells	1072.0	1025.0	1254.4	856.6	772.2	777.3	641.2	1343.6	1298.9	783.0	1238.7
naive B-cells	1561.5	1309.9	1374.1	566.7	1554.2	1136.3	717.4	1406.2	762.0	465.4	1348.4
pDC	1856.7	1776.3	2462.2	1352.1	1895.2	1377.5	1126.9	2751.4	1732.1	1896.8	2154.2
pro B-cells	1082.8	874.7	1518.0	718.2	1111.1	793.0	1246.8	1582.3	1296.5	1503.9	1042.4

**Cell-type distribution**	**IGIB113041961**	**IGIB113011563**	**IGIB11303953**	**IGIB11303770**	**IGIB11303722**	**IGIB11303711**	**IGIB11303605**	**IGIB11303602**	**IGIB11303580**	**IGIB11303563**	**IGIB11303523**
Adipocytes	2359.8	1308.4	491.4	1824.0	604.1	1675.1	1025.8	2116.8	220.2	2794.6	2433.0
Astrocytes	1240.2	1894.7	248.4	1707.6	0.0	2233.1	1691.5	1462.2	786.3	2179.9	1979.0
B-cells	183.3	1623.0	732.2	1880.9	3741.4	1620.9	1648.1	1488.3	656.1	668.8	1062.5
Basophils	1231.8	1327.0	774.7	1266.6	670.2	1375.9	1355.0	1297.7	731.4	1746.4	1550.6
CD4+ T-cells	1054.5	1595.5	1040.6	928.2	2823.7	910.5	737.3	807.4	798.6	767.8	1090.0
CD4+ Tcm	1308.6	968.4	510.5	997.0	1329.3	1422.8	1556.0	540.5	567.3	812.8	1064.9
CD4+ Tem	2136.3	865.8	1245.4	1436.3	2464.5	1228.8	1756.9	1209.5	1320.5	948.9	1197.2
CD4+ memory T-cells	1157.0	1093.0	1120.0	760.5	3087.5	832.3	830.5	1410.6	1227.2	944.0	1578.3
CD4+ naive T-cells	1112.3	1454.0	943.8	1080.3	2755.9	1501.0	1346.9	1009.6	900.6	874.2	1207.9
CD8+ T-cells	1567.8	624.9	528.1	1333.0	1995.4	975.6	1003.1	1311.7	579.9	1285.5	1437.2
CD8+ Tcm	991.0	1084.7	529.4	1067.4	2166.7	1316.8	894.1	1486.4	298.3	1496.4	1895.5
CD8+ Tem	1161.4	1114.4	475.4	1301.6	1491.0	1258.0	1435.9	1126.0	317.7	1948.1	1586.5
CD8+ naive T-cells	684.1	851.5	847.0	766.6	1313.5	1394.9	842.1	802.5	730.2	1135.1	1549.6
CLP	1306.9	1050.5	1791.1	590.9	2507.0	812.8	1014.7	1129.8	1997.3	511.2	833.3
CMP	1478.4	2059.8	824.4	1956.8	959.3	1948.8	1727.3	1419.0	1030.7	1796.5	2201.1
Chondrocytes	1227.2	1717.3	410.1	1211.0	694.3	1285.4	1554.5	917.4	699.0	1411.8	677.7
Class-switched memory B-cells	1303.5	2528.3	1421.2	2278.9	3164.4	2138.6	2283.8	1903.1	1586.0	972.2	1265.0
DC	2041.1	1360.1	218.6	1357.1	1378.7	1755.1	957.9	3394.7	400.7	2352.7	4225.3

Endothelial cells	947.7	1774.3	320.8	1860.3	257.0	2175.4	1437.2	1836.6	703.9	1993.9	2251.6
Eosinophils	1512.6	1046.0	418.0	1077.5	240.1	1419.0	869.4	1584.4	591.5	1205.3	2087.2
Epithelial cells	3082.1	2284.1	4065.7	2576.5	995.8	1535.9	1835.5	2615.0	3426.9	1937.5	1500.9
Erythrocytes	1766.8	524.3	131.5	1301.9	909.1	1814.7	686.5	2773.6	272.1	2749.5	3664.4
Fibroblasts	1004.8	1247.2	742.2	1534.9	700.0	1386.9	1130.3	879.8	909.9	1294.0	577.7
GMP	938.5	1076.0	810.3	1468.8	1301.1	1202.4	705.1	1593.4	928.1	1396.2	1928.6
HSC	1372.0	1295.1	606.0	1756.7	1319.9	2565.7	389.8	2205.8	1102.7	2664.3	3144.5
Hepatocytes	1175.0	1196.5	221.9	1268.9	172.8	1688.4	1227.3	2163.0	475.6	1767.8	2556.3
Keratinocytes	2300.4	1172.5	2384.4	1488.0	355.3	1586.4	1203.4	2045.2	1829.2	1902.9	1765.1
MEP	1329.3	1075.7	652.4	1043.8	887.2	1766.6	607.3	1214.5	826.6	1877.1	2046.9
MPP	1268.9	1741.4	522.8	2114.9	1634.7	1335.5	1404.1	1733.4	924.9	1732.3	2212.5
MSC	1652.6	1793.5	1214.9	1428.8	975.2	1557.9	1663.3	1385.9	1249.7	2202.1	1278.4
Macrophages	1069.4	998.1	895.5	936.8	1386.3	885.9	807.4	1356.1	1131.9	886.0	1629.2
Macrophages M1	891.3	914.6	424.2	776.8	707.4	1009.2	716.7	1379.9	618.3	686.2	2031.0
Macrophages M2	1018.7	1940.7	934.6	1336.6	1392.5	1013.1	1292.2	1442.3	1291.5	1145.4	1431.4
Mast cells	867.5	998.3	964.4	1059.4	1622.6	994.3	913.3	460.1	1143.4	726.6	260.1
Megakaryocytes	2067.6	2397.6	869.8	1093.6	249.3	709.8	1200.8	2488.6	612.2	1540.9	2277.8
Melanocytes	825.9	2283.0	1019.2	1822.2	1174.9	1201.5	1839.2	1791.2	1230.0	1636.7	973.6
Memory B-cells	704.9	1743.0	317.7	1618.0	2672.0	1996.9	1266.3	1190.0	291.4	1445.7	1423.6
Mesangial cells	1212.3	1832.6	628.9	1993.9	197.2	2043.9	2102.4	1780.5	487.5	2697.0	2244.6
Monocytes	1958.0	1944.3	1219.4	2866.2	2401.5	1944.0	3209.2	2511.5	2347.8	1625.3	1747.8
Myocytes	1440.5	1316.8	588.6	1504.4	744.0	1446.3	1589.1	1653.1	924.3	1395.1	1998.5
NK cells	895.9	1338.2	823.6	1409.5	1869.4	1795.8	906.9	1198.6	819.5	1305.5	1603.5
NKT	2289.7	1757.8	387.7	1959.5	767.0	1416.9	2340.6	1741.9	1120.7	1875.3	1636.6
Neurons	964.6	1530.0	36.1	1625.4	176.7	1884.0	1257.3	1485.5	430.2	2084.9	1867.9
Neutrophils	3680.6	2816.3	2315.9	3597.3	3513.5	3647.7	4052.4	3352.1	3244.9	2450.8	2702.2
Osteoblast	1040.6	1130.1	1162.0	756.3	916.4	1087.5	933.0	634.4	1272.3	1236.6	700.0
Pericytes	2058.7	1969.8	921.9	2104.6	1073.7	1759.3	1774.5	2203.2	907.2	2068.7	2383.4
Plasma cells	754.4	1538.7	1009.0	990.6	1826.2	890.0	1068.8	412.1	1035.3	804.2	485.2
Platelets	796.6	689.7	328.5	581.5	366.5	896.1	624.4	887.1	556.5	1179.3	1588.9
Preadipocytes	1075.5	1786.0	1207.7	1820.7	1121.2	1317.5	1362.7	2077.2	1507.6	1653.4	1031.1
Sebocytes	1964.1	925.9	2213.9	1393.3	302.9	1263.0	822.5	1995.2	1426.1	1845.9	1825.7
Skeletal muscle	1416.8	1761.0	87.3	1311.0	327.1	1845.0	1349.5	2224.3	127.4	2291.1	3243.7
Smooth muscle	1562.9	1246.4	1995.8	1063.4	2097.7	721.3	734.6	1131.1	2219.4	1167.2	956.2
Tgd cells	231.3	254.2	280.8	516.5	1275.9	690.5	270.5	402.3	503.1	77.8	686.1
Th1 cells	1092.2	1285.1	1716.1	694.0	1747.9	1012.8	737.1	1183.8	1903.0	1775.9	1235.8
Th2 cells	1002.7	1321.7	333.9	894.5	1451.1	860.2	1094.3	1497.9	593.0	1849.4	2001.1
Tregs	2115.7	2353.3	972.0	2782.0	3094.8	2469.0	2077.9	1107.5	1077.0	1908.2	1697.1
aDC	1766.9	1989.4	605.8	1947.0	2174.3	1681.2	1717.0	2745.8	526.4	2423.7	3470.4
cDC	1866.9	1106.5	571.6	1791.5	2084.7	1893.9	1697.8	3409.3	1070.9	2128.2	3311.4
iDC	2419.4	1695.4	368.7	1843.9	1523.8	1284.1	1420.2	3878.0	667.0	2430.4	4637.3
ly Endothelial cells	1001.4	2658.1	291.0	2788.6	453.7	2855.8	1842.3	1840.3	788.9	2551.5	2148.3
mv Endothelial cells	506.6	1053.1	580.1	1128.6	479.4	1276.1	1031.3	749.6	679.1	1015.1	1044.6
naive B-cells	621.6	1639.7	358.8	1737.1	3078.1	1723.5	1241.8	1276.7	352.2	823.9	1021.2
pDC	1719.7	1776.0	631.3	748.0	1310.4	2296.3	1671.0	1088.6	850.2	1761.4	2035.8
pro B-cells	1169.0	1217.3	145.1	1014.3	989.0	1300.3	943.1	1461.1	375.9	1695.8	2027.1

Cell-type distribution	IGIB11302559	IGIB11302272	IGIB11302264	IGIB11302237	IGIB11301955	IGIB11301910	IGIB11301829	IGIB11301798	IGIB11301795	IGIB11301775	IGIB11301748	IGIB11301567
Adipocytes	1881.6	732.5	2676.2	864.3	2612.0	2222.6	601.1	2701.0	2247.3	2506.6	2054.2	1367.1
Astrocytes	2184.9	821.1	2119.4	709.3	1973.2	1785.0	581.5	2083.7	1593.5	1917.5	1583.2	1395.8
B-cells	1289.8	1089.6	1168.8	2007.3	764.5	1549.5	1242.6	1203.7	1351.4	1117.7	1531.3	1754.9
Basophils	1192.5	442.4	1630.1	1459.6	2148.6	1613.2	885.6	1691.1	1609.0	1800.9	1391.6	1269.2
CD4+ T-cells	545.0	913.4	1197.0	1317.2	998.5	1346.8	1089.6	1124.0	835.1	1182.5	1067.3	1071.0
CD4+ Tcm	1394.5	735.6	810.4	713.7	677.6	589.3	705.0	760.2	948.9	788.6	421.0	513.9
CD4+ Tem	2209.3	1104.4	1163.5	1709.1	1215.7	1147.4	1277.3	1196.8	1564.3	954.1	1217.7	1057.7
CD4+ memory T-cells	597.2	904.4	1370.6	1657.8	1277.2	1483.0	1147.4	1530.2	1221.1	1392.3	1467.0	796.3
CD4+ naive T-cells	910.5	930.9	1115.8	1648.6	903.6	1021.7	1211.4	1231.9	1083.9	1304.9	970.1	983.6
CD8+ T-cells	1139.1	724.4	1748.2	1268.9	1364.4	1347.4	908.4	1586.8	1436.0	1425.6	993.9	928.0
CD8+ Tcm	1068.0	428.9	1915.9	1206.6	1624.5	1740.2	575.5	2129.4	1935.7	1891.6	1044.3	1178.9
CD8+ Tem	1599.8	350.9	1814.3	683.9	1554.3	1497.7	931.7	1677.8	1440.2	1760.1	868.6	859.9
CD8+ naive T-cells	1019.0	627.5	1224.8	868.1	728.1	1509.8	489.7	1422.3	1080.2	1352.9	1683.4	1453.2
CLP	1256.6	1845.3	1182.1	1478.3	1366.5	1828.9	1479.7	1053.3	1413.7	1059.2	1684.9	1851.0
CMP	1658.4	1153.6	2097.1	1315.7	2072.0	1911.2	823.0	1878.6	1728.3	2079.5	1536.3	1999.8
Chondrocytes	1385.1	612.3	792.0	420.8	1204.0	682.0	773.7	804.4	819.4	761.8	659.7	878.6
Class-switched memory B-cells	2105.5	991.7	2026.7	2256.5	1407.5	1285.5	1904.0	1463.8	1743.1	1901.3	1934.0	1681.9
DC	2014.5	417.7	4104.2	1724.1	3528.5	4021.7	387.2	4414.2	3834.5	3962.9	2945.5	2454.2
Endothelial cells	1991.4	481.8	2296.1	680.3	1883.7	1475.6	1143.7	1714.6	2394.7	1602.8	850.1	872.7
Eosinophils	1666.6	287.7	2063.0	1004.1	1533.1	1944.0	705.5	2080.6	1902.5	2035.3	968.9	1052.1
Epithelial cells	3294.0	3220.7	1335.8	1892.7	1733.9	1980.2	4097.1	1403.7	2423.0	1443.7	2591.5	2456.6
Erythrocytes	1607.4	558.5	3633.8	929.7	3155.8	3575.1	268.0	3918.2	3108.1	3562.1	2419.0	1869.8
Fibroblasts	853.1	804.6	565.8	1055.7	926.2	834.0	897.3	621.8	438.6	482.0	700.7	992.7
GMP	1168.8	877.2	1972.4	1477.9	1888.1	1952.7	818.4	2046.1	1809.1	1875.1	1744.0	1629.3
HSC	1662.6	1252.7	2912.5	1351.4	2401.5	2793.3	796.3	3208.3	2494.0	2802.8	1588.9	1508.1
Hepatocytes	1567.9	161.5	2513.8	721.3	2495.7	2410.9	455.0	2726.1	2195.1	2613.1	1544.6	1466.8
Keratinocytes	1875.8	1746.8	1596.2	939.3	1449.4	1886.1	2483.3	1628.8	1936.5	1761.2	1451.8	1609.4
MEP	1254.6	846.7	1971.9	391.2	2044.4	1863.6	329.4	2189.9	2081.2	1972.7	1546.9	1341.8
MPP	2167.6	413.2	2210.6	1266.2	1791.0	2616.3	1154.7	2236.5	1915.7	2048.9	1404.2	1827.4
MSC	1896.8	943.3	1362.2	589.7	2040.2	1211.9	1226.8	1458.0	1189.3	1394.1	970.5	1144.8
Macrophages	885.9	623.8	1497.5	2402.4	1486.5	1660.7	1586.5	1722.9	1525.8	1499.9	1562.0	1083.0
Macrophages M1	903.7	239.1	1819.4	2062.0	1692.3	1914.5	1274.6	2119.1	1678.4	1832.3	1221.8	896.3
Macrophages M2	1380.0	587.4	1815.8	2496.3	1655.2	1528.9	1691.1	1563.8	2051.3	1758.4	1657.0	1031.7
Mast cells	758.8	1124.5	548.0	1330.9	289.7	443.3	890.2	371.4	157.7	554.4	654.0	832.0
Megakaryocytes	1809.8	706.0	2356.0	806.4	1322.3	1491.6	854.6	1805.8	1612.1	2348.6	769.2	1943.4
Melanocytes	1627.8	869.8	900.7	1656.3	1902.3	889.2	1344.7	1064.7	1248.8	1251.0	771.1	746.7
Memory B-cells	986.7	317.2	1347.9	1076.4	1369.7	1693.8	703.2	1591.0	1788.4	1535.1	1189.5	1218.8
Mesangial cells	2062.8	386.6	2332.2	657.9	2162.4	2304.7	944.1	2418.2	1918.4	2304.5	1412.2	1328.6
Monocytes	760.1	316.5	1184.7	3917.4	1373.2	1153.4	2748.2	1318.0	1163.0	1114.9	631.0	1305.2
Myocytes	1724.9	861.0	2069.6	1008.9	1787.0	1853.0	925.9	1858.7	1674.1	1590.5	1389.4	1392.0
NK cells	1307.1	852.1	1895.7	1590.7	1478.8	1624.2	989.1	1693.3	1455.6	1723.8	1213.0	1228.8
NKT	1628.8	116.7	1812.7	2348.0	2173.5	1705.7	1366.2	1860.1	1593.9	1808.4	967.9	1266.6

Neurons	1157.7	31.3	1976.6	721.3	1757.1	1649.8	324.3	1978.4	1770.1	1794.6	1176.9	1482.1
Neutrophils	2819.3	298.4	2175.6	4840.3	2171.5	2224.5	4053.9	2427.5	2250.9	2276.3	2060.3	2644.3
Osteoblast	491.8	1282.9	521.5	423.7	813.9	344.3	866.7	468.0	660.4	492.9	1232.9	800.1
Pericytes	2119.0	744.9	1956.1	1032.5	2028.0	2167.5	1624.9	2115.2	1663.7	2073.8	1589.9	1438.1
Plasma cells	1082.6	1127.7	638.9	1410.0	1800.8	759.1	1290.9	637.9	1012.5	1022.2	563.7	834.6
Platelets	400.7	745.6	1495.1	295.5	955.7	1211.6	712.2	1241.7	1073.0	1445.7	595.1	1279.2
Preadipocytes	1107.9	1303.0	1250.6	1067.3	1647.7	932.2	1221.8	1176.4	1261.7	1096.6	1229.3	1571.7
Sebocytes	2133.3	1521.1	1830.3	818.2	1850.6	2148.9	2633.9	1742.4	1972.4	1869.8	1424.0	1673.2
Skeletal muscle	2316.4	442.7	3081.7	374.0	3077.0	3113.8	678.6	3403.1	2825.3	3226.5	1972.0	2080.3
Smooth muscle	1031.3	2121.7	907.9	1679.2	925.2	1071.5	1558.4	1085.1	967.4	868.2	1759.8	1909.0
Tgd cells	417.0	396.2	552.8	617.4	396.3	739.1	373.6	702.1	456.8	763.0	485.2	168.1
Th1 cells	2121.9	1658.2	1293.7	1178.6	1524.3	1095.0	964.7	1423.8	2189.8	1274.5	1710.2	999.3
Th2 cells	1757.6	329.2	2845.8	716.4	2671.5	2876.9	560.0	2243.0	2232.5	2096.0	931.2	1477.3
Tregs	1811.2	1173.3	1875.4	2179.5	1943.2	2130.4	1469.7	1895.6	2048.3	2424.7	1193.6	2171.0
aDC	2170.5	376.1	3389.8	3306.2	3011.7	3421.0	1383.5	3614.7	2949.6	3539.2	2578.5	1947.0
cDC	1487.6	522.4	3570.7	2285.6	3222.9	3049.9	1086.3	3511.1	2988.4	3042.4	2718.2	1680.1
iDC	2884.4	582.6	4403.6	1347.3	4063.9	4375.2	500.1	4830.7	4355.6	4298.4	3255.1	2610.1
ly Endothelial cells	2375.5	838.0	2375.5	910.2	1957.2	1412.7	1527.3	1739.3	2676.0	1866.7	1046.3	1194.3
mv Endothelial cells	1307.5	619.4	1004.2	451.2	819.5	576.3	998.9	748.5	1015.5	674.8	634.0	762.6
naive B-cells	1495.3	466.4	1462.4	1190.6	878.6	1302.1	825.1	1152.4	1783.4	1085.1	1084.8	1596.1
pDC	1866.9	584.0	1873.9	1638.9	1766.6	2061.0	1178.5	2197.5	2120.1	2111.0	1553.7	1443.0
pro B-cells	1625.9	528.3	2018.7	159.8	1826.8	2162.0	279.9	2163.5	1806.7	2029.9	1291.8	1332.5

Supplementary figure S1: Combined pathway analysis for common, immune and ribosome regulating TFs.

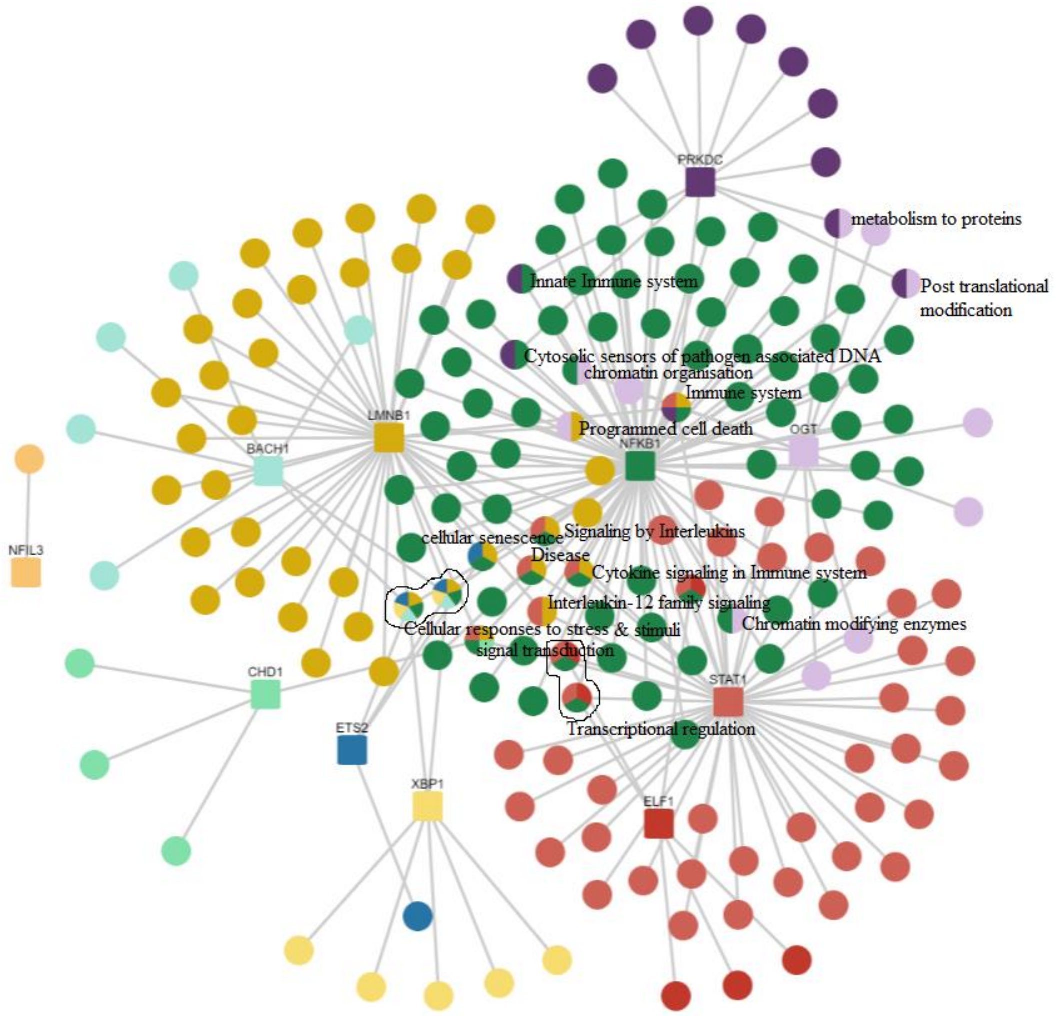

Supplement: Supplemental file 1 — Supplemental material. Download spectrum.04292-22-s0001.pdf, PDF file, 1.1 MB [file spectrum.04292-22-s0001.pdf]
